# Integrated multi-omics characterization reveals a distinctive metabolic signature and the role of NDUFA4L2 in promoting angiogenesis, chemoresistance, and mitochondrial dysfunction in clear cell renal cell carcinoma

**DOI:** 10.18632/aging.101685

**Published:** 2018-12-11

**Authors:** Giuseppe Lucarelli, Monica Rutigliano, Fabio Sallustio, Domenico Ribatti, Andrea Giglio, Martina Lepore Signorile, Valentina Grossi, Paola Sanese, Anna Napoli, Eugenio Maiorano, Cristina Bianchi, Roberto A. Perego, Matteo Ferro, Elena Ranieri, Grazia Serino, Lauren N. Bell, Pasquale Ditonno, Cristiano Simone, Michele Battaglia

**Affiliations:** 1 Department of Emergency and Organ Transplantation- Urology, Andrology and Kidney Transplantation Unit, University of Bari, Bari, Italy; 2 Department of Basic Medical Sciences, Neuroscience and Sense Organs, University of Bari, Bari, Italy; 3 Department of Biomedical Sciences and Human Oncology, Medical Genetics, University of Bari, Bari, Italy; 4 Department of Emergency and Organ Transplantation, Pathology Unit, University of Bari, Bari, Italy; 5 Department of Health Sciences, School of Medicine and Surgery, University of Milano Bicocca, Milan, Italy; 6 Division of Urology, European Institute of Oncology, Milan, Italy; 7 Department of Medical and Surgical Sciences, Molecular Medicine Center, Section of Clinical Pathology, University of Foggia, Foggia, Italy; 8 National Institute of Gastroenterology, ‘S de Bellis’, Castellana Grotte, Bari, Italy; 9 Metabolon, Inc., Research Triangle Park, Morrisville, NC 27560, USA; * Equal contribution

**Keywords:** renal cell carcinoma, metabolomics, transcriptome, NDUFA4L2, mitochondria

## Abstract

An altered metabolism is involved in the development of clear cell - renal cell carcinoma (ccRCC), and in this tumor many altered genes play a fundamental role in controlling cell metabolic activities. We delineated a large-scale metabolomic profile of human ccRCC, and integrated it with transcriptomic data to connect the variations in cancer metabolism with gene expression changes. Moreover, to better analyze the specific contribution of metabolic gene alterations potentially associated with tumorigenesis and tumor progression, we evaluated the transcription profile of primary renal tumor cells. Untargeted metabolomic analysis revealed a signature of an increased glucose uptake and utilization in ccRCC. In addition, metabolites related to pentose phosphate pathway were also altered in the tumor samples in association with changes in Krebs cycle intermediates and related metabolites. We identified NADH dehydrogenase (ubiquinone) 1 alpha subcomplex 4-like 2 (NDUFA4L2) as the most highly expressed gene in renal cancer cells and evaluated its role in sustaining angiogenesis, chemoresistance, and mitochondrial dysfunction. Finally, we showed that silencing of NDUFA4L2 affects cell viability, increases mitochondrial mass, and induces ROS generation in hypoxia.

## Introduction

Renal cell carcinoma (RCC) is the twelfth most common cancer in the world, with over 330,000 new cases diagnosed every year. Recent estimates have calculated that in 2018, in the United States 65,340 new cases will be diagnosed and 14,970 patients will die of RCC [1].

Although the pathogenesis of RCC is still not fully understood, recent data have confirmed that RCC is fundamentally a metabolic disease. In fact, many studies have suggested that an altered metabolism is involved in the development of RCC, and that in this tumor many altered genes play a fundamental role in controlling cell metabolic activities [2].

Moreover, the recent re-discovery of cancer as a metabolic disorder has led to the identification of specific oncometabolites with an important role in tumor growth and progression, such as choline and sarcosine for prostate cancer, or 2-hydroxyglutarate for gliomas [3–5].

The recent comprehensive molecular characterization of clear cell RCC (ccRCC) by the cancer genome atlas (TCGA) research network has confirmed that a reprogrammed metabolism and DNA epigenetic changes, associated with mutations of chromatin remodeling genes, are two major features in this cancer [6]. In particular, these studies have revealed a metabolic reprogramming in ccRCC resulting in a downregulation of the tricarboxylic acid (TCA) cycle genes, upregulation of the pentose phosphate pathway (PPP) genes, and a decreased AMPK expression in high grade, advanced stage disease [6,7].

In addition, the introduction of high-throughput omics technologies has led not only to a detailed molecular characterization of RCC, but also to the identification of biomarkers that allow a more accurate prognostic stratification [7,8]. The discovery of diagnostic and prognostic markers will play a prominent role in this disease considering that up to 30% of cases are diagnosed at an advanced stage and to date we have no specific molecular factor that may help to guide therapy [9]. Recently we showed that in ccRCC a metabolic reprogramming occurs, involving the glucose metabolism and the pentose phosphate pathway, and that patients with high levels of glycolytic enzymes had reduced progression-free and cancer-specific survival rates as compared to subjects with low levels [10,11]. Moreover, we found that glucose and lipid metabolism rearrangements were grade-dependent, suggesting the opportunity of reclassifying ccRCC also on the basis of specific metabolic pathway alterations [12].

In this study we delineated a large-scale metabolomic profile of human ccRCC, and integrated it with transcriptomic data to connect the variations in cancer metabolism with gene expression changes. Moreover, to better analyze the specific contribution of metabolic gene alterations potentially associated with tumorigenesis and tumor progression, we evaluated the transcription profile of primary renal tumor cells, and identified NADH dehydrogenase (ubiquinone) 1 alpha subcomplex 4-like 2 (NDUFA4L2) as the most highly expressed gene in renal cancer cells. We evaluated the prognostic role of NDUFA4L2 and analyzed its functions in sustaining angiogenesis, chemoresistance, and mitochondrial dysfunction.

## RESULTS

### Global metabolic profile distinguishes ccRCC from normal renal tissue

**Figure 1:**
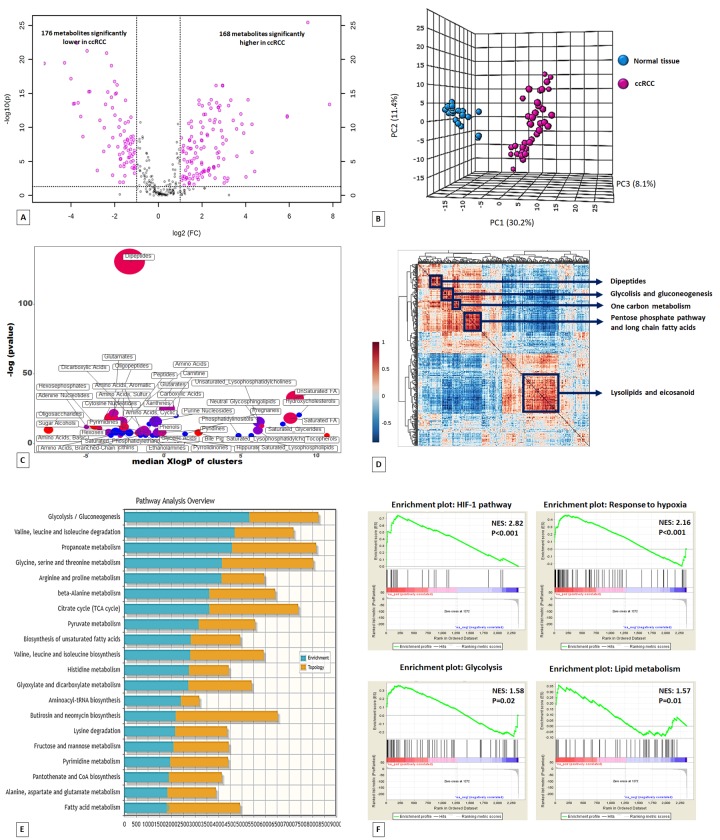
Volcano plot of the 516 metabolites profiled. 344 exhibited significant differential abundance when comparing ccRCC to normal kidney tissues (**A**). Principal component analysis (PCA) of global tissue metabolome demonstrated that the two groups (ccRCC vs normal renal tissue) were clearly distinguishable (**B**). ChemRICH set enrichment statistical plot. Each node reflects a significantly altered cluster of metabolites. Node sizes represent the total number of metabolites in each cluster set. The node color scale shows the proportion of increased (red) or decreased (blue) compounds in tumor compared to normal tissue. Purple color nodes have both increased and decreased metabolites (**C**). Hierarchical cluster analysis and heatmap of metabolite-metabolite correlation matrix. Metabolite clusters are indicated (**D**). Integrated metabolic pathway enrichment analysis. The stacked bars show a summary of the joint evidence from enrichment and topology analyses (**E**).Gene Set Enrichment Analysis (GSEA) of the GSE47032 dataset (**F**).

Untargeted metabolomic analysis was performed on 60 kidney-derived tissues, including 20 normal tissues and 40 ccRCC (Supplementary Table 1), using LC-MS and GC-MS platforms. In total, 516 metabolites were identified, and 344 were found to be differentially expressed in tumor tissues compared to normal samples (168 higher and 176 lower) (Figure 1A). The application of principal component analysis (PCA) to separate normal and pathological samples as a function of the global tissue metabolome demonstrated that the two groups were clearly distinguishable (Figure 1B).

To obtain a global overview of altered biochemical processes, we performed a metabolite set enrichment analysis (MSEA) using MetaboAnalyst 3.0 [13], and an alternative enrichment analysis based on chemical similarity (ChemRICH) [14]. These functional approaches showed that the lipid metabolism and alterations in glucose utilization had the highest impact in ccRCC metabolism (Figure 1C; Supplementary Figures 1 and 2; Supplementary Table 2).

To gain additional insight into cellular metabolites status, we also employed a metabolite–metabolite correlation approach. The correlation heatmap identified 5 main blocks including highly correlated metabolites in the following compound classes: lipids, glucose metabolism-related compounds, pentose phosphate pathway metabolites, one carbon metabolism, and dipeptides (Figure 1D).

### Metabolic perturbations of glucose metabolism in clear cell RCC

**Figure 2:**
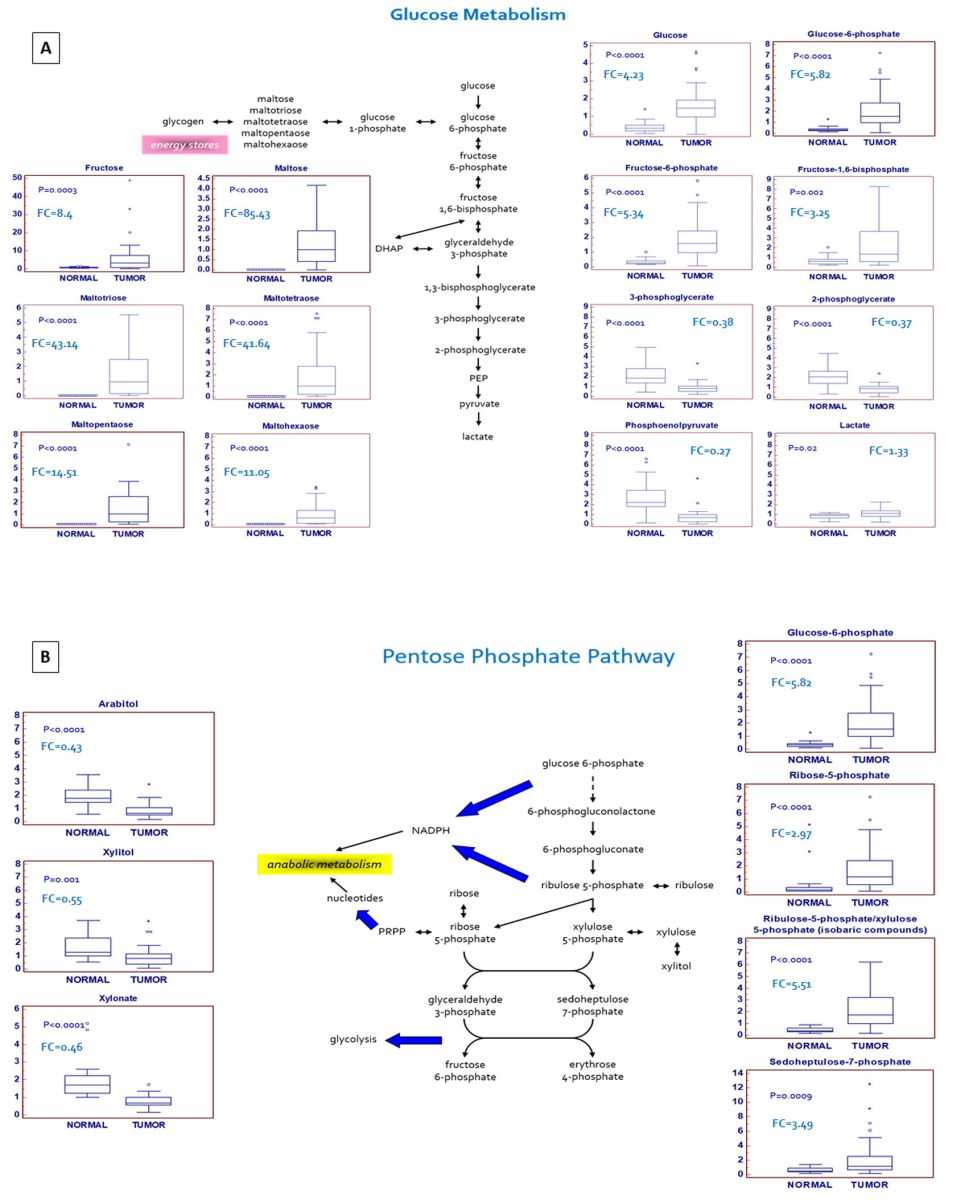
Schematic model summarizing the differences in glucose metabolism (**A**) and pentose phosphate pathway (**B**) between normal and tumor tissue.

A hallmark of many cancers is an increased glucose utilization through glycolysis with lactate production regardless of oxygen availability (Warburg effect). A clear signature of an increased glucose uptake (glucose transporter GLUT1 was over-expressed in ccRCC, Supplementary Figure 3) and utilization was observed in tumor samples, glucose being significantly elevated, along with higher levels of other sugars (fructose and sorbitol) and their phospho-derivatives. Glycogenolysis was also activated in ccRCC, and significant elevations in glycogen synthesis/degradation products were observed. In particular, maltose, maltotriose, maltotetraose, maltopentaose, and maltohexaose were significantly elevated (ranging from 11-fold to 85-fold higher) in tumor tissue. The increased glucose availability was accompanied by elevations in upstream glycolytic intermediates (glucose 6-phosphate, fructose 6-phosphate and fructose 1,6-bisphosphate), reductions in downstream intermediates (3-phosphoglycerate, 2-phosphoglycerate, and phosphoenolpyruvate) and increased lactate production. Moreover, the higher levels of glucose 6-phosphate and fructose 6-phosphate, associated with the increased tissue level of ribulose 5-phosphate, suggested an increased shunting of the metabolites of the upper part of glycolysis into the pentose phosphate pathway (Figure 2A).

### Alterations in pentose phosphate pathway (PPP) activity

In addition to the observed changes in glycolytic intermediates, metabolites related to PPP activity were also altered in the tumor samples. The PPP supports key aspects of accelerated tumor growth, generating precursors for nucleotides synthesis and NADPH for anabolic reactions and redox homeostasis.

An increased glucose utilization through this pathway was observed in ccRCC tissue, as shown by higher levels of the intermediates sedoheptulose 7-phosphate, ribose 5-phosphate, and ribulose 5-phosphate/xylulose 5-phosphate (isobaric compounds). Interestingly, the tissue levels of several pentitols produced from these intermediates, such as arabitol, xylitol, and xylonate, were significantly reduced in the pathological samples, suggestive of an increased PPP activity serving to generate nucleotides (Figure 2B). In addition to supporting nucleotide biosynthesis, an increased NADPH generation through the PPP is important to supply the reducing power necessary for lipid biosynthesis and to maintain the cellular antioxidant glutathione in its reduced state. In particular, the difference in metabolites production between upper and lower glycolysis, in association with the upregulation of glucose-6-phosphate dehydrogenase (G6PDH), suggests that the metabolic flux through this pathway may be differentially partitioned [15]. In fact, while the sugars produced in the upper part of glycolysis are diverted to the PPP to promote both anabolic reactions and redox homeostasis, the triose phosphates generated in the lower part are rerouted towards the TCA cycle and one-carbon metabolism.

### Changes in tricarboxylic acid (TCA) cycle intermediates and related metabolites

**Figure 3:**
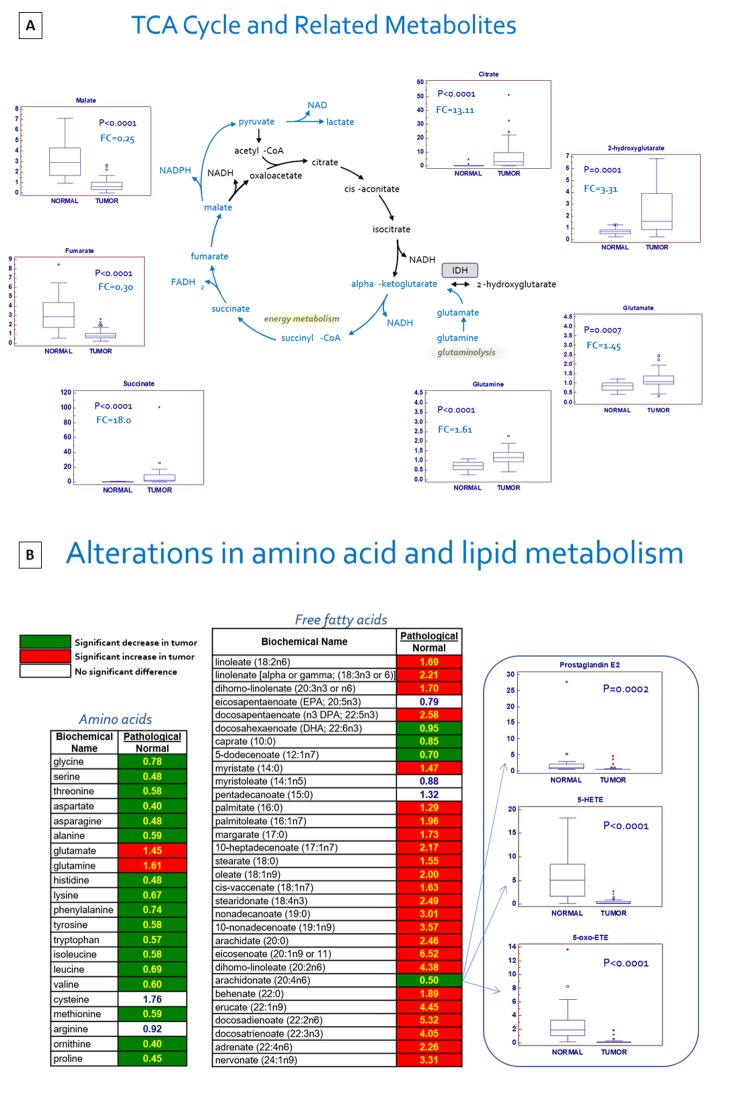
Schematic model summarizing the differences in tricarboxylic acid (TCA) cycle metabolites between normal and tumor tissue (**A**). Alterations in amino acid and lipid metabolism (**B**).

Through glycolysis the glucose metabolism contributes to an increased production of the TCA cycle intermediate, citrate, which can be exported to the cytoplasm and used for lipid synthesis. In addition, many cancer cells utilize the amino acids glutamine and glutamate via glutaminolysis to replenish the TCA cycle. In cancer tissues, citrate and succinate were markedly increased but fumarate and malate were both decreased, suggestive of a metabolic signature that may be consistent with an increased glutaminolysis, because pyruvate levels were maintained and lactate was elevated. In fact, nearly all the amino acids were significantly reduced in tumor tissue, with the exception of glutamine and glutamate, which were significantly elevated (Figure 3A). Taken together, these findings suggest that mitochondrial bioenergetics and oxidative phosphorylation processes are impaired in ccRCC. In addition, the different expression of TCA metabolites, in association with increased levels of glutamine, indicates the activation of reductive carboxylation in ccRCC.

An important metabolic adaptation observed in tumor cells is the reported increased lipid biosynthesis for cellular proliferation, membrane formation and cell signaling. In particular, *de novo* lipogenesis and cholesterogenesis are sustained by conversion in the cytosol of citrate to acetyl-CoA by ATP citrate lyase (ACLY). In this context, the abundance of citrate in ccRCC offers a fundamental substrate for the lipogenesis and lipid metabolism changes observed in this tumor (see below).

In addition to changes in energetics, the significant increase in the oncometabolite 2-hydroxyglutarate (2-HG) is consistent with the findings of a recent study that demonstrated that the increased level of 2-HG in ccRCC was associated with reduced levels of 5-hydroxymethylcytosine (5hmC) in genomic DNA. These results are in accordance with the ability of 2-HG to inhibit TET enzymatic activity [16].

### *Alterations in redox homeostasis*

Due to a consistently elevated metabolic rate, cancer cells are generally exposed to high amounts of oxidative stress. In ccRCC tumor samples, the accumulation of several powerful antioxidants, including alpha-tocopherol (P<0.0001), beta-tocopherol (P<0.0001), gamma-tocopherol (P<0.0001), ascorbate (vitamin C) (P=0.0097), and ergothioneine (P=0.0001), was observed. The levels of the powerful cellular antioxidant glutathione (P=0.01) were increased too. Depletion of the glutathione precursors methionine and homocysteine, and maintenance of cysteine levels despite significant reductions in nearly all the other amino acids (except glutamine and glutamate) is suggestive of an increased glutathione synthesis in an attempt to maintain redox homeostasis. In addition, the levels of the glutathione degradation product 5-oxoproline (P=0.0007) were significantly reduced in cancer tissues.

### *Changes in amino acids, lipids metabolism and other metabolites*

In addition to the affected biochemical pathways described above, alterations in amino acid/protein and lipid metabolism were prevalent in ccRCC tissue, as evidenced by significant reductions in nearly all the free amino acids measured, and significant elevations in many dipeptides (Figure 3B). Taken together, these changes may be indicative of a dynamic gene expression program that includes protein degradation and the utilization of amino acids for protein synthesis in cancer cells. In addition, massive changes in nearly all the free fatty acids measured were noted, and an accumulation of many essential and long-chain fatty acids was observed in pathological samples. These changes may be associated with an increased fatty acid uptake and/or synthesis, likely for subsequent membrane biosynthesis to promote cellular growth and proliferation. The observed changes in the glycerolipid metabolism, including the membrane precursors/degradation products choline, ethanolamine, choline phosphate, phosphoethanolamine, cytidine 5’-diphosphocholine, glycerol, and glycerolphosphorylcholine, provide further support for the notion of an active membrane remodeling occurring in ccRCC tumor tissue. Levels of many lysolipids, which are similar to phospholipids in that they contain a head group, a phosphate group, and a glycerol backbone, but they only have a single esterified fatty acyl chain, were altered in tumor samples as well, again suggestive of an increased membrane turnover. Significant elevations in the polyamines putrescine (P<0.0001) and spermidine (P=0.02), which bind to and stabilize DNA to promote cellular proliferation, were also observed in ccRCC samples. A mixed signature of inflammation was noted in pathological samples as levels of the arachidonate (20:4n6)-derived inflammatory markers prostaglandin E2, 5-HETE, and 5-oxoETE were significantly reduced. This may be simply due to depletion of arachidonate and therefore the lack of this precursor for eicosanoid synthesis. In contrast, kynurenine, a metabolite of the amino acid tryptophan, was elevated nearly 6-fold in ccRCC tumor tissue [17].

Finally, significant elevations in heme (P=0.001), bilirubin (P=0.0001), and biliverdin (P<0.0001) were suggestive of an altered heme metabolism in tumor tissue. Indeed, an increased activity of the enzyme heme oxygenase (HO), which converts heme to biliverdin and releases iron, has been implicated in various aspects of tumor biology including the induction of angiogenesis and resistance to chemotherapy [18].

### Integrated metabolomics/transcriptomic signature

To compare the relative changes in gene expression and metabolite abundance in ccRCC, we integrated the metabolomics data with gene expression data from 10 ccRCC tumor samples and matched non-tumor kidney tissues samples obtained from patients who underwent nephrectomy in our department (GSE47032). The combined analysis identified 20 significantly enriched biochemical pathways (p<0.05), including those of glycolysis and fatty acid metabolism (Figure 1E).

### Human renal tumor cells display an altered expression profile of metabolic and damaged mitochondria-associated genes

Gene Set Enrichment Analysis (GSEA) [19] of the GSE47032 dataset showed that ccRCC featured multiple enriched gene sets depicting HIF-1 and hypoxia pathway activation, glycolysis and an enhanced lipid metabolism (Figure 1F).

To gain further insight into the specific contribution of renal cancer cells to the global metabolic profile of ccRCC, 5 clones of normal renal tubule epithelial cells and 6 clones of primary renal cancer cells, derived from different patients, were used for genome-wide gene expression profiling by oligonucleotide microarray (Dataset GSE117890). Primary renal cancer cells showed a distinct gene expression profile, with 302 differentially expressed genes. In this aberrant gene expression signature, we found the overexpression of molecules involved in cell metabolism such as PTG/PPP1R3C (involved in glycogen metabolism), CA IX and XII (pH homeostasis), ENO2 and PFKFB4 (glucose metabolism), as well as ACLY and HILPDA (lipogenesis and cholesterogenesis). Interestingly, NADH dehydrogenase (ubiquinone) 1 alpha sub complex 4-like 2 (NDUFA4L2, Gene ID: 56901), was the most highly expressed gene in cancer cells compared with normal tubular cells (absolute FC = 86.180; Log FC = 6.43). This gene was also highly expressed in the GSE47032 dataset. To confirm the above findings, we analyzed the differential expression of NDUFA4L2 mRNA by data mining of the Oncomine microarray gene expression datasets (Supplementary Table 3). In accordance with our results, NDUFA4L2 was significantly upregulated in all the ccRCC datasets (global P=1.30 E-11). In addition, exploration of the Metabologram Data Portal showed the abundance of succinate, reduced levels of malate and fumarate, and the upregulation of NDUFA4L2 in tumor tissues (Supplementary Figure 4). These findings are consistent with a reduced oxidative phosphorylation rate in ccRCC.

NDUFA4L2 is a HIF-1 target gene encoding for a regulatory protein that attenuates mitochondrial oxygen consumption through the inhibition of Complex I activity [20]. The gene expression profile also showed the overexpression of two genes (SPATA18 and ATG9A) encoding for proteins that are recruited to damaged mitochondria to mediate mitochondrial quality control mechanisms [21,22].

In addition, we observed a reduced expression of the RUVBL complex genes (RUVBL1 and ACTL6A). RUVBL1, and its interacting protein ACTL6A, have been shown to respond to the cell’s metabolic state, regulate mTORC1 signaling activity, and they are positively correlated with transcripts encoding proteins with a mitochondrial function [23]. Finally, a reduced expression of genes encoding mitochondrial ribosomal proteins (MRPS9 and MRPL3) was found (Supplementary Figure 3). Gene Ontology (GO) enrichment analysis performed using ClueGO [24] showed how HIF-mediated metabolic pathways and mitophagy were the most significantly overrepresented biological processes in renal cancer cell (Supplementary Figure 6).

**Figure 4:**
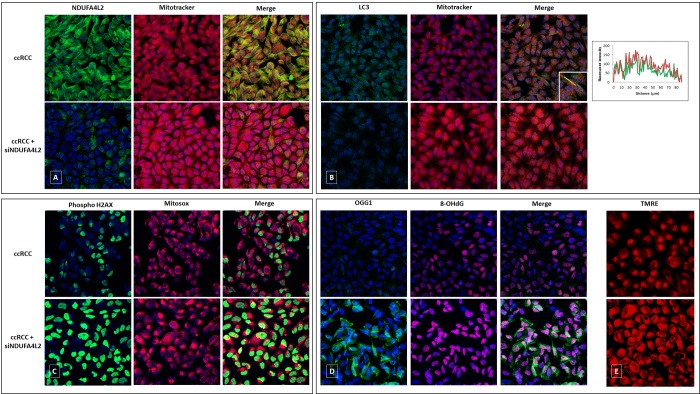
Immunofluorescence showing the increase in MitoTracker signal levels in ccRCC cells treated with small interfering RNA targeting NDUFA4L2 (siNDUFA4L2) compared to untreated cells (**A**). The autophagic marker LC3 is increased in ccRCC cells and co-localizes with the mitochondrial label MitoTracker. A line profile is shown (**B**). NDUFA4L2-silenced cancer cells show an increased superoxide radicals production (**C**), high levels of phospho-H2AX (**C**), a significant accumulation of 8-oxodG (**D**), and a reactive increased expression of the DNA repair enzyme OGG1 (**D**). NDUFA4L2 silencing increased the membrane potential in cancer cells, as shown by increased signals of the fluorescence probe TMRE (**E**).

**Figure 5:**
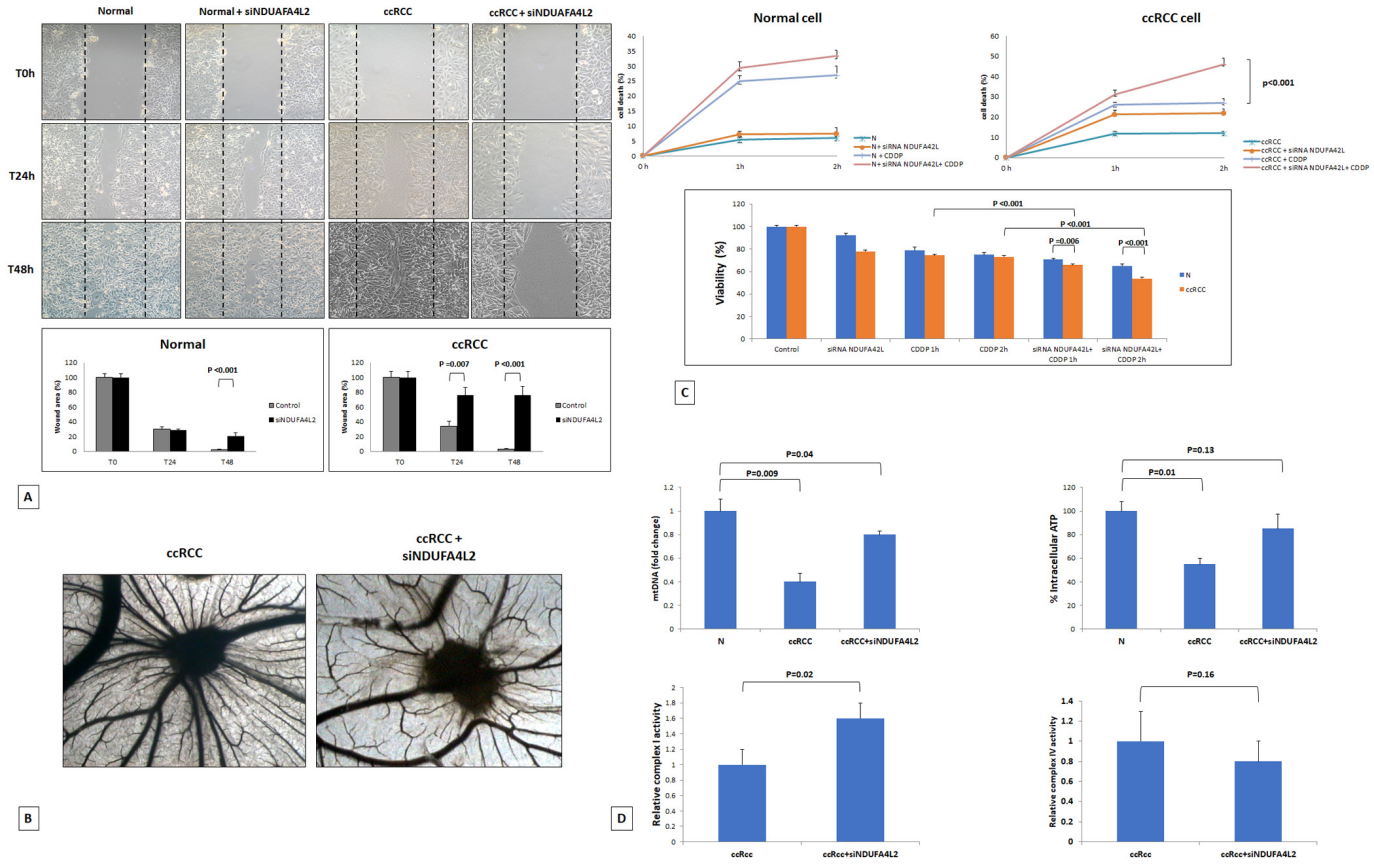
Wounded normal and tumor cell monolayers were photographed 24 and 48 hours after the mechanical scratch and the area of the wounds was measured in 3 independent wound sites per group. When specified, the cells were treated with small interfering RNA targeting NDUFA4L2 (siNDUFA4L2). RCC cells treated with siNDUFA4L2 have decreased cell migratory capabilities compared with untreated tumor cells (**A**). Chick embryo chorioallantoic membrane angiogenic assay: when tumor cell are treated with siNDUFA4L2, a lower vascular reaction is detectable (**B**). NDUFA4L2 has a role in RCC resistance to cisplatin (CDDP)-induced cytotoxicity (**C**). The death rate of treated tumor cells (tumor+ siNDUFA4L2+CDDP) is significantly higher than that of untreated cells (tumor+CDDP) (P<0.001). No difference is observed in normal cells. MTT assay reveals significantly decreased cell viability when RCC cells are treated with siNDUFA4L2 before cisplatin incubation (**C**). RCC cells exhibit reduced levels of mitochondrial DNA, and produced lower levels of ATP, as compared to normal cells. These levels are rescued when cancer cells are treated with siNDUFA4L2 (**D**). NDUFA4L2 specifically inhibits mitochondrial complex I but not complex IV activity (**D**).

The primary ccRCC cell line, in fact, exhibited reduced levels of mitochondrial DNA, mitochondrial mass (MitoTracker Red) and produced lower levels of ATP, as compared to normal cells. These levels were rescued when cancer cells were treated with small interfering RNA (siRNA) targeting NDUFA4L2 (siNDUFA4L2) (Figures 4 and 5). In addition, we observed an increased expression of the autophagic marker LC3, that colocalized with the mitochondrial label MitoTracker (Figure 4B). When NDUFA4L2 was silenced, we observed reduced LC3 levels, and an increase in fluorescence intensity of MitoTracker, suggesting an impaired mitochondrial degradation [25].

Moreover, NDUFA4L2 silencing increased the membrane potential and reactive oxygen species (ROS) production in cancer cells, as shown by increased signals of the fluorescence probe TMRE and the mitochondrial superoxide indicator MitoSOX (Figure 4E).

Since NDUFA4L2-silenced cancer cells showed an increased ROS production, and superoxide radicals can induce histone modifications, we analyzed the phosphorylation of the H2AX histone. In particular, we observed increased phospho-H2AX levels in cancer cells when NDUFA4L2 was silenced, suggesting that NDUFA4L2 plays an important role in ROS control and that its loss generates cell stress (Figure 4C).

Considering that 8-oxo-7,8-dihydro-2’-deoxyguanosine (8-oxodG) is one of the predominant forms of free radical-induced oxidative lesions in DNA damage, and that 8-oxodG is repaired by enzyme 8-oxoguanine-DNA glycosylase (OGG1), we evaluated their expression in NDUFA4L2-silenced renal tumor cells. When NDUFA4L2 was silenced, we observed a significant accumulation of 8-oxodG, as a consequence of a reactivation of oxidative phosphorylation (Figure 5) and ROS production, and a reactive increased expression of the DNA repair enzyme OGG1, that prevents excessive oxidative DNA damage in cancer cells (Figure 4D).

Taken together these findings, in association with the particular metabolomic profile (partitioned metabolic flux through glycolysis), are suggestive of an altered mitochondrial function in ccRCC (impaired mitochondrial bioenergetics and oxidative phosphorylation, and NDUFA4L2 overexpression), and indicate the activation of compensatory mechanisms to keep mitochondrial ROS under control, in association with increased levels of cellular antioxidants (glutathione, tocopherol, and ascorbate).

### NDUFA4L2 expression is increased in neoplastic tissue and is a risk factor for ccRCC progression and mortality

To analyze the transcription levels, we firstly performed quantitative real-time PCR and evaluated NDUFA4L2 mRNA levels in ccRCC tissue samples compared with normal renal parenchyma (Supplementary Figure 5). Normalized gene expression levels for NDUFA4L2 were significantly higher in ccRCC as compared with normal tissues.

**Figure 6:**
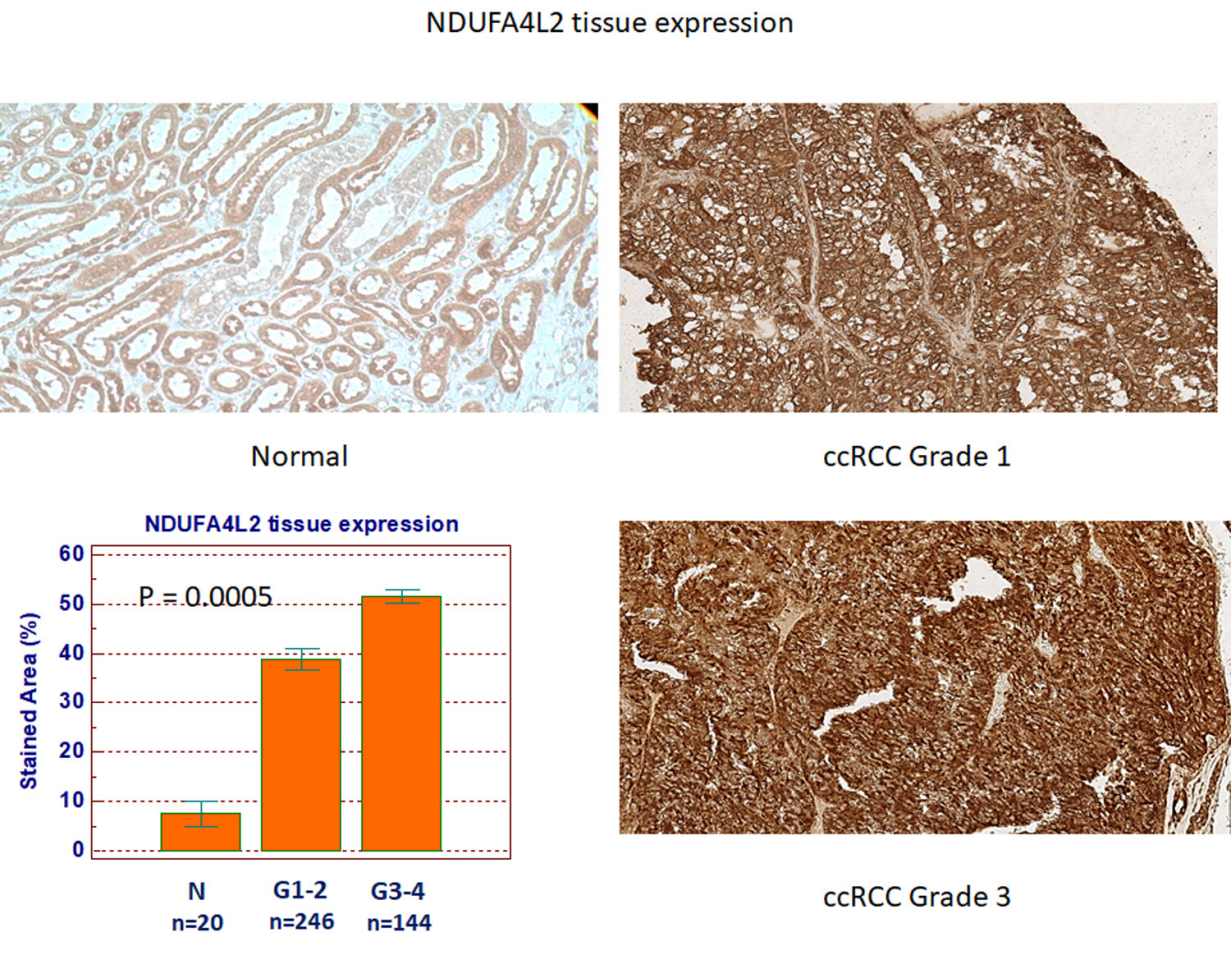
NDUFA4L2 expression in normal (n=20) and ccRCC (n=390) specimens.

**Figure 7:**
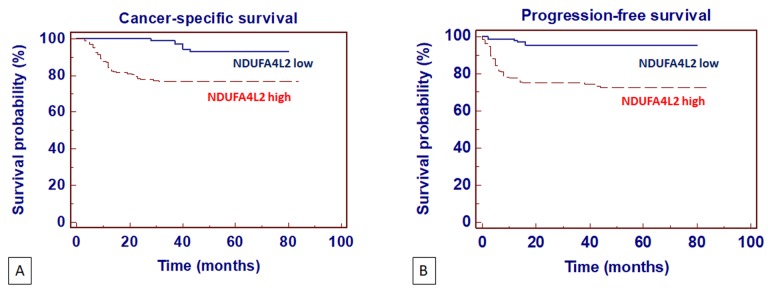
Kaplan-Meier cancer-specific survival (CSS) (**A**) and progression-free survival (PFS) (**B**) curves, stratified by NDUFA4L2 tissue expression levels. Patients with higher NDUFA4L2 levels had reduced CSS and PFS as compared to patients with lower values.

Next, to analyze the expression and location of NDUFA4L2, we performed immunohistochemistry on normal and pathological tissues, eliciting results consistent with the gene expression levels (Figure 6). NDUFA4L2 protein levels were significantly higher in ccRCC than in healthy tissue (P=0.0005). Detailed clinical and pathological characteristics of the patients are summarized in Supplementary Table 4. Kaplan-Meier survival curves for cancer-specific survival (CSS) and progression-free survival (PFS), stratified by NDUFA4L2 tissue expression, are shown in figure 7. Both CSS and PFS were significantly decreased in patients with high levels of NDUFA4L2. Univariate analysis for the predefined variables showed that the pathological stage, presence of nodal and visceral metastases, Fuhrman grade, presence of necrosis, tumor size, and high levels of NDUFA4L2, were significantly associated with the risk of death (Table 1) and progression (Table 2). At multivariate analysis by Cox regression modeling, the pathological stage, presence of nodal and visceral metastases, Fuhrman grade, and increased expression of NDUFA4L2, were independent adverse prognostic factors for CSS and PFS (Tables 1 and 2).

**Table 1:** Univariate and multivariate analyses for cancer-specific survival.

**Variable**	**Category**	**Univariate**		**Multivariate**	
		HR (95% CI)	P-value	HR (95% CI)	P-value
**T stage**	T3 vs T1/2	3.21 (2.69-4.31)	0.001	2.18 (1.22-3.18)	0.01
**N stage**	N+ vs N0	4.12 (3.11-7.65)	0.001	2.35 (1.26-5.38)	0.001
**M stage**	M+ vs M0	6.45 (4.46-11.28)	0.001	4.45 (2.74-8.15)	0.001
**Grade**	G3/4 vs G1/2	2.34 (1.12-5.66)	0.01	1.29 (1.02-2.24)	0.01
**Necrosis**	Yes vs No	2.12 (1.15-3.96)	0.01	-	-
**Tumor size**	Continuous	1.29 (1.03-2.12)	0.01	-	-
**NDUFA4L2**	High vs Low	3.45 (1.14-5.78)	0.001	2.11 (1.06-2.95)	0.001

**Table 2:** Univariate and multivariate analyses for progression-free survival.

**Variable**	**Category**	**Univariate**		**Multivariate**	
		HR (95% CI)	P-value	HR (95% CI)	P-value
**T stage**	T3 vs T1/2	5.43 (2.28-7.85)	0.001	2.89 (1.56-6.43)	0.01
**N stage**	N+ vs N0	3.74 (1.95-6.22)	0.01	2.02 (1.18-5.93)	0.01
**M stage**	M+ vs M0	5.36 (2.22-10.16)	0.001	3.18 (2.01-6.31)	0.001
**Grade**	G3/4 vs G1/2	2.43 (1.19-7.21)	0.01	1.94 (1.21-3.65)	0.01
**Necrosis**	Yes vs No	1.85 (1.04-2.86)	0.01	-	-
**Tumor size**	Continuous	1.88 (1.03-2.86)	0.01	-	-
**NDUFA4L2**	High vs Low	2.28 (1.16-3.18)	0.001	1.96 (1.03-2.28)	0.001

### NDUFA4L2 has a role in cancer cell migration and angiogenesis, and decreases cisplatin-induced renal cancer cell death

To study the role of NDUFA4L2 in renal cancer cell proliferation, migration, angiogenesis and chemoresistance, *in vitro* and *in vivo* assays were performed. The scratch wound healing assay showed that primary ccRCC cells treated with siNDUFA4L2 had a decreased migratory ability compared with normal cells (Figure 5A). To investigate the angiogenic response, suspensions of tumor cells alone, or treated with siRNA, were seeded on the top of the chick embryo chorioallantoic membrane (CAM) and their ability to induce the formation of new vessels was histologically evaluated. In particular, the CAM assay showed that gelatin sponges soaked with the tumor cells suspension were surrounded by numerous allantoic vessels that developed radially toward the implant in a spoked wheel pattern (mean ± SD= 28 ± 4 blood vessels). In contrast, few blood vessels were identified around sponges containing tumor cells treated with siRNA targeting NDUFA4L2 (mean ± SD= 14 ± 3; P = 0.001 vs untreated tumor cells) (Figure 5B).

Next, we evaluated the role of NDUFA4L2 in sustaining cancer cell proliferation and in reducing cisplatin-induced cytotoxicity. While the absence of NDUFA4L2 did not significantly affect cell proliferation in normal renal tubular cells, NDUFA4L2-silenced renal cancer cells proliferated at a slower rate than non-silenced cancer cells. In addition, after cisplatin treatment, the death rate of tumor cells treated with siNDUFA4L2 was significantly greater than that of untreated cancer cells (p < 0.001, Figure 5C). The MTT assay confirmed these findings, demonstrating a decreased cell viability when tumor cells were pre-treated with siNDUAFA4L2 before cisplatin incubation (Figure 5C).

### Silencing of NDUFA4L2 affects cell viability, increases mitochondrial mass, and induces ROS generation in hypoxia

**Figure 8:**
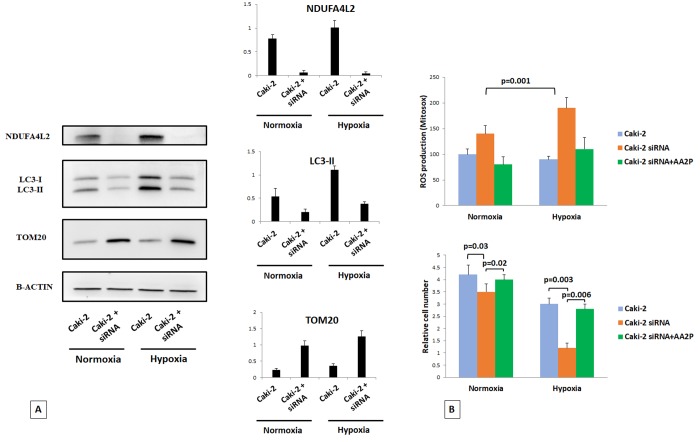
Immunoblot analysis of Caki-2 cells cultured under normoxic (21% O2) or hypoxic (1% O2) conditions for 18 hr and probed against NDUFA4L2, LC3, and TOM20 antibodies. Beta actin was used as a loading control (**A**). The silencing of NDUFA4L2 impairs cancer cell proliferation, inhibits the autophagic machine, and increases the levels of the mitochondrial protein TOM20, especially in hypoxic conditions (**B**). Cell proliferation was restored when NDUFA4L2-silenced cells were pre-treated with ascorbic acid 2-phosphate (AA2P) (**B**).

We used Caki-2 cell lines in normoxic and hypoxic conditions to better analyze the role of NDUFA4L2 in controlling cell proliferation and the autophagic turnover of damaged mitochondria. In normoxic conditions, the silencing of NDUFA4L2 impaired cell proliferation, led to an inhibition of the autophagic machine, and increased the mitochondrial mass, as suggested by higher levels of the mitochondrial protein TOM20 (Figure 8A). These effects were more evident in hypoxia, where the absence of NDUFA4L2 significantly affected renal cancer cell viability. To investigate whether the increased production of ROS in silenced-Caki-2 cells during hypoxic conditions was responsible for the impaired cell viability, we evaluated ROS generation (using the mitochondrial superoxide indicator MitoSOX) and the effects of ascorbic acid 2-phosphate (AA2P) exposure. In NDUFA4L2-silenced cells, during hypoxia we found an overproduction of ROS in association with a significantly reduced cell viability as compared to in normoxic conditions (Figure 8B). Cell proliferation was restored when NDUFA4L2-silenced cells were pre-treated with AA2P, suggesting that an increased mitochondrial ROS generation may be involved in the impaired cell viability observed in hypoxic conditions, as a consequence of a reactivation of oxidative phosphorylation in mitochondria (Figure 8B). These findings were also in accordance with the increased levels of H2AX histone phosphorylation observed in silenced human renal cancer cells, suggesting that the lack of NDUFA4L2 induces cell stress.

## DISCUSSION

Analysis of the ccRCC metabolome and its integration with transcriptomic data revealed significant metabolic alterations in this tumor. In particular, we observed increased levels of metabolites in the upper chain of reactions involving 6-carbon molecules, and a reduction of metabolic intermediates in the lower part of glycolysis, downstream of fructose 6-phosphate. This different distribution of metabolites production between the upper and lower chains of glycolytic reactions suggests that the metabolic flux through this pathway is differentially partitioned. In particular, the sugars generated in the upper part of glycolysis are rerouted to the pentose phosphate pathway, while the triose phosphates produced in the lower part are diverted towards the Krebs cycle or one-carbon metabolism. In this scenario, our results suggest that in ccRCC the mitochondrial bioenergetics and oxidative phosphorylation processes are impaired, and that an increased glucose utilization is promoted through the pentose phosphate pathway. These alterations in glucose metabolism and pentose phosphate pathway were in accordance with our previous findings showing that in ccRCC, oncogenic signaling pathways may promote cancer through rerouting the sugar metabolism [10].

Tello et al. have recently shown that NADH dehydrogenase (ubiquinone) 1 alpha subcomplex 4-like 2 (NDUFA4L2) is a HIF-1 target gene encoding for a protein that attenuates mitochondrial oxygen consumption through inhibiting the electron transport chain (ETC) Complex I [20]. Interestingly, NDUFA4L2 is one of the most highly expressed genes in clear cell RCC, as shown in our study and by data mining of public Oncomine microarray datasets (Supplementary Table 3). We found that NDUFA4L2 had a fundamental role in ccRCC bioenergetics and in different processes such as cell proliferation, cancer cell migration and angiogenesis. RCC is a notoriously chemo-resistant tumor. In the last years, an in-depth understanding of the molecular basis of this tumor has led to introduction of novel targeted therapies, although these agents yield partial responses in a minority of patients, with no evidence of complete responses [9,26]. We hypothesized that NDUFA4L2 over-expression could strengthen drug resistance. To explore this hypothesis renal cancer cells were pretreated with siRNA targeting NDUFA4L2. Interestingly, NDUFA4L2 knockdown decreased cell viability, and improved cisplatin susceptibility, suggesting that this protein can regulate chemotherapy resistance in RCC. Moreover, NDUFA4L2 silencing led to an inhibition of the autophagic machine, increased mitochondrial mass, and induced an overproduction of ROS, especially in hypoxic conditions. In particular, the reduced levels of NDUFA4L2 significantly affected lipidation of LC3 protein, and were associated with concomitant increased expression of the mitochondrial membrane protein TOM20. In accordance with these findings, renal cancer cells treated with siNDUFA4L2, exhibited increased levels of mitochondrial DNA, and increased fluorescence intensity of mitochondrial dye MitoTracker.

Further studies are warranted to delineate the precise role of NDUFA4L2 in regulating angiogenesis and mitophagy.

In conclusion, our findings delineate a clear cell RCC metabolic signature characterized by an anaerobic switch that favors rerouting of the sugar metabolism toward the pentose phosphate pathway, and impairs the mitochondrial activity though the overexpression of NDUFA4L2. Thus, this protein could serve as a marker of ccRCC aggressiveness, as well as a potential novel therapeutic target.

## MATERIALS AND METHODS

### Study population and tissue collection

Investigation has been conducted in accordance with the ethical standards and according to the Declaration of Helsinki and according to national and international guidelines and has been approved by the authors' institutional review board. Written informed consent to take part was given by all participants.

Primary renal tumor (n=40) and non neoplastic tissues (n=20) were collected from patients who underwent radical or partial nephrectomy for ccRCC (Supplementary Table 1). All specimens were immediately used to obtain primary cell cultures after frozen-section confirmation of the diagnosis. Two pathologists confirmed the presence of clear cell RCC in the neoplastic tissues and excluded tumor cells in the healthy specimens. Patients with diabetes mellitus and/or an estimated glomerular filtration rate (MDRD equation) < 60 mL/min/1.73m^2^ were excluded from the study.

For the NDUFA4L2 tissue expression study, tissue samples were collected from 390 patients with ccRCC. Detailed clinical and pathological characteristics of these patients are summarized in Supplementary Table 4. All patients were preoperatively staged by thoraco-abdominal Computed Tomography or Magnetic Resonance Imaging. Tumor staging was reassigned according to the seventh edition of the AJCC-UICC TNM classification. The 2016 World Health Organization and Fuhrman classifications were used to attribute histological type and nuclear grade, respectively.

### Metabolite analysis

### *Sample preparation*

Metabolic profiling of RCC samples was carried out at Metabolon Inc. All tissue samples were maintained at -80 ºC until processed. The sample preparation process was carried out using the Hamilton Company automated MicroLab STAR® system. Recovery standards were added prior to the first step in the extraction process for QC purposes. Sample preparation was conducted using a proprietary series of organic and aqueous extractions to remove the protein fraction, while allowing maximum recovery of small molecules. The resulting extract was divided into two fractions; one for analysis by LC and one for analysis by GC. Samples were placed briefly on a TurboVap® (Zymark) to remove the organic solvent. Each sample was then frozen and dried under vacuum. Samples were then prepared for the appropriate instrument, either LC/MS or GC/MS. For QA/QC purposes, a number of additional samples were included in each day’s analyses. Furthermore, a selection of QC compounds was added to every sample, including those under test. These compounds were carefully chosen so as not to interfere with the measurement of the endogenous compounds. The Supplementary Tables 5 and 6 describe the QC samples and compounds. These QC samples were primarily used to evaluate the control process for each study as well as assisting in data curation.

### *Liquid chromatography/Mass Spectrometry (LC/MS, LC/MS^2^)*

The LC/MS portion of the platform was based on a Waters ACQUITY UPLC and a Thermo-Finnigan LTQ mass spectrometer, which consisted of an electrospray ionization (ESI) source and linear ion-trap (LIT) mass analyzer. The sample extract was split into two aliquots, dried, then reconstituted in acidic or basic LC-compatible solvents, each of which contained 11 or more injection standards at fixed concentrations. One aliquot was analyzed using acidic positive ion optimized conditions and the other using basic negative ion optimized conditions in two independent injections using separate dedicated columns. Extracts reconstituted in acidic conditions were gradient-eluted using water and methanol, both containing 0.1% Formic acid, while the basic extracts, also reconstituted using water/methanol, contained 6.5mM Ammonium Bicarbonate. The MS analysis alternated between MS and data-dependent MS^2^ scans using dynamic exclusion.

### *Gas chromatography/Mass Spectrometry (GC/MS)*

 The samples destined for GC/MS analysis were re-dried under vacuum desiccation for a minimum of 24 hours prior to being derivatized under dried nitrogen using bistrimethyl-silyl-trifluoroacetamide (BSTFA). The GC column was 5% phenyl and the temperature ramp is from 40° to 300° C in a 16 minute period. Samples were analyzed on a Thermo-Finnigan Trace DSQ fast-scanning single-quadrupole mass spectrometer using electron impact ionization. The instrument was tuned and calibrated for mass resolution and mass accuracy on a daily basis. The information output from the raw data files was automatically extracted as discussed below.

### *Accurate Mass Determination and MS/MS fragmentation (LC/MS), (LC/MS/MS)*

The LC/MS portion of the platform was based on a Waters ACQUITY UPLC and a Thermo-Finnigan LTQ-FT mass spectrometer, which had a linear ion-trap (LIT) front end and a Fourier transform ion cyclotron resonance (FT-ICR) mass spectrometer backend. For ions with counts exceeding 2 million, an accurate mass measurement could be performed. Accurate mass measurements could be made on the parent ion as well as fragments. The typical mass error was less than 5 ppm. Characterizing ions with less than two million counts is a more laborious process. Fragmentation spectra (MS/MS) were typically generated in a data-dependent manner but if necessary, targeted MS/MS could be employed, as in the case of lower level signals.

### *Laboratory Information Management System (LIMS)*

The informatics system consisted of four major components, the Laboratory Information Management System (LIMS), the data extraction and peak-identification software, data processing tools for QC and compound identification, and a collection of information interpretation and visualization tools for use by data analysts. The hardware and software foundations for these informatics components were the LAN backbone, and a database server running on the Oracle 10.2.0.1 Enterprise Edition. The purpose of the Metabolon LIMS system was to enable fully auditable laboratory automation through a secure, easy to use, and highly specialized system. The scope of the Metabolon LIMS system encompasses sample accessing, sample preparation and instrumental analysis and reporting and advanced data analysis. All of the subsequent software systems are grounded in the LIMS data structures, modified for leverage and interface with the in-house information extraction and data visualization systems, as well as third party instrumentation and data analysis software.

### *Data extraction and quality assurance*

The data extraction of the raw mass spec data files yielded information that could be loaded into a relational database and manipulated without resorting to BLOB manipulation. Once in the database, the information was examined and appropriate QC limits were imposed. Peaks were identified using Metabolon’s proprietary peak integration software, and component parts were stored in a separate, specifically designed complex data structure.

### *Compound identification*

Compounds were identified by comparison to library entries of purified standards or recurrent unknown entities. The identification of known chemical entities was based on comparison to metabolomic library entries of purified standards. As of this writing, more than 1000 commercially available purified standard compounds had been acquired and registered into LIMS for distribution to both the LC and GC platforms for determination of their analytical characteristics. The combination of chromatographic properties and mass spectra indicated a match to the specific compound or an isobaric entity. Additional entities could be identified by virtue of their recurrent nature (both chromatographic and mass spectral). These compounds may potentially be identified by future acquisition of a matching purified standard or by classical structural analysis.

### *Curation*

A variety of curation procedures was carried out to ensure that a high quality data set was made available for statistical analyses and data interpretation. The QC and curation processes were designed to ensure an accurate and consistent identification of true chemical entities, and to remove those representing system artifacts, mis-assignments, and background noise.

Metabolon data analysts use proprietary visualization and interpretation software to confirm the consistency of peak identification among the various samples. Library matches for each compound were checked for each sample and corrected if necessary.

### *Normalization*

For studies spanning multiple days, a data normalization step was performed to correct variations resulting from inter-day instrument tuning differences. Essentially, each compound was corrected in run-day blocks by registering the medians to equal one (1.00) and normalizing each data point proportionately. For studies that did not last more than one day, no normalization is necessary, other than for purposes of data visualization.

### *Bioinformatics and statistical analyses*

Significance tests were performed with MedCalc 9.2.0.1 (MedCalc software, Mariakerke, Belgium) and “R” (http://cran.r-project.org). Global biochemical profiles were determined in human kidney tissue/tumor samples and compared across groups stratified by renal tissue pathology status. An estimate of the false discovery rate (q-value) was calculated to take into account the multiple comparisons that normally occur in metabolomic-based studies. Comparisons of metabolites median values between different groups were evaluated by Mann–Whitney U test. In the cancer-specific survival (CSS) analysis, patients who died of RCC-unrelated causes or were lost to follow-up were censored. Progression-free survival (PFS) was calculated from the date of surgery to the date of disease recurrence. Estimates of CSS and PFS were calculated according to the Kaplan–Meier method and compared with the log-rank test. Univariate and multivariate analyses were performed using the Cox proportional hazards regression model to identify the most significant variables for predicting CSS and PFS. A backward selection procedure was performed with removal criterion P > 0.10 based on likelihood ratio tests. A P-value of <0.05 was considered statistically significant.

### *Integration of metabolomic and transcriptomic data*

Clear cell-RCC transcriptome data derived from exon array analysis of 20 total samples (10 ccRCC tumor sample and their matched non-tumor kidney tissues samples) were used. Exon array data are deposited in GEO at Series accession number GSE47032.

MetaboAnalyst 3.0 [13] was used for the metabolite set enrichment (MSEA) and to integrate the data from the transcriptomics and metabolomics experiments. The main function of this module is to pinpoint the pathways involved in the underlying biological processes by combining the evidence based on alterations in both gene expression and metabolite concentrations. In addition, a topology analysis was used to evaluate the relative importance of the gene/compounds based on their relative locations within a pathway. Over-representation analysis of the pathways was based on the hypergeometric test.

Gene set enrichment analysis (GSEA) [19] was used to determine which pathways were statistically enriched across the renal cancer data sets. The normalized enrichment score (NES) was used to evaluate the extent and direction of enrichment of each pathway.

Biochemical pathway enrichment analysis was performed using ChemRICH [14], a novel statistical approach based on chemical similarity that yields study-specific, non overlapping sets of all identified metabolites. Enrichment p-values were obtained with the Kolmogorov-Smirnov test.

Finally, Gene Ontology (GO) enrichment analysis was processed by the ClueGO 2.3.2 plug-in of Cytoscape [24], which integrates GO terms as well as KEGG pathways and creates a functionally organized GO/pathway term network.

### Primary cell cultures from renal tissues

Tumor (ccRCC) and normal (N) kidney tissue specimens were immediately placed in a Petri dish with phosphate-buffered saline (PBS) 1x and cut into small pieces of about 1 mm^3^. Each small piece of tissue was placed on the surface of the Petri dish with Dulbecco’s Modified Eagle Medium (DMEM, Invitrogen, Life Technologies, Monza, Italy) supplemented with 10% fetal bovine serum (FBS, Sigma-Aldrich, Milan, Italy) and 1% penicillin-streptomycin-L-glutamine (Sigma-Aldrich, Milan, Italy). Cell proliferation was obtained around the kidney specimens as previously described [27]. Kidney epithelial tubular and neoplastic cells were isolated with EpCAM (CD326) Ab-conjugated magnetic microbeads (Miltenyi Biotec, Bergisch Gladbach, Germany) under the effect of a magnetic field generated by the Mini MACS Separation Unit (Miltenyi Biotec), as previously described^5^. These cells were then characterized for EpCAM, CA IX, and NDUFA4L2 by immunocytochemistry. Primary ccRCC cells were used for proliferation studies at the second passage.

### Human cell lines

Caki-2 was cultured in McCoy's5A medium supplemented with 10% FBS and 1% penicillin-streptomycin-L-glutamine. Normoxic Caki-2 cells (21% O2) were maintained at 37°C in 5% CO_2_/95% air incubator. For hypoxic exposure, Caki-2 cell culture dishes were placed in a HERAcell 150i humidified hypoxia workstation (Thermo Scientific, Langenselbold, Germany) at 37°C in 1% O2 for 18h.

### Small interfering (siRNA) transfection

Isolated normal and tumor renal cells were cultured at 2x10^5^ cells per well in a 12-well plate with Keratinocyte Serum-Free Medium (KSFM), supplemented with 5 ng/ml recombinant epidermal growth factor (rEGF), 50 µg/ml bovine pituitary extract (BPE) (Gibco, Life Technologies, Monza, Italy ) and 30 ng/ml cholera toxin (Sigma-Aldrich, Milan, Italy). The same procedure was used for Caki-2 cells in McCoy's5A medium supplemented with 10% FBS. The transfection of siRNA was carried out using Lipofectamine 3000 (Life Technologies, Monza, Italy) in accordance with the manufacturer’s procedure. For each transfection, 50 nM of small interfering RNA targeting NDUFA4L2 (siNDUFA4L2) (Qiagen, Hilden, Germania) were used. In transfection experiments, a mock-transfection control was performed by putting cells through the transfection procedure without adding siRNA. The validated non-silencing siRNA sequence AllStars Negative Control siRNA (50 nM, Qiagen, Hilden, Germania) was used as negative control. Each transfection experiment was performed in triplicate. After transfection, normal and tumor renal cells were incubated for 24h and 72h at 37°C in 5% CO2 and used for total RNA extraction, immunofluorescence, wound healing and cell viability assays, respectively.

Caki-2 cells were transfected for 72h at 37°C in 5% CO2 in normoxic conditions. For hypoxia conditions, after 54h from the addition or not of the siRNA, Caki-2 cells were transferred and maintained at 37°C in 1% O2 for 18h. Caki-2 cells were used for western blot, cell proliferation and ROS measurement assays.

### Gene expression profile of primary renal tumor cells

Transcriptome data were generated using the HumanHT-12 v3 Expression BeadChip (Release 38, Illumina, San Diego, CA. USA). In this process, 500 ng total RNA were used to synthesize biotin-labeled cRNA using the Illumina®TotalPrep™ RNA amplification kit (Applied biosystems/Ambion, USA). Quality of labelled cRNA was measured using the NanoDrop® ND-100 spectrophotometer and the Agilent 2100 Bioanalyzer. 750 ng biotinylated cRNA was used for hybridization to gene-specific probes on the Illumina microarrays. The Illumina arrays were then scanned with the HiScanSQ.

Microarray statistical analyses were performed with Genespring GX 11.0 software (Agilent Tech Inc., Santa Clara, CA, USA). Identification of genes differentially expressed between primary normal (n=5) and neoplastic renal cells (n=6) was carried out with the Benjamini-Hochberg FDR method and gene probe sets were filtered on the basis of the false discovery rate, FDR, (adjusted-P value with multiple testing on 1000 permutations) and fold-change. The fold-change filter was set at 1.5-fold in each comparison. Only genes that were significantly (adjusted-P value <0.05 and fold-change ≥1.5) modulated were considered for further analysis. The raw data have been deposited in the Gene Expression Omnibus database (GEO accession number GSE117890). Microarray data are MIAME compliant.

### Real Time PCR

Total RNA of normal and tumor tissues were reverse transcribed with the High-Capacity cDNA Reverse Transcription Kit (Applied Biosystems Foster City, CA, USA), following the manufacturer’s instructions. Quantitative real-time polymerase chain reactions (PCR) were performed using the iQTM SYBR Green Supermix buffer (6mMMgCl2, dNTPs, iTaq DNA polymerase, SYBR Green I, fluorescein and stabilizers) (BIO-RAD Laboratories, Hercules, CA, USA). The primers used for Real Time PCR are listed in Supplementary Table 7.

Quantification of the mRNA levels was performed on a MiniOpticon Real-Time PCR detection system (BIO-RAD Laboratories). In the PCR reactions, the following protocol was used: polymerase activation at 95◦C for 3 min, followed by 45 cycles at 95◦C for 10 s, 60◦C for 30 s. Melting curves were generated through 60 additional cycles (65◦C for 5 s with an increment of 0.5◦C/cycle). Gene expression results were obtained as mean Ct (threshold cycle) values of triplicate samples. Expression was determined using the 2^-ΔΔCt^ method. Expression values were normalized to β-Actin.

### Data mining using the Oncomine gene expression microarray datasets and Metabologram

NDUFA4L2 gene expression was analyzed using the microarray gene expression datasets deposited in the Oncomine database (https://www.oncomine.org/resource/login.html).

Firstly, to address the differential expression of genes between renal cancer and normal tissues, combined filters were applied to display the corresponding datasets. Cancer Type was defined as Clear Cell Renal Cell Carcinoma; Data Type was mRNA; Analysis Type was Cancer vs Normal Analysis. The expression values of the genes (log2 median-centered intensity) were read from the displayed bar chart. Student’s t test was used to calculate the significance.

In addition, the oxidative phosphorylation pathway was explored using the Metabologram data portal (http://sanderlab.org/kidneyMetabProject), a web-based application that integrates transcriptomic and metabolomics data using both gene expression (derived from TCGA database) and metabolite abundance data (derived from MSKCC metabolomics dataset).

### Immunohistochemistry

Immunohistochemical evaluation of NDUFA4L2 and GLUT1 proteins expression was done on paraffin-embedded tissue sections derived from 20 normal kidney samples and 390 tumoral samples. Sections (3 mm) of paraffin-embedded tissue were deparaffinized and rehydrated through xylenes and graded alcohol series. Slides were subjected to specific epitope demasking by microwave treatment at 700 W in citrate buffer (0.01 M, pH 6.0). After antigen retrieval, the tissue samples were incubated for 10 minutes with 3% H2O2 to block endogenous peroxidase activity. Sections were blocked with Protein Block Serum-Free (Dako) at room temperature for 10 minutes and then incubated with anti-NDUFA4L2 antibody (1:100 dilution, Proteintech, Chicago, IL, USA) and anti-GLUT1 antibody (1:200, Novus Biologicals, Littleton, CO, USA) at 4C overnight. Binding of the secondary biotinylated antibody was detected using the Dako Real EnVision Detection System, Peroxidase/DAB kit (Dako, Agilent, Santa Clara, US), according to the manufacturer’s instructions. Sections were counterstained with Mayer's haematoxylin (blue) and mounted with glycerol (Dako Cytomation). Negative controls were obtained by incubating serial sections with the blocking solution and then omitting the primary antibodies. Digital images were obtained using the Aperio ScanScope CS2 device (Aperio Technologies, Vista, CA) and further analyses of the scanned images were performed with the ImageScope V12.1.0.5029 (Aperio). Specific staining was quantified by applying the Positive Pixel Count v9_v10.0.0.1805 algorithm (Aperio) and expressed as percentage of positive pixels in the analyzed area.

### Immunofluorescence microscopy

Tumor renal cells (ccRCC cells) were seeded at a density of 2x10^5^ on glass coverslips and left to adhere overnight at 37°C in 5% CO2. The cells were exposed to 50nM of siNDUFA4L2 or incubated in medium without siRNA for 72h. These preparations were double-stained for NDUFA4L2 (Proteintech, Chicago, IL, USA) and MitoTracker™ Red CMXRos (Molecular Probes, Life Technologies, Monza, Italy ); OGG1 (Novus Biologicals, Littleton, CO, USA) and 8-Hydroxyguanosine (Abcam, Cambridge, UK); LC3 (Novus Biologicals Littleton, CO, USA) and MitoTracker™ Red CMXRos; Gamma-H2AX (Abcam, Cambridge, UK) and MitoSOX red (Molecular Probes, Life Technologies, Monza, Italy ). The expression and localization of proteins was evaluated by indirect immunofuorescence and confocal microscopy analysis. Mitocondrial mass was determined by incubating the live cells with 500 nM MitoTracker™ Red CMXRos in growth medium, for 20 min at 37°C, whereas to study mitochondrial superoxide generation, live-cells were stained with 5 μM MitoSOX red for 10 min at 37°C. The cells were fixed using ice-cold 4% paraformaldehyde for 10 min at room temperature. Then the preparations were blocked with 1% BSA in PBS for 1 h at room temperature and incubated overnight at 4 °C with a primary antibody against NDUFA4L2 (1:25 in blocking) or LC3 (1:100 in blocking) or Gamma-H2AX (1:100 in blocking), followed by incubation for 1h at 37°C with the secondary antibody goat anti-rabbit IgG FITC (1:200; Novus Biologicals). To study markers for measuring the rate of oxidative damage to nucleic acids and the proteins involved in oxidative stress-induced DNA demethylation, the preparations were incubated overnight at 4 °C with a primary antibody against 8-Hydroxyguanosine (1:100 in blocking), followed by incubation for 2h with the secondary antibody Alexa Fluor 555 goat anti-mouse (1:200; Molecular Probes). Then, they were washed in PBS and incubated overnight at 4 °C with primary antibodies against OGG1 (1:100 in blocking) followed by incubation for 1 h at 37°C with the secondary antibody goat anti-rabbit IgG FITC (Novus Biologicals Littleton, CO, USA). All preparations were counterstained with TO-PRO-3 (Molecular Probes). Negative controls were performed by omitting the primary antibodies. Specific fluorescence was acquired by a Leica TCS SP2 (Leica, Wetzlar, Germany) confocal laser-scanning microscope using an ×63 objective lens.

### Mitochondrial membrane potential

Mitochondrial membrane potential was determined by incubating the cells with the fluorescent dye tetramethylrhodamine ethyl ester (TMRE; Sigma-Aldrich, Milan, Italy). Cells were incubated with 2.5 µM TMRE for 1h at 37°C and subsequently analyzed by immunofluorescence.

### Western blot

Normoxic Caki-2 cells and hypoxic Caki-2 cells both with and without siRNA NDUFA4L2 were homogenized in 1X lysis buffer (50 mM Tris-HCl, pH 7.4, 5 mM EDTA, 250 mM NaCl, 0.1% Triton X-100 [Sigma-Aldrich, Milan, Italy, T8787]) supplemented with protease and phosphatase inhibitors (Complete mini [Roche, 11836153001] and phosSTOP [Roche, 04906845001]). 50 µg of protein extracts from each sample were denatured in 4X Laemmli sample buffer [Biorad, #1610747] and loaded into an SDS-polyacrylamide gel for western blot analysis. Western blots were performed using anti b-Actin [Cell Signaling Technology, #3700S], anti-LC3 (Cell Signaling Technology, #12741), and anti-TOM20 (Santa Cruz Biotechnogy, sc-17764). Western blots were developed with the Clarity TM Western ECL substrate chemiluminescence reagent (Biorad, Uppsala, Sweden #1705061) as per manufacturer’s instructions. The densitometric evaluation was performed by the ImageJ software.

### Wound healing assay

2×10^5^ normal and tumor renal cells were seeded onto a six-well plate with 2 mL of Keratinocyte Serum-Free Medium (KSFM) to create a confluent monolayer, supplemented with 5ng/ml recombinant epidermal growth factor (rEGF), 50μg/ml bovine pituitary extract (BPE) (Gibco) and 30ng/ml cholera toxin (Sigma-Aldrich, Milan, Italy). Cells were incubated overnight at 37°C, 5% CO2, and then exposed to 50nM of siRNA NDUFA4L2 (Quiagen) or incubated in medium without siRNA for 72h. After 24h from the treatment, a wound was manually created by scraping the cell monolayer with a P200 pipette tip. A reference mark was created on the dish and a time 0 image was acquired. After 24 and 48 hours, additional images were taken in the matched region, and the wound-healing area was quantified with ImageJ software (http://rsbweb.nih.gov/ij/). Each experimental condition was performed in triplicate.

### Cell viability assay

Cell viability after exposure to 50nM of siNDUFA4L2 or to siNDUFA4L2 and 10μM cis-Diamminedichloroplatinum(II) (cisplatin) was evaluated using the trypan blue dye exclusion and 3-(4,5-dimethylthiazol-2-yl)-2,5-diphenyltetrazolium bromide (MTT) assay. For the dye exclusion test and MTT assay, normal and tumor cells were seeded at a density of 1.5x10^5^ and 1.5x10^4^ cells in six-well and 96-well plates, respectively (Sigma Aldrich, Milan, Italy) and incubated overnight at 37°C, 5% CO2 . In the first part of the experiment, the cells were exposed to 50nM of siNDUFA4L2 for 72h or incubated in medium alone. In the second part of the experiment, 24h after the addition of siRNA, the cells were treated with cisplatin 10 μM for 1h and 2h. After several washes to remove cisplatin the cells were again incubated in medium with 50nM of siRNA NDUFA4L2 for 48h. Each experimental condition was performed in triplicate.

### Cell proliferation and ROS measurement assay

For the cell proliferation assay, 2 × 10^5^ Caki-2 cells were plated in a 10 cm dish, 1 day before exposure to siRNA NDUFA4L2 or to medium alone, and cultured in normoxic conditions. The cells were exposed to 50nM of siNDUFA4L2 for 24h and then treated with 100 μM of ascorbic acid 2-phosphate (AA2P) for 24h. After several washes to remove AA2P, the cells were incubated for 18 h in normoxic or hypoxic conditions. Lastly, the cells were trypsinized and the viable cells were counted using trypan blue.

To study mitochondrial superoxide generation, Caki-2 cells were seeded at a density of 2x10^5^ on glass coverslips and left to adhere overnight at 37°C in 5% CO2. The next day the cells were washed and incubated with siNDUFA4L2 and AA2P, as described above. Caki-2 cells were stained with 5 μM MitoSOX red for 10 min at 37°C and washed three times before imaging. Caki-2 cells were fixed using ice-cold 4% paraformaldehyde for 10 min at room temperature. Then, nuclei were revealed by counterstaining with TO-PRO-3 (Molecular Probes). Images were taken with a Leica TCS SP2 (Leica, Wetzlar, Germany) confocal laser-scanning microscope using an X63 objective lens. Each experimental condition was performed in triplicate.

### In vivo chorioallantoic membrane (CAM) angiogenic assay

Fertilized White Leghorn chicken eggs were incubated at 37°C at constant humidity. On day 3 of incubation a square window was opened in the egg shell after removal of 2–3 ml of albumen so as to detach the developing CAM from the shell. The window was sealed with a glass and the eggs were returned to the incubator. Gelatin sponges (Gelfoam, Upjohn Company, Kalamazoo, U.S.A.) were cut to a size of 1 mm3 and placed on top of the growing CAM at day 8 incubation under sterile conditions according to Ribatti et al. [28]. The sponges were then adsorbed with 2 μl of cell suspension of tumor cells or tumor cells incubated with siNDUFA4L2. The angiogenic response was evaluated on day 12 of incubation after the implants by means of a stereomicroscope connected to an image analyzer system (Olympus Italia, Italy). Blood vessels entering the sponges within the focal plane of the CAM were counted by two observers in double blind fashion at a magnification of 50x. Means ± standard deviation (SD) were evaluated and the statistical significance of the differences between counts was determined by Student’s t-test for unpaired data.

### Quantification of mitochondrial DNA content

Mitochondrial DNA was isolated from normal, ccRCC and siNDUFA4L2 tumor cells using the Mitochondrial DNA Isolation Kit (Abcam, Cambridge, UK) according to the manufacturer’s instructions. Mitochondrial DNA content was measured using real-time quantitative PCR (qPCR) as previously described. In brief, the relative quantification of mitochondrial DNA content for each sample was determined using a set of mitochondrial specific primers: mt-Mito: forward 5’- CACTTTCCACACAGACATCA - 3’ , reverse 5’- TGGTTAGGCTGGTGTTAGGG – 3’ ; and set of nuclear- specific primers: B2M forward 5’- TGTTCCTGCTGGGTAGCTCT-3’ and reverse 5’- CCTCCATGATGCTGCTTACA-3’ . qPCR was performed with a MiniOpticon real-time PCR detection system (BIO-RAD Laboratories). In the PCR reactions, the following protocol was used: polymerase activation at 95°C for 3 minutes, followed by 45 cycles at 95°C for 10 seconds, 60°C for 30 seconds. Melting curves were generated through 60 additional cycles (65°C for 5 seconds with an increment of 0.5°C/cycle). Each experimental condition was performed in triplicate. The relative mitochondrial DNA content was calculated using the 2^-∆∆Ct^ method.

### ATP assay

Normal cells and ccRCC cells were seeded at a density of 1.5x10^4^ cells in 96-well plates with 200 μL of KSFM, supplemented with 5ng/ml rEGF, 50μg/ml BPE and 30ng/ml cholera toxin, incubated overnight at 37°C, 5% CO2 and then exposed to 50nM of siRNA NDUFA4L2 or incubated in medium without siRNA for 72h. CellTiter-Glo® assay reagent (Promega, Madison, Wisconsin, US) was added in according to the manufacturer’s instructions and the luminescent signal, proportional to the amount of ATP present, was measured using a GloMx-Multi+Microplate Multimode Reader (Promega, Madison, Wisconsin, US). Each experimental condition was performed in triplicate.

### Complex I and IV activity

The activities of Complex I and Complex IV were measured in ccRCC cells and siNDUFA4L2 ccRCC cells using the Complex I Enzyme Activity Microplate Assay Kit and Complex IV Human Enzyme Activity Microplate Assay Kit (Abcam, Cambridge, UK), according to the manufacturer’s instructions. Each experimental condition was performed in triplicate.

### Availability of data and material

The datasets generated and/or analysed during the current study are available in the GEO repository:

Accession number GSE47032

Accession number GSE117890

## Supplementary Material

Supplementary Figures

Supplementary Tables 1, 3-7

Supplementary Table 2
