# Supplementary material for: Integrated multi-omics characterization reveals a distinctive metabolic signature and the role of NDUFA4L2 in promoting angiogenesis, chemoresistance, and mitochondrial dysfunction in clear cell renal cell carcinoma
Source: Aging (Albany NY). 2018 Dec 11;10(12):3957–85. doi: 10.18632/aging.101685 (PMC6326659; doi:10.18632/aging.101685)
Supplement: Supplementary Figures [file aging-10-101685-s001.pdf]

## SUPPLEMENTARY FIGURES

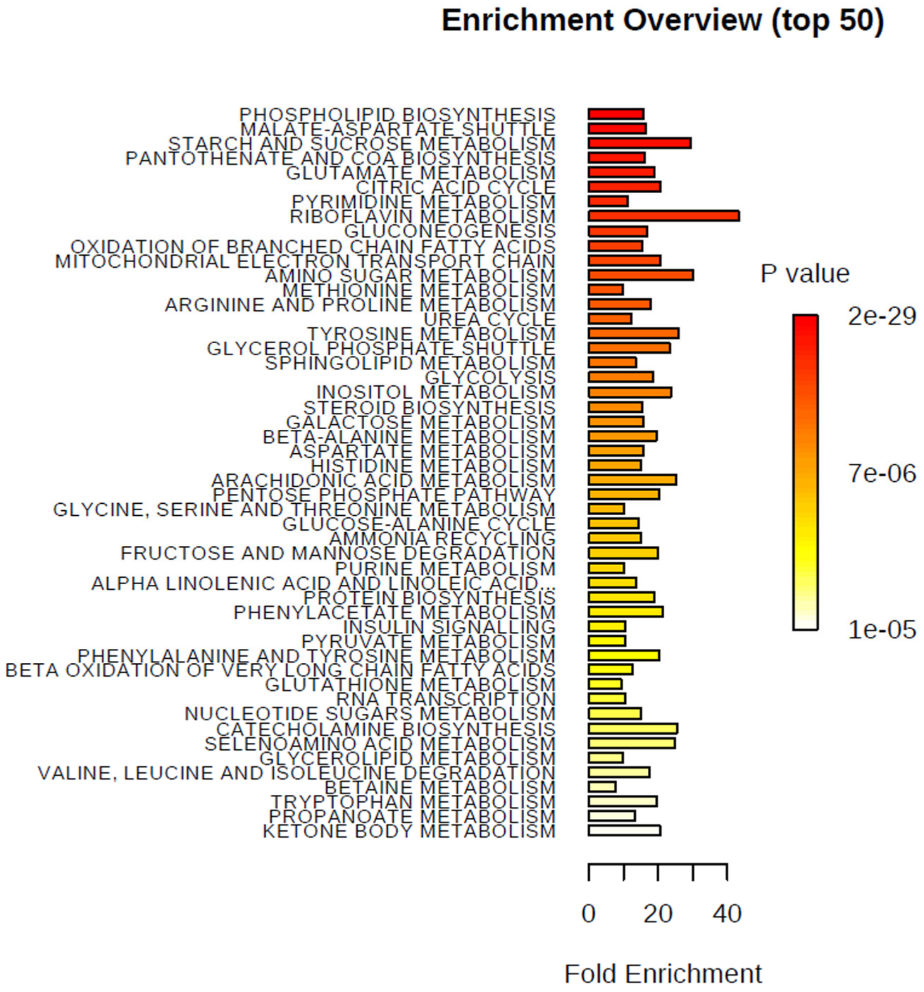


**Supplementary Figure 1.** Metabolite set enrichment overview performed in MetaboAnalyst 3.0

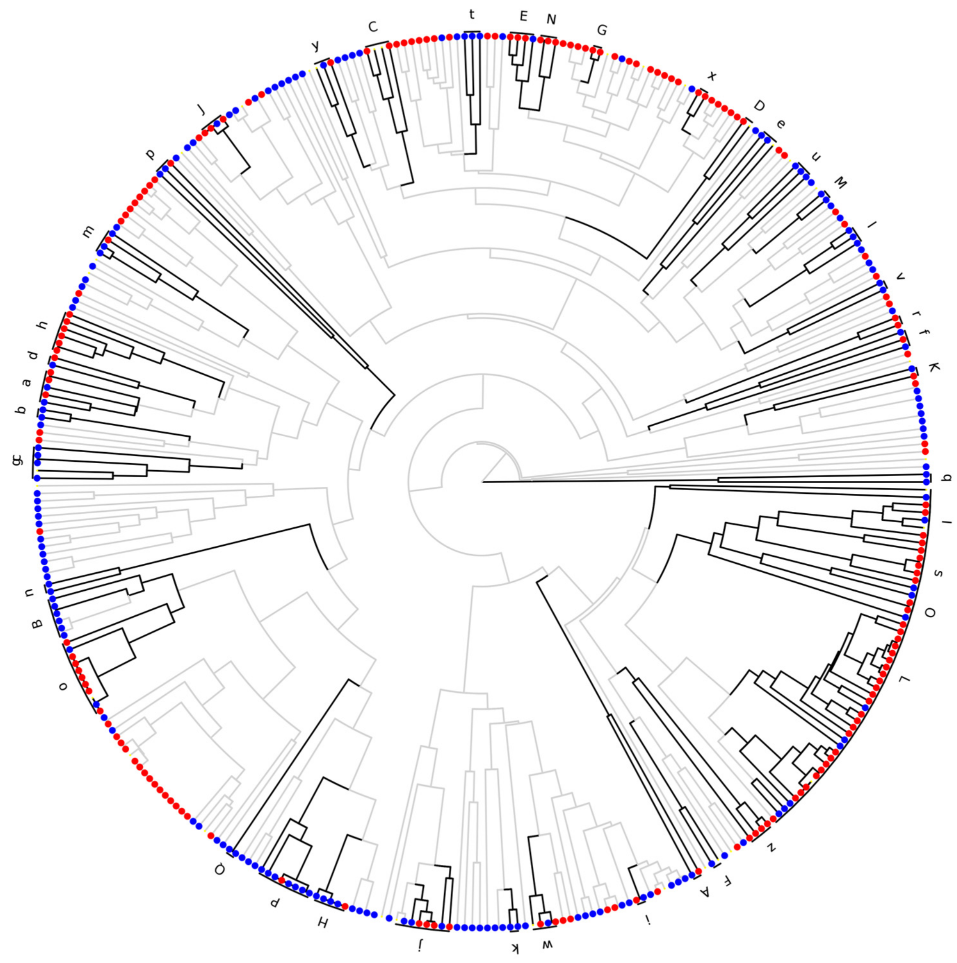

**Supplementary Figure 2.** Tanimoto chemical similarity mapping of all identified metabolites in ccRCC. Clusters are defined by comparing within- versus between group similarities, forming a clustered chemical similarity tree. Dark black lines indicate boundaries of clusters significantly different in tumor versus normal tissue ($p<0.05$). Cluster letter labels are detailed in Supplementary Table 2. Increased metabolites in ccRCC are labeled as red nodes. Decreased metabolites are blue nodes.

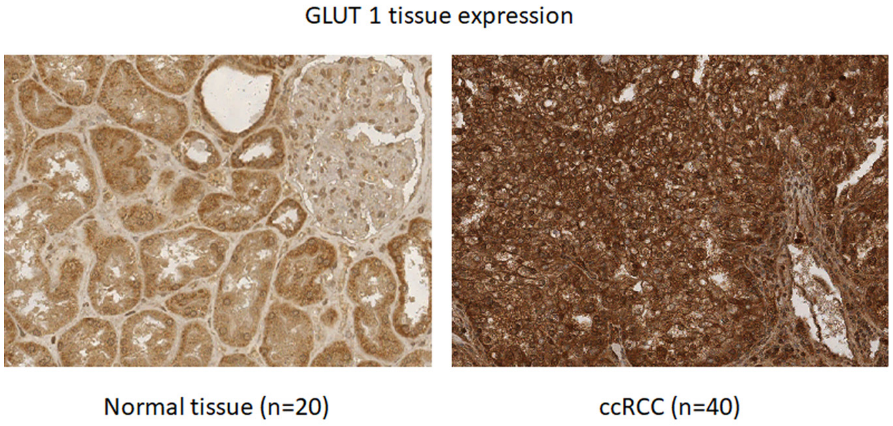


**Supplementary Figure 3.** Glut1 expression in normal (n=20) and ccRCC (n=40) specimens.

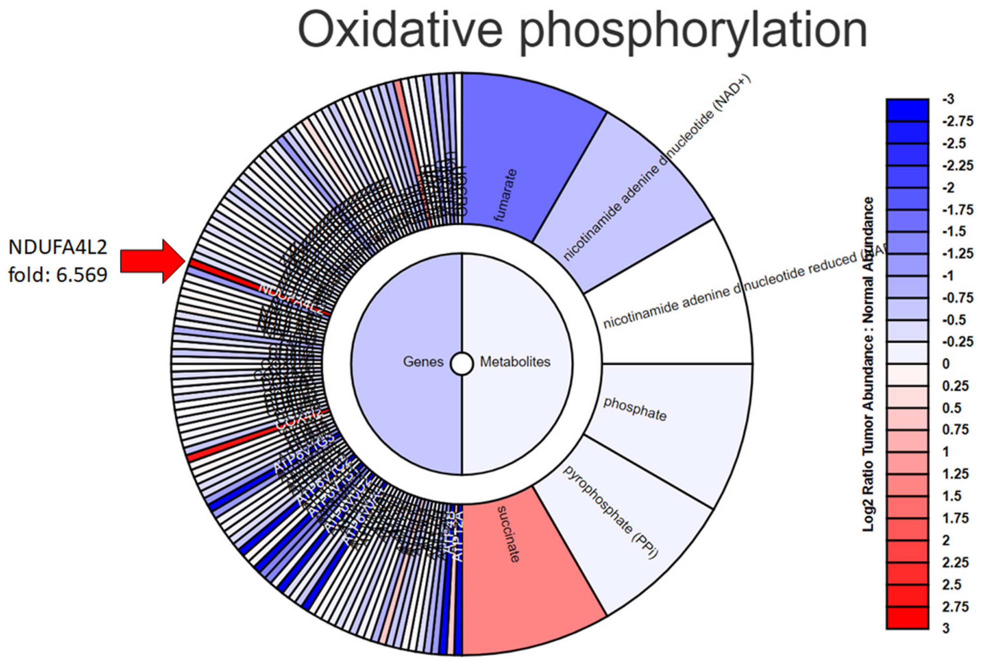


**Supplementary Figure 4.** Exploration of the "Metabologram" Data Portal. The changes in the "Oxidative phosphorylation" metabolism pathway are shown in both transcripts and metabolites when comparing tumors to adjacent normal kidney tissues.

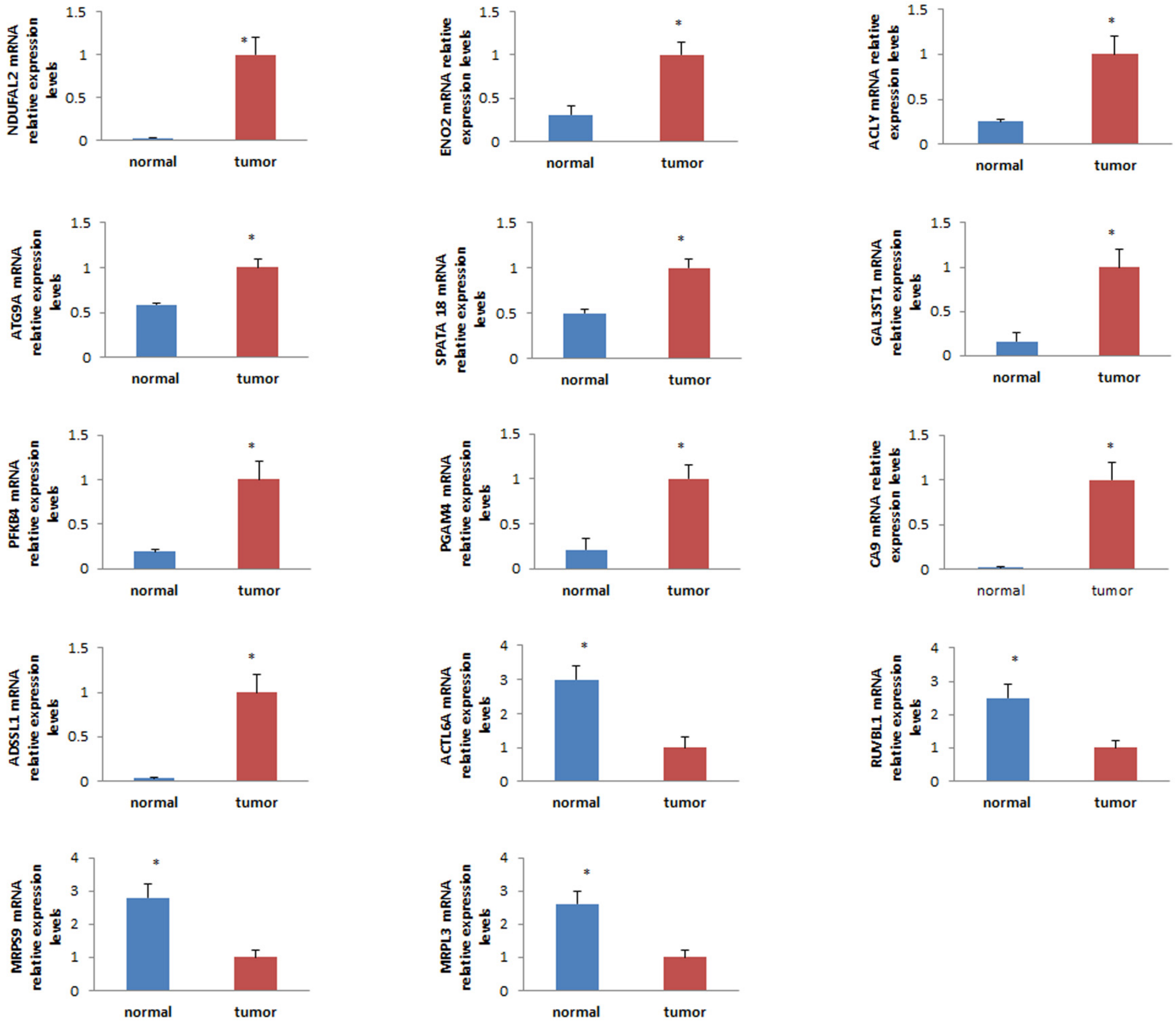


**Supplementary Figure 5.** Gene expression by real-time PCR.

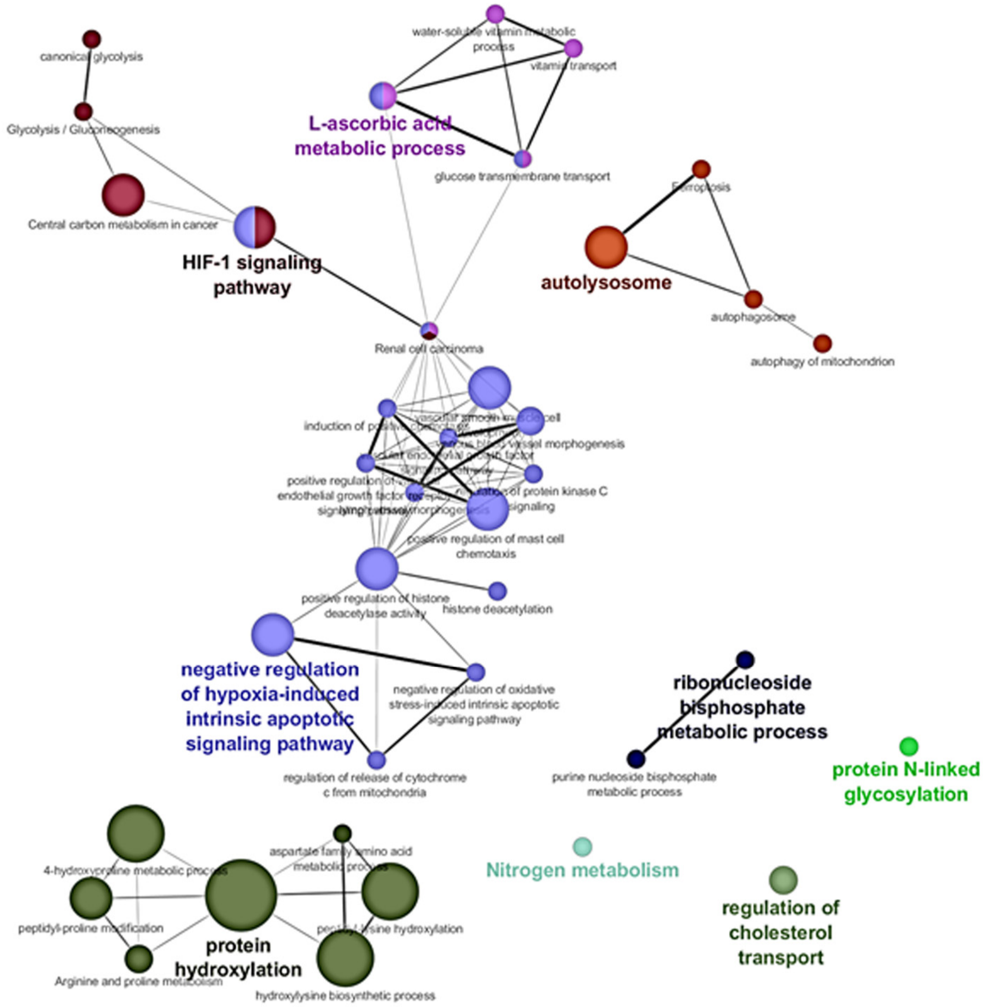


**Supplementary Figure 6.** Enriched GO network groups using ClueGO for GSE117890 dataset analysis. Biological processes (GO category) are visualized (kappa score ≥ 0.3) as a functional grouped network and only the most significant interactions are shown. Each node represents a biological process. Edges represent connections between the nodes and the length of each edge reflects the relatedness of two processes.
