# Supplementary material for: Integrated multi-omics characterization reveals a distinctive metabolic signature and the role of NDUFA4L2 in promoting angiogenesis, chemoresistance, and mitochondrial dysfunction in clear cell renal cell carcinoma
Source: Aging (Albany NY). 2018 Dec 11;10(12):3957–85. doi: 10.18632/aging.101685 (PMC6326659; doi:10.18632/aging.101685)
Supplement: Supplementary Tables 1, 3-7 [file aging-10-101685-s002.pdf]

SUPPLEMENTARY TABLES

Supplementary Table 1. Clinical and pathological characteristics of tissues collected from patients who underwent radical or partial nephrectomy for ccRCC. These specimens were used for metabolomics analysis and to obtain primary cell cultures.

**Variable**	**n=40**
Age (years)<br>median<br>range	 52<br>24-81
Gender<br>Male<br>Female	 26 (65%)<br>14 (35%)
Dimensions (cm)<br>median<br>range	 5.5<br>2 -12
Pathological stage<br>pT1a<br>pT1b<br>pT2<br>pT3	 18 (45%)<br>12 (30%)<br>5 (12.2%)<br>5 (12.5%)
pN+	2 (5%)
cM+	2 (5%)
Fuhrman grade<br>G1-2<br>G3-4	 20 (50%)<br>20 (50%)

Supplementary Table 2. ChemRich results. Please browse Full text version to see the data

Supplementary Table 3. NDUFA4L2 expression in ccRCC. Data from 5 available datasets (Source: Oncomine, Compendia Bioscience, Ann Arbor, MI).

Dataset	Sample set	Measured genes	ccRCC samples examined	Normal samples examined	Fold change	p-value
**Gumz**	ccRCC vs Normal	12,624	10	10	53.935	2.60E-11
**Beroukhim**	Non-hereditary ccRCC vs Normal	12,624	27	11	50.115	2.37E-14
**Beroukhim**	Hereditary ccRCC vs Normal	12,624	32	11	75.690	5.55E-23
**Jones**	ccRCC vs Normal	12,624	23	23	8.361	7.32E-8
**Lenburg**	ccRCC vs Normal	17,779	9	9	23.340	5.51E-9
**Yusenko**	ccRCC vs Normal	17,779	26	3	86.565	7.04E-15

Supplementary Table 4. Clinical and pathological characteristics.

Variable	n=390
**Age (years)**	
**median**	60
**95% CI**	55-62
**Gender**	
**Male**	258 (66%)
**Female**	132 (34%)
**Dimensions (cm)**	
**median**	5.0
**95% CI**	4.5 - 5.5
**Pathological stage**	
**pT1a**	184 (47%)
**pT1b**	100 (26%)
**pT2**	46 (12%)
**pT3**	42 (11%)
**pT4**	18 (4%)
**pN+**	53 (13.6%)
**cM+**	44 (11.3%)
**Fuhrman grade**	
**G1-2**	246 (63%)
**G3-4**	144 (37%)
**Follow-up**	
**Median (months)**	45
**95% CI**	39 – 44

Supplementary Table 5. Description of Metabolon QC Samples.

Type	Description	Purpose
MTRX	Large pool of human plasma maintained by Metabolon that has been characterized extensively.	Assure that all aspects of Metabolon process are operating within specifications.
CMTRX	Pool created by taking a small aliquot from every customer sample.	Assess the effect of a non-plasma matrix on the Metabolon process and distinguish biological variability from process variability.
PRCS	Aliquot of ultra-pure water	Process Blank used to assess the contribution to compound signals from the process.
SOLV	Aliquot of solvents used in extraction.	Solvent blank used to segregate contamination sources in the extraction.

Supplementary Table 6. Metabolon QC Standards.

Type	Description	Purpose
DS	Derivatization Standard	Assess variability of derivatization for GC/MS samples.
IS	Internal Standard	Assess variability and performance of instrument.
RS	Recovery Standard	Assess variability and verify performance of extraction and instrumentation.

Supplementary Table 7. Primers used for Real Time PCR.

PGAM4	5'-GAGCCCGACCATCCTTTCTA-3'<br>5'-CAGTACACGTTTCCCCTCCT-3'
ENO2	5'-GAACAGTGAAGCCTTGGAGC-3'<br>5'-AAAGTCCTGGTAGAGTGCCC-3'
ACLY	5'-AAGTGGGGTGACATCGAGTT-3'<br>5'-CTTTGGGGTTCAGCAAGGTC-3'
GAL3ST1	5'-TGTTCGAGTCCTCCTTCCAC-3'<br>5'-TTTCGGAGGTAGTGGGCATT-3'
ADDSSL1	5'-CGTGTATGGCGTGGTGAAAG-3'<br>5'-AATCCGTTGACCATGTGAGC-3'
PFKFB4	5'-TGAGGTCATAGCTGCCAACA-3'<br>5'-TGGCCCACATCCATGATCTT-3'
CA9	5'-GCCGCTACTTCCAATATGAGG-3'<br>5'-CCTCAATCACTCGCCCATTC-3'
ACTL6A	5'-GCCAGTCTCCATCCTGTTCT-3'<br>5'-CAGTGGTATGAGTGGCTCCA-3'
RUVBL1	5'-ATCGCTCCCATCGTCATCTT-3'<br>5'-TCATCACTCGGTCCAGAAGG-3'
NDUFA4L2	5'-AGATCAAAAGACATCCGGGGA-3'<br>5'-CGAGTCGCAGCAAGTAAAGC-3'
ATG9A	5'-ATGTCTGCCCTTCCGTATTG-3'<br>5'-GCGGTGGTAGATGTCCAGTT-3'
SPATA 18	5'-GAAGCTGTCACCAGGAGAGG-3'<br>5'-AGGACTCCGACTTCGAGACA-3'
MRPS9	5'-CAAGGAATGGCCTTTAGCAA-3'<br>5'-GTCCTGTGTGATCGGGAAGT-3'
MRPL3	5'-CCAAGGATGGTCAAAAGCAT-3'<br>5'-CAGACAGGGTTGCCATTTTT-3'
B-ACTIN	5'-AATCTGGCACCACACCTTCT-3'<br>5'-AGCCTGGATAGCAACGTACA-3'
