# Supplementary material for: Integrated multi-omics characterization reveals a distinctive metabolic signature and the role of NDUFA4L2 in promoting angiogenesis, chemoresistance, and mitochondrial dysfunction in clear cell renal cell carcinoma
Source: Aging (Albany NY). 2018 Dec 11;10(12):3957–85. doi: 10.18632/aging.101685 (PMC6326659; doi:10.18632/aging.101685)
Supplement: Supplementary Table 2 [file aging-10-101685-s003.doc]

| **Cluster name** | **Cluster size** | **p-values** | **FDR** | **Key compound** | **Altered metabolites** | **Increased** | **Decreased** | **Increased ratio** | **Altered Ratio** |
| --- | --- | --- | --- | --- | --- | --- | --- | --- | --- |
| Dipeptides | 78 | 1.8E-57 | 8.1E-56 | N-acetylaspartate (NAA) | 71 | 58 | 13 | 0.7 | 0.9 |
| UnSaturated FA | 20 | 5.6E-15 | 1.2E-13 | erucate (22:1n9) | 18 | 15 | 3 | 0.8 | 0.9 |
| Carnitine | 15 | 1.3E-11 | 1.8E-10 | octanoylcarnitine | 13 | 6 | 7 | 0.4 | 0.9 |
| Glutamates | 5 | 2.8E-10 | 3.1E-09 | N-acetyl-aspartyl-glutamate (NAAG) | 5 | 2 | 3 | 0.4 | 1 |
| Hydroxycholesterols | 3 | 0.000000001 | 8.8E-09 | 7-beta-hydroxycholesterol | 3 | 0 | 3 | 0 | 1 |
| Carboxylic Acids | 3 | 1.7E-09 | 0.000000013 | phosphoenolpyruvate (PEP) | 3 | 0 | 3 | 0 | 1 |
| Oligopeptides | 11 | 0.00000001 | 0.000000063 | aspartylleucine | 9 | 9 | 0 | 0.8 | 0.8 |
| Saturated FA | 11 | 0.000000012 | 0.000000063 | stearate (18:0) | 10 | 8 | 2 | 0.7 | 0.9 |
| Purine Nucleosides | 9 | 0.00000012 | 0.00000053 | adenosine | 8 | 1 | 7 | 0.1 | 0.9 |
| Amino Acids | 8 | 0.00000013 | 0.00000053 | citrulline | 8 | 3 | 5 | 0.4 | 1 |
| Unsaturated_Lysophosphatidylcholines | 8 | 0.00000013 | 0.00000053 | 1-linoleoylglycerophosphocholine | 8 | 1 | 7 | 0.1 | 1 |
| Saturated_Glycerides | 3 | 0.00000073 | 0.0000027 | 1-stearoylglycerol (1-monostearin) | 3 | 0 | 3 | 0 | 1 |
| Xanthines | 3 | 0.0000008 | 0.0000027 | xanthine | 3 | 0 | 3 | 0 | 1 |
| Hexosephosphates | 7 | 0.0000012 | 0.0000039 | mannose-6-phosphate | 7 | 7 | 0 | 1 | 1 |
| Neutral Glycosphingolipids | 3 | 0.0000032 | 0.0000093 | palmitoyl sphingomyelin | 3 | 2 | 1 | 0.7 | 1 |
| Adenine Nucleotides | 3 | 0.0000057 | 0.000015 | nicotinamide adenine dinucleotide (NAD+) | 3 | 1 | 2 | 0.3 | 1 |
| Peptides | 5 | 0.0000059 | 0.000015 | N-acetylmethionine | 4 | 4 | 0 | 0.8 | 0.8 |
| Cytosine Nucleotides | 3 | 0.000016 | 0.000039 | cytidine 5'-monophosphate (5'-CMP) | 3 | 2 | 1 | 0.7 | 1 |
| Oligosaccharides | 5 | 0.000045 | 0.0001 | maltose | 5 | 5 | 0 | 1 | 1 |
| Dicarboxylic Acids | 5 | 0.000046 | 0.0001 | malate | 5 | 2 | 3 | 0.4 | 1 |
| Amino Acids, Sulfur | 4 | 0.000048 | 0.0001 | methionine | 3 | 0 | 3 | 0 | 0.8 |
| Saturated_Lysophosphatidylcholines | 5 | 0.000071 | 0.00014 | 1-palmitoylglycerophosphocholine | 5 | 1 | 4 | 0.2 | 1 |
| Amino Acids, Aromatic | 5 | 0.00015 | 0.00029 | tyrosine | 4 | 1 | 3 | 0.2 | 0.8 |
| Sugar Alcohols | 5 | 0.00018 | 0.00033 | arabitol | 3 | 0 | 3 | 0 | 0.6 |
| Phenols | 5 | 0.00028 | 0.00049 | 3-(4-hydroxyphenyl)lactate | 4 | 1 | 3 | 0.2 | 0.8 |
| Glutarates | 4 | 0.00034 | 0.00049 | 2-hydroxyglutarate | 4 | 1 | 3 | 0.2 | 1 |
| Pyridines | 4 | 0.00034 | 0.00049 | nicotinamide | 4 | 2 | 2 | 0.5 | 1 |
| Amino Acids, Basic | 4 | 0.00034 | 0.00049 | glutamine | 4 | 1 | 3 | 0.2 | 1 |
| Saturated_Phosphatidylethanolamines | 4 | 0.00034 | 0.00049 | 2-palmitoylglycerophosphoethanolamine* | 4 | 0 | 4 | 0 | 1 |
| Bile Pigments | 4 | 0.00035 | 0.00049 | bilirubin (Z,Z) | 4 | 4 | 0 | 1 | 1 |
| Glyceric Acids | 4 | 0.00035 | 0.00049 | 2-phosphoglycerate | 4 | 0 | 4 | 0 | 1 |
| Amino Acids, Cyclic | 4 | 0.00038 | 0.00052 | histidine | 4 | 1 | 3 | 0.2 | 1 |
| Hexoses | 4 | 0.0004 | 0.00053 | fructose | 4 | 3 | 1 | 0.8 | 1 |
| Pyrimidines | 3 | 0.00042 | 0.00055 | 5,6-dihydrouracil | 2 | 1 | 1 | 0.3 | 0.7 |
| Pregnanes | 3 | 0.0009 | 0.0011 | cortisone | 2 | 0 | 2 | 0 | 0.7 |
| Pyrrolidinones | 3 | 0.0025 | 0.0026 | 5-oxoproline | 3 | 0 | 3 | 0 | 1 |
| Saturated_Lecithins | 3 | 0.0025 | 0.0026 | 2-eicosatrienoylglycerophosphocholine* | 3 | 1 | 2 | 0.3 | 1 |
| Saturated_Lysophospholipids | 3 | 0.0025 | 0.0026 | 1-stearoylglycerophosphoethanolamine | 3 | 0 | 3 | 0 | 1 |
| Tocopherols | 3 | 0.0025 | 0.0026 | alpha-tocopherol | 3 | 3 | 0 | 1 | 1 |
| Amino Acids, Branched-Chain | 3 | 0.0025 | 0.0026 | isoleucine | 3 | 0 | 3 | 0 | 1 |
| Inositol | 3 | 0.0025 | 0.0026 | myo-inositol | 3 | 0 | 3 | 0 | 1 |
| Hippurates | 3 | 0.0025 | 0.0026 | hippurate | 3 | 0 | 3 | 0 | 1 |
| Ethanolamines | 3 | 0.0026 | 0.0026 | ethanolamine | 3 | 0 | 3 | 0 | 1 |
| Phosphatidylinositols | 3 | 0.0026 | 0.0026 | 1-stearoylglycerophosphoinositol | 3 | 0 | 3 | 0 | 1 |

| **Compound.Name** | **InChiKeys** | **Pubchem.ID** | **SMILES** | **pvalue** | **foldchange** | **CID** | **Cluster**  **Number** | **xlogp** | **ClusterLabel** | **TreeLabels** | **FDR** |
| --- | --- | --- | --- | --- | --- | --- | --- | --- | --- | --- | --- |
| 2-hydroxyglutarate | HWXBTNAVRSUOJR-UHFFFAOYSA-N | 43 | C(CC(=O)O)C(C(=O)O)O | 0 | 3.3 | 43 | 10 | -1.116 | Glutarates | a | 0 |
| 3-methyl-2-oxovalerate | JVQYSWDUAOAHFM-UHFFFAOYSA-N | 47 | CCC(C)C(=O)C(=O)O | 0.0005 | 0.41 | 47 | 75 | 0.689 | Carboxylic Acids | b | 0.078 |
| 2-phosphoglycerate | GXIURPTVHJPJLF-UHFFFAOYSA-N | 59 | C(C(C(=O)O)OP(=O)(O)O)O | 0 | 0.37 | 59 | 68 | -2.952 | Glyceric Acids | c | 0 |
| 4-methyl-2-oxopentanoate | BKAJNAXTPSGJCU-UHFFFAOYSA-N | 70 | CC(C)CC(=O)C(=O)O | 0.0012 | 0.49 | 70 | 75 | 0.904 | Carboxylic Acids | b | 0.16 |
| 3-ureidopropionate | JSJWCHRYRHKBBW-UHFFFAOYSA-N | 111 | C(CNC(=O)N)C(=O)O | 0.07 | 1.5 | 111 | 59 | -1.628 |  |  | 1 |
| 5-methyltetrahydrofolate (5MeTHF) | ZNOVTXRBGFNYRX-UHFFFAOYSA-N | 146 | CN1C(CNC2=C1C(=O)N=C(N2)N)CNC3  =CC=C(C=C3)C(=O)NC(CCC(=O)O)C(=O)O | 0.0014 | 0.48 | 146 | 37 | -2.254 | Tetrahydrofolates |  | 0.18 |
| acetylcholine | OIPILFWXSMYKGL-UHFFFAOYSA-N | 187 | CC(=O)OCC[N+](C)(C)C | 0.074 | 2.3 | 187 | 62 | -0.097 | Biogenic Amines |  | 1 |
| allantoin | POJWUDADGALRAB-UHFFFAOYSA-N | 204 | C1(C(=O)NC(=O)N1)NC(=O)N | 0.057 | 0.61 | 204 | 95 | -1.169 | Hydantoins |  | 1 |
| beta-alanine | UCMIRNVEIXFBKS-UHFFFAOYSA-N | 239 | C(CN)C(=O)O | 0.0012 | 3 | 239 | 59 | -1.026 | Amino Acids |  | 0.16 |
| betaine | KWIUHFFTVRNATP-UHFFFAOYSA-N | 247 | C[N+](C)(C)CC(=O)[O-] | 0.0028 | 0.5 | 247 | 62 | -1.227 | Trimethyl Ammonium Compounds |  | 0.32 |
| citrate | KRKNYBCHXYNGOX-UHFFFAOYSA-N | 311 | C(C(=O)O)C(CC(=O)O)(C(=O)O)O | 0 | 13 | 311 | 91 | -2.247 | Citrates | d | 0 |
| hippurate | QIAFMBKCNZACKA-UHFFFAOYSA-N | 464 | C1=CC=C(C=C1)C(=O)NCC(=O)O | 0 | 0.09 | 464 | 2 | 0.154 | Hippurates |  | 0 |
| 2-aminoadipate | OYIFNHCXNCRBQI-UHFFFAOYSA-N | 469 | C(CC(C(=O)O)N)CC(=O)O | 0.0008 | 3.5 | 469 | 86 | -2.991 | Dicarboxylic Acids |  | 0.12 |
| 4-guanidinobutanoate | TUHVEAJXIMEOSA-UHFFFAOYSA-N | 500 | C(CC(=O)O)CN=C(N)N | 0.0058 | 0.71 | 500 | 63 | -0.946 | Guanidines | e | 0.59 |
| malate | BJEPYKJPYRNKOW-UHFFFAOYSA-N | 525 | C(C(C(=O)O)O)C(=O)O | 0 | 0.25 | 525 | 10 | -1.474 | Dicarboxylic Acids | a | 0 |
| creatine | CVSVTCORWBXHQV-UHFFFAOYSA-N | 586 | CN(CC(=O)O)C(=N)N | 0.023 | 2 | 586 | 66 | -2.404 | Guanidines | f | 1 |
| creatinine | DDRJAANPRJIHGJ-UHFFFAOYSA-N | 588 | CN1CC(=O)N=C1N | 0 | 0.4 | 588 | 66 | -0.126 | Imidazoles | f | 0 |
| cystine | LEVWYRKDKASIDU-UHFFFAOYSA-N | 595 | C(C(C(=O)O)N)SSCC(C(=O)O)N | 0.083 | 2.2 | 595 | 89 | -3.312 | Amino Acids, Sulfur |  | 1 |
| glutamate | WHUUTDBJXJRKMK-VKHMYHEASA-N | 611 | C(CC(=O)O)[C@@H](C(=O)O)N | 0.0014 | 1.4 | 611 | 13 | -3.349 | Glutamates |  | 0.18 |
| lactate | JVTAAEKCZFNVCJ-UHFFFAOYSA-N | 612 | CC(C(=O)O)O | 0.063 | 1.3 | 612 | 38 | -0.591 | Lactates | g | 1 |
| sedoheptulose-7-phosphate | JDTUMPKOJBQPKX-GBNDHIKLSA-N | 616 | C([C@H]([C@H]([C@H]([C@@H]  (C(=O)CO)O)O)O)O)OP(=O)(O)O | 0.0004 | 3.5 | 616 | 9 | -5.74 | Sugar Phosphates | h | 0.064 |
| 5,6-dihydrouracil | OIVLITBTBDPEFK-UHFFFAOYSA-N | 649 | C1CNC(=O)NC1=O | 0 | 3.9 | 649 | 95 | -1.16 | Pyrimidines |  | 0 |
| ethanolamine | HZAXFHJVJLSVMW-UHFFFAOYSA-N | 700 | C(CO)N | 0 | 0.31 | 700 | 26 | -1.275 | Ethanolamines |  | 0 |
| 3-phosphoglycerate | OSJPPGNTCRNQQC-UHFFFAOYSA-N | 724 | C(C(C(=O)O)O)OP(=O)(O)O | 0 | 0.38 | 724 | 68 | -2.952 | Glyceric Acids | c | 0 |
| deoxycarnitine | JHPNVNIEXXLNTR-UHFFFAOYSA-N | 725 | C[N+](C)(C)CCCC(=O)[O-] | 0.056 | 1.4 | 725 | 63 | -0.711 | Carnitine | e | 1 |
| glycerate | RBNPOMFGQQGHHO-UWTATZPHSA-N | 752 | C([C@H](C(=O)O)O)O | 0.0021 | 0.78 | 752 | 38 | -1.723 | Glyceric Acids | g | 0.26 |
| glycerol | PEDCQBHIVMGVHV-UHFFFAOYSA-N | 753 | C(C(CO)O)O | 0.071 | 1.4 | 753 | 14 | -1.88 | Sugar Alcohols |  | 1 |
| guanosine 5'- monophosphate (5'-GMP) | RQFCJASXJCIDSX-UHFFFAOYSA-N | 761 | C1=NC2=C(N1C3C(C(C(O3)COP  (=O)(O)O)O)O)NC(=NC2=O)N | 0 | 5 | 761 | 69 | -2.616 | Guanine Nucleotides | i | 0 |
| guanidinoacetate | BPMFZUMJYQTVII-UHFFFAOYSA-N | 763 | C(C(=O)O)N=C(N)N | 0 | 0.16 | 763 | 66 | -3.974 |  | f | 0 |
| histidine | HNDVDQJCIGZPNO-YFKPBYRVSA-N | 773 | C1=C(NC=N1)C[C@@H](C(=O)O)N | 0 | 0.48 | 773 | 7 | -3.429 | Amino Acids, Cyclic | j | 0 |
| homocysteine | FFFHZYDWPBMWHY-UHFFFAOYSA-N | 778 | C(CS)C(C(=O)O)N | 0.034 | 0.72 | 778 | 89 | -2.217 | Amino Acids, Sulfur |  | 1 |
| hypoxanthine | FDGQSTZJBFJUBT-UHFFFAOYSA-N | 790 | C1=NC2=C(N1)C(=O)N=CN2 | 0 | 0.71 | 790 | 49 | 0.556 | Hypoxanthines | k | 0 |
| isoleucine | AGPKZVBTJJNPAG-WHFBIAKZSA-N | 791 | CC[C@H](C)[C@@H](C(=O)O)N | 0 | 0.58 | 791 | 50 | -1.6 | Amino Acids,  Branched-Chain | l | 0 |
| citrulline | RHGKLRLOHDJJDR-BYPYZUCNSA-N | 833 | C(C[C@@H](C(=O)O)N)CNC(=O)N | 0 | 0.53 | 833 | 11 | -3.909 | Amino Acids |  | 0 |
| myo-inositol | CDAISMWEOUEBRE-UHFFFAOYSA-N | 892 | C1(C(C(C(C(C1O)O)O)O)O)O | 0 | 0.31 | 892 | 31 | -1.458 | Inositol | m | 0 |
| nicotinamide | DFPAKSUCGFBDDF-UHFFFAOYSA-N | 936 | C1=CC(=CN=C1)C(=O)N | 0 | 0.71 | 936 | 61 | -1.236 | Pyridines |  | 0 |
| 1-palmitoylglycerophosphoinositol* | NIOYUNMRJMEDGI-UHFFFAOYSA-N | 984 | CCCCCCCCCCCCCCCC=O | 0 | 0.22 | 984 | 90 | 7.984 | Aldehydes | n | 0 |
| palmitate (16:0) | IPCSVZSSVZVIGE-UHFFFAOYSA-M | 985 | CCCCCCCCCCCCCCCC(=O)[O-] | 0.02 | 1.3 | 985 | 3 | 7.089 | Saturated FA | o | 1 |
| phosphoenolpyruvate (PEP) | DTBNBXWJWCWCIK-UHFFFAOYSA-M | 1005 | C=C(C(=O)O)OP(=O)(O)[O-] | 0 | 0.27 | 1005 | 67 | -1.296 | Carboxylic Acids | p | 0 |
| putrescine | KIDHWZJUCRJVML-UHFFFAOYSA-N | 1045 | C(CCN)CN | 0 | 7.1 | 1045 | 39 | -0.626 | Biogenic Polyamines |  | 0 |
| phosphate | NBIIXXVUZAFLBC-UHFFFAOYSA-K | 1061 | [O-]P(=O)([O-])[O-] | 0 | 0.75 | 1061 | 98 | -2.247 |  | q | 0 |
| citramalate | XFTRTWQBIOMVPK-UHFFFAOYSA-N | 1081 | CC(CC(=O)O)(C(=O)O)O | 0 | 0.42 | 1081 | 91 | -1.364 | Dicarboxylic Acids | d | 0 |
| spermidine | ATHGHQPFGPMSJY-UHFFFAOYSA-N | 1102 | C(CCNCCCN)CN | 0.012 | 1.5 | 1102 | 39 | -0.654 | Putrescine |  | 0.97 |
| succinate | KDYFGRWQOYBRFD-UHFFFAOYSA-N | 1110 | C(CC(=O)O)C(=O)O | 0 | 18 | 1110 | 97 | -0.71 | Dicarboxylic Acids |  | 0 |
| taurine | XOAAWQZATWQOTB-UHFFFAOYSA-N | 1123 | C(CS(=O)(=O)O)N | 0.076 | 0.95 | 1123 | 39 | -1.678 | Alkanesulfonic Acids |  | 1 |
| C-glycosyltryptophan* | QIVBCDIJIAJPQS-UHFFFAOYSA-N | 1148 | C1=CC=C2C(=C1)C(=CN2)CC(C(=O)O)N | 0.097 | 0.88 | 1148 | 36 | -2.023 | Salicylates |  | 1 |
| uracil | ISAKRJDGNUQOIC-UHFFFAOYSA-N | 1174 | C1=CNC(=O)NC1=O | 0.0013 | 0.75 | 1174 | 49 | -0.875 | Pyrimidines | k | 0.17 |
| urea | XSQUKJJJFZCRTK-UHFFFAOYSA-N | 1176 | C(=O)(N)N | 0.038 | 0.37 | 1176 | 39 | -1.686 | Organic Chemicals |  | 1 |
| xanthine | LRFVTYWOQMYALW-UHFFFAOYSA-N | 1188 | C1=NC2=C(N1)C(=O)NC(=O)N2 | 0 | 0.7 | 1188 | 49 | -0.654 | Xanthines | k | 0 |
| 2'-deoxyadenosine 3'-monophosphate | UEUPTUCWIHOIMK-UHFFFAOYSA-N | 1467 | C1C(C(OC1N2C=NC3=C2N=CN=C3N)  CO)OP(=O)(O)O | 0.0032 | 2.4 | 1467 | 17 | -3.424 | Adenine Nucleotides |  | 0.36 |
| 5-oxoETE | MEASLHGILYBXFO-XTDASVJISA-N | 1831 | CCCCC/C=C\C/C=C\C/C=C\C=C\C(=O)  CCCC(=O)O | 0 | 0.06 | 1831 | 1 | 6.351 | oxo-ETE |  | 0 |
| alanylphenylalanine | OMNVYXHOSHNURL-WPRPVWTQSA-N | 2080 | C[C@@H](C(=O)N[C@@H]  (CC1=CC=CC=C1)C(=O)O)N | 0.024 | 2.7 | 2080 | 2 | -1.877 | Dipeptides |  | 1 |
| tyrosylarginine | JXNRXNCCROJZFB-UHFFFAOYSA-N | 2932 | C1=CC(=CC=C1CC(C(=O)NC  (CCCN=C(N)N)C(=O)O)N)O | 0.0037 | 2.6 | 2932 | 4 | -3.774 | Dipeptides |  | 0.4 |
| caprate (10:0) | GHVNFZFCNZKVNT-UHFFFAOYSA-M | 2969 | CCCCCCCCCC(=O)[O-] | 0.025 | 0.85 | 2969 | 3 | 3.675 | Saturated FA | o | 1 |
| sphinganine | OTKJDMGTUTTYMP-UHFFFAOYSA-N | 3126 | CCCCCCCCCCCCCCCC(C(CO)N)O | 0.015 | 1.9 | 3126 | 81 | 6.268 | Neutral Glycosphingolipids | r | 1 |
| guanosine 3'-monophosphate (3'-GMP) | ZDPUTNZENXVHJC-UHFFFAOYSA-N | 3522 | C1=NC2=C(N1C3C(C(C(O3)CO)OP  (=O)(O)O)O)NC(=NC2=O)N | 0 | 0.15 | 3522 | 69 | -3.045 | Guanine Nucleotides | i | 0 |
| heme | KABFMIBPWCXCRK-UHFFFAOYSA-L | 4973 | CC1=C(C2=CC3=NC(=CC4=C(C(=C([N-]4)C=C5C(=C(C(=N5)C=C1[N]2)C=C)  C)C)CCC(=O)O)C(=C3C)CCC(=O)O)C=C.[Fe+2] | 0.0007 | 2.6 | 4973 | 25 | 2.06 | Bile Pigments | s | 0.1 |
| N-acetyl-aspartyl-glutamate (NAAG) | WHUUTDBJXJRKMK-VKHMYHEASA-N | 5255 | C(CC(=O)O)[C@@H](C(=O)O)N | 0 | 0.09 | 5255 | 13 | -3.349 | Glutamates |  | 0 |
| stearate (18:0) | GWOWVOYJLHSRJJ-UHFFFAOYSA-L | 5281 | CCCCCCCCCCCCCCCCCC(=O)O | 0 | 1.6 | 5281 | 3 | 8.708 | Saturated FA | o | 0 |
| thymidine 3'-monophosphate | XXYIANZGUOSQHY-UHFFFAOYSA-N | 5463 | CC1=CN(C(=O)NC1=O)C2CC(C(O2)  CO)OP(=O)(O)O | 0.012 | 2.3 | 5463 | 55 | -2.993 | Thymine Nucleotides |  | 0.97 |
| trigonelline (N'-methylnicotinate) | WWNNZCOKKKDOPX-UHFFFAOYSA-N | 5570 | C[N+]1=CC=CC(=C1)C(=O)[O-] | 0.03 | 0.53 | 5570 | 61 | 0.135 | Alkaloids |  | 1 |
| trans-4-hydroxyproline | PMMYEEVYMWASQN-DMTCNVIQSA-N | 5810 | C1[C@H](CN[C@@H]1C(=O)O)O | 0.007 | 0.53 | 5810 | 58 | -0.786 | Amino Acids, Cyclic | t | 0.69 |
| alanine | QNAYBMKLOCPYGJ-REOHCLBHSA-N | 5950 | C[C@@H](C(=O)O)N | 0 | 0.59 | 5950 | 60 | -2.824 | Amino Acids | u | 0 |
| serine | MTCFGRXMJLQNBG-REOHCLBHSA-N | 5951 | C([C@@H](C(=O)O)N)O | 0 | 0.48 | 5951 | 60 | -3.956 | Amino Acids | u | 0 |
| aspartate | CKLJMWTZIZZHCS-REOHCLBHSA-N | 5960 | C([C@@H](C(=O)O)N)C(=O)O | 0 | 0.4 | 5960 | 92 | -3.707 | Amino Acids, Acidic | v | 0 |
| glutamine | ZDXPYRJPNDTMRX-VKHMYHEASA-N | 5961 | C(CC(=O)N)[C@@H](C(=O)O)N | 0 | 1.6 | 5961 | 83 | -4.077 | Amino Acids, Basic |  | 0 |
| lysine | KDXKERNSBIXSRK-YFKPBYRVSA-N | 5962 | C(CCN)C[C@@H](C(=O)O)N | 0.0001 | 0.67 | 5962 | 86 | -2.949 | Amino Acids, Basic |  | 0.019 |
| fructose | LKDRXBCSQODPBY-VRPWFDPXSA-N | 5984 | C1[C@H]([C@H]([C@@H](C(O1)(CO)O)O)O)O | 0 | 8.4 | 5984 | 21 | -1.74 | Hexoses |  | 0 |
| uridine monophosphate (5' or 3') | DJJCXFVJDGTHFX-XVFCMESISA-N | 6030 | C1=CN(C(=O)NC1=O)[C@H]2[C@@H]  ([C@@H]([C@H](O2)COP(=O)(O)O)O)O | 0.033 | 1.3 | 6030 | 55 | -3.775 | Uracil Nucleotides |  | 1 |
| tyrosine | OUYCCCASQSFEME-QMMMGPOBSA-N | 6057 | C1=CC(=CC=C1C[C@@H](C(=O)O)N)O | 0 | 0.58 | 6057 | 4 | -2.711 | Amino Acids, Aromatic |  | 0 |
| andro steroid monosulfate 2* | AEMFNILZOJDQLW-QAGGRKNESA-N | 6128 | C[C@]12CCC(=O)C=C1CC[C@@H]3  [C@@H]2CC[C@]4([C@H]3CCC4=O)C | 0.0001 | 0.34 | 6128 | 8 | 3.028 | Androstenes |  | 0.019 |
| cytidine | UHDGCWIWMRVCDJ-XVFCMESISA-N | 6175 | C1=CN(C(=O)N=C1N)[C@H]2[C@@H]  ([C@@H]([C@H](O2)CO)O)O | 0.075 | 0.83 | 6175 | 24 | -2.193 | Pyrimidines | w | 1 |
| ornithine | AHLPHDHHMVZTML-BYPYZUCNSA-N | 6262 | C(C[C@@H](C(=O)O)N)CN | 0 | 0.4 | 6262 | 83 | -3.307 | Amino Acids, Basic |  | 0 |
| asparagine | DCXYFEDJOCDNAF-REOHCLBHSA-N | 6267 | C([C@@H](C(=O)O)N)C(=O)N | 0 | 0.48 | 6267 | 92 | -4.435 | Amino Acids, Basic | v | 0 |
| pantothenate | GHOKWGTUZJEAQD-ZETCQYMHSA-N | 6613 | CC(C)(CO)[C@H](C(=O)NCCC(=O)O)O | 0 | 0.2 | 6613 | 51 | -1.343 | beta-Alanine | x | 0 |
| dihydrocholesterol | QYIXCDOBOSTCEI-QCYZZNICSA-N | 6665 | C[C@H](CCCC(C)C)[C@H]1CC[C@@H]  2[C@@]1(CC[C@H]3[C@H]2CC[C@@H]  4[C@@]3(CC[C@@H](C4)O)C)C | 0.001 | 0.75 | 6665 | 22 | 11.783 | Cholesterol |  | 0.14 |
| pyridoxate | HXACOUQIXZGNBF-UHFFFAOYSA-N | 6723 | CC1=NC=C(C(=C1O)C(=O)O)CO | 0 | 0.51 | 6723 | 61 | -0.835 | Pyridines |  | 0 |
| xylitol | HEBKCHPVOIAQTA-NGQZWQHPSA-N | 6912 | C([C@H](C([C@H](CO)O)O)O)O | 0.0001 | 0.55 | 6912 | 14 | -3.224 | Sugar Alcohols |  | 0.019 |
| 5-oxoproline | ODHCTXKNWHHXJC-VKHMYHEASA-N | 7405 | C1CC(=O)N[C@@H]1C(=O)O | 0 | 0.68 | 7405 | 58 | -0.827 | Pyrrolidinones | t | 0 |
| behenate (22:0) | UKMSUNONTOPOIO-UHFFFAOYSA-N | 8215 | CCCCCCCCCCCCCCCCCCCCCC(=O)O | 0.0001 | 1.9 | 8215 | 3 | 10.984 | Saturated FA | o | 0.019 |
| erucate (22:1n9) | DPUOLQHDNGRHBS-KTKRTIGZSA-M | 8216 | CCCCCCCC/C=C\CCCCCCCCCCCC(=O)[O-] | 0 | 4.4 | 8216 | 1 | 9.987 | UnSaturated FA |  | 0 |
| 3-(4-hydroxyphenyl)lactate | JVGVDSSUAVXRDY-UHFFFAOYSA-N | 9378 | C1=CC(=CC=C1CC(C(=O)O)O)O | 0 | 3.9 | 9378 | 36 | -0.478 | Phenols |  | 0 |
| margarate (17:0) | KEMQGTRYUADPNZ-UHFFFAOYSA-N | 10465 | CCCCCCCCCCCCCCCCC(=O)O | 0.0001 | 1.7 | 10465 | 3 | 8.139 | Saturated FA | o | 0.019 |
| 16-hydroxypalmitate | UGAGPNKCDRTDHP-UHFFFAOYSA-M | 10466 | C(CCCCCCCC(=O)[O-])CCCCCCCO | 0 | 0.5 | 10466 | 3 | 5.324 | Saturated FA | o | 0 |
| arachidate (20:0) | VKOBVWXKNCXXDE-UHFFFAOYSA-M | 10467 | CCCCCCCCCCCCCCCCCCCC(=O)[O-] | 0 | 2.5 | 10467 | 3 | 9.365 | Saturated FA | o | 0 |
| N-acetylglycine | OKJIRPAQVSHGFK-UHFFFAOYSA-N | 10972 | CC(=O)NCC(=O)O | 0.0003 | 2 | 10972 | 54 | -0.908 | Dipeptides | y | 0.05 |
| myristate (14:0) | TUNFSRHWOTWDNC-UHFFFAOYSA-N | 11005 | CCCCCCCCCCCCCC(=O)O | 0.014 | 1.5 | 11005 | 3 | 6.432 | Saturated FA | o | 1 |
| 2-hydroxyisobutyrate | BWLBGMIXKSTLSX-UHFFFAOYSA-N | 11671 | CC(C)(C(=O)O)O | 0 | 0.21 | 11671 | 93 | -0.481 | Hydroxybutyrates |  | 0 |
| 2-pyrrolidinone | HNJBEVLQSNELDL-UHFFFAOYSA-N | 12025 | C1CC(=O)NC1 | 0 | 0.2 | 12025 | 64 | -0.312 | Pyrrolidinones |  | 0 |
| nonadecanoate (19:0) | ISYWECDDZWTKFF-UHFFFAOYSA-M | 12591 | CCCCCCCCCCCCCCCCCCC(=O)[O-] | 0 | 3 | 12591 | 3 | 8.796 | Saturated FA | o | 0 |
| dehydroisoandrosterone sulfate (DHEA-S) | CZWCKYRVOZZJNM-USOAJAOKSA-N | 12594 | C[C@]12CC[C@H]3[C@H]([C@@H]1CCC2=O)  CC=C4[C@@]3(CC[C@@H](C4)OS(=O)(=O)O)C | 0.011 | 0.41 | 12594 | 8 | 1.738 | Dehydro-  epiandrosterone |  | 0.95 |
| methylphosphate | CAAULPUQFIIOTL-UHFFFAOYSA-N | 13130 | COP(=O)(O)O | 0.0012 | 0.7 | 13130 | 98 | -1.728 | Organophosphates | q | 0.16 |
| cytidine 5'-diphosphocholine | RZZPDXZPRHQOCG-OJAKKHQRSA-N | 13804 | C[N+](C)(C)CCOP(=O)(O)OP(=O)([O-])OC[C@@H]  1[C@H]([C@H]([C@@H](O1)N2C=CC(=NC2=O)N)O)O | 0.025 | 3.8 | 13804 | 24 | -4.899 | Cytosine Nucleotides | w | 1 |
| pentadecanoate (15:0) | WQEPLUUGTLDZJY-UHFFFAOYSA-N | 13849 | CCCCCCCCCCCCCCC(=O)O | 0.088 | 1.3 | 13849 | 3 | 7.001 | Saturated FA | o | 1 |
| 1-palmitoylglycerol (1-monopalmitin) | QHZLMUACJMDIAE-UHFFFAOYSA-N | 14900 | CCCCCCCCCCCCCCCC(=O)OCC(CO)O | 0.009 | 0.76 | 14900 | 40 | 6.51 | Saturated_Glycerides |  | 0.82 |
| alpha-tocopherol | GVJHHUAWPYXKBD-IEOSBIPESA-N | 14985 | CC1=C(C(=C2CC[C@@](OC2=C1C)  (C)CCC[C@H](C)CCC[C@H](C)CCCC(C)C)C)O | 0 | 12 | 14985 | 30 | 10.695 | Tocopherols | z | 0 |
| gamma-tocopherol | QUEDXNHFTDJVIY-UHFFFAOYSA-N | 14986 | CC1=C(C=C2CCC(OC2=C1C)(C)CCCC(C)  CCCC(C)CCCC(C)C)O | 0 | 5.5 | 14986 | 30 | 10.514 | Tocopherols | z | 0 |
| pseudouridine | PTJWIQPHWPFNBW-GBNDHIKLSA-N | 15047 | C1=C(C(=O)NC(=O)N1)[C@H]2[C@@H]  ([C@@H]([C@H](O2)CO)O)O | 0 | 0.26 | 15047 | 55 | -2.477 | Uridine |  | 0 |
| 4-acetamidobutanoate | UZTFMUBKZQVKLK-UHFFFAOYSA-M | 18189 | CC(=O)NCCCC(=O)[O-] | 0 | 0.3 | 18189 | 64 | -0.873 | Pyrrolidinones |  | 0 |
| 13-HODE + 9-HODE | DTXVKPOKPFWSFF-UHFFFAOYSA-N | 21643 | C1=CC(=NN=C1N)Cl | 0 | 0.23 | 21643 | 96 | 1.131 | Pyridazines | A | 0 |
| 1-stearoylglycerol (1-monostearin) | VBICKXHEKHSIBG-UHFFFAOYSA-N | 24699 | CCCCCCCCCCCCCCCCCC(=O)OCC(CO)O | 0 | 0.41 | 24699 | 40 | 7.648 | Saturated_Glycerides |  | 0 |
| 3-hydroxydecanoate | FYSSBMZUBSBFJL-UHFFFAOYSA-N | 26612 | CCCCCCCC(CC(=O)O)O | 0.0002 | 0.63 | 26612 | 41 | 2.704 | OH-FA_10_0_1 | B | 0.035 |
| adenosine | OIRDTQYFTABQOQ-KQYNXXCUSA-N | 60961 | C1=NC2=C(C(=N1)N)N=CN2[C@H]3[C@@H]  ([C@@H]([C@H](O3)CO)O)O | 0 | 0.24 | 60961 | 17 | -2.367 | Purine Nucleosides |  | 0 |
| 3-aminoisobutyrate | QCHPKSFMDHPSNR-UHFFFAOYSA-N | 64956 | CC(CN)C(=O)O | 0.0026 | 3.7 | 64956 | 59 | -0.733 | Amino Acids |  | 0.31 |
| xanthosine | UBORTCNDUKBEOP-UUOKFMHZSA-N | 64959 | C1=NC2=C(N1[C@H]3[C@@H]([C@@H]  ([C@H](O3)CO)O)O)NC(=O)NC2=O | 0 | 0.34 | 64959 | 6 | -1.843 | Purine Nucleosides |  | 0 |
| glucuronate | AEMOLEFTQBMNLQ-AQKNRBDQSA-N | 65041 | [C@@H]1([C@@H]([C@H](OC([C@@H]1O)O)  C(=O)O)O)O | 0 | 0.1 | 65041 | 10 | -1.54 | Glucuronates | a | 0 |
| 2'-deoxyinosine | VGONTNSXDCQUGY-RRKCRQDMSA-N | 65058 | C1[C@@H]([C@H](O[C@H]1N2C=NC3=C  2NC=NC3=O)CO)O | 0.027 | 2.3 | 65058 | 6 | -0.032 | Purine Nucleosides |  | 1 |
| N-acetylaspartate (NAA) | OTCCIMWXFLJLIA-BYPYZUCNSA-N | 65065 | CC(=O)N[C@@H](CC(=O)O)C(=O)O | 0 | 0.18 | 65065 | 85 | -1.265 | Dipeptides |  | 0 |
| mannose-6-phosphate | NBSCHQHZLSJFNQ-QTVWNMPRSA-N | 65127 | C([C@@H]1[C@H]([C@@H]([C@@H]  (C(O1)O)O)O)O)OP(=O)(O)O | 0 | 5.9 | 65127 | 21 | -3.355 | Hexosephosphates |  | 0 |
| fructose 1-phosphate | ZKLLSNQJRLJIGT-UYFOZJQFSA-N | 65246 | C([C@H]([C@H]([C@@H](C(=O)COP  (=O)(O)O)O)O)O)O | 0 | 8.1 | 65246 | 9 | -5.068 | Hexosephosphates | h | 0 |
| N-acetylserine | JJIHLJJYMXLCOY-BYPYZUCNSA-N | 65249 | CC(=O)N[C@@H](CO)C(=O)O | 0 | 0.52 | 65249 | 82 | -1.514 | Dipeptides |  | 0 |
| cysteinylglycine | ZUKPVRWZDMRIEO-VKHMYHEASA-N | 65270 | C([C@@H](C(=O)NCC(=O)O)N)S | 0 | 6.2 | 65270 | 34 | -3.403 | Dipeptides | C | 0 |
| glutathione, oxidized (GSSG) | YPZRWBKMTBYPTK-UHFFFAOYSA-N | 65359 | C(CC(=O)NC(CSSCC(C(=O)NCC(=O)O)NC(=O)  CCC(C(=O)O)N)C(=O)NCC(=O)O)C(C(=O)O)N | 0.086 | 15 | 65359 | 88 | -6.622 | Oligopeptides | D | 1 |
| N-acetylvaline | IHYJTAOFMMMOPX-LURJTMIESA-N | 66789 | CC(C)[C@@H](C(=O)O)NC(=O)C | 0 | 0.53 | 66789 | 33 | 0.273 | Dipeptides | E | 0 |
| N-acetylarginine | SNEIUMQYRCDYCH-UHFFFAOYSA-N | 67427 | CC(=O)NC(CCCN=C(N)N)C(=O)O | 0.0019 | 3.3 | 67427 | 56 | -3.309 | Dipeptides |  | 0.24 |
| 7-methylurate | YHNNPKUFPWLTOP-UHFFFAOYSA-N | 69160 | CN1C2=C(NC(=O)NC2=O)NC1=O | 0.0005 | 0.05 | 69160 | 70 | -1.478 | Xanthines |  | 0.078 |
| 2-hydroxystearate | KIHBGTRZFAVZRV-UHFFFAOYSA-N | 69417 | CCCCCCCCCCCCCCCCC(C(=O)O)O | 0.0002 | 0.24 | 69417 | 41 | 7.733 | OH-FA_18_0_1 | B | 0.035 |
| fructose-6-phosphate | GSXOAOHZAIYLCY-HSUXUTPPSA-N | 69507 | C([C@H]([C@H]([C@@H](C(=O)CO)O)O)O)  OP(=O)(O)O | 0 | 5.3 | 69507 | 9 | -5.068 | Hexosephosphates | h | 0 |
| 1-methylurate | QFDRTQONISXGJA-UHFFFAOYSA-N | 69726 | CN1C(=O)C2=C(NC(=O)N2)NC1=O | 0.0093 | 0.55 | 69726 | 70 | -1.049 | Xanthines |  | 0.84 |
| 2-eicosatrienoylglycerophosphocholine* | SUHOQUVVVLNYQR-QMMMGPOBSA-N | 71920 | C[N+](C)(C)CCOP(=O)([O-])OC[C@H](CO)O | 0 | 0.73 | 71920 | 42 | -2.928 | Saturated_Lecithins |  | 0 |
| glutamate, gamma-methyl ester | ZGEYCCHDTIDZAE-UHFFFAOYSA-N | 73913 | COC(=O)CCC(C(=O)O)N | 0.0007 | 0.65 | 73913 | 13 | -3.028 | Glutamates |  | 0.1 |
| phenol sulfate | CTYRPMDGLDAWRQ-UHFFFAOYSA-N | 74426 | C1=CC=C(C=C1)OS(=O)(=O)O | 0.0012 | 0.53 | 74426 | 87 | -0.9 | Phenols | F | 0.16 |
| 1-methylimidazoleacetate | MGIKMFCKRKDEIK-UHFFFAOYSA-N | 75810 | CN1C=CN=C1CC(=O)O | 0 | 0.46 | 75810 | 45 | -0.297 | Imidazoles |  | 0 |
| ribulose | ZAQJHHRNXZUBTE-NQXXGFSBSA-N | 79021 | C([C@H]([C@H](C(=O)CO)O)O)O | 0 | 3.2 | 79021 | 10 | -3.167 | Pentoses | a | 0 |
| glucose | WQZGKKKJIJFFOK-GASJEMHNSA-N | 79025 | C([C@@H]1[C@H]([C@@H]([C@H]  (C(O1)O)O)O)O)O | 0 | 4.2 | 79025 | 21 | -1.697 | Hexoses |  | 0 |
| leucylglycine | LESXFEZIFXFIQR-UHFFFAOYSA-N | 79070 | CC(C)CC(C(=O)NCC(=O)O)N | 0.019 | 8.6 | 79070 | 73 | -2.217 | Dipeptides | G | 1 |
| 1-palmitoylglycerophosphocholine | ASWBNKHCZGQVJV-UHFFFAOYSA-O | 86555 | CCCCCCCCCCCCCCCC(=O)OCC(COP(=O)(O)  OCC[N+](C)(C)C)O | 0 | 0.33 | 86555 | 15 | 5.462 | Saturated_  Lysophosphatidyl-cholines | H | 0 |
| tyrosylleucine | AUEJLPRZGVVDNU-STQMWFEESA-N | 87071 | CC(C)C[C@@H](C(=O)O)NC(=O)[C@H]  (CC1=CC=C(C=C1)O)N | 0.0015 | 5.6 | 87071 | 4 | -1.578 | Dipeptides |  | 0.19 |
| N-acetylalanine | KTHDTJVBEPMMGL-UHFFFAOYSA-N | 88064 | CC(C(=O)O)NC(=O)C | 0.0016 | 0.74 | 88064 | 54 | -0.382 | Dipeptides | y | 0.2 |
| glycyltyrosine | XBGGUPMXALFZOT-VIFPVBQESA-N | 92829 | C1=CC(=CC=C1C[C@@H](C(=O)O)NC(=O)CN)O | 0.0003 | 2.5 | 92829 | 4 | -3.539 | Dipeptides |  | 0.05 |
| 2-hydroxypalmitate | JGHSBPIZNUXPLA-UHFFFAOYSA-M | 92836 | CCCCCCCCCCCCCCC(C(=O)[O-])O | 0 | 0.13 | 92836 | 41 | 6.114 | OH-FA_16_0_1 | B | 0 |
| glycylleucine | DKEXFJVMVGETOO-LURJTMIESA-N | 92843 | CC(C)C[C@@H](C(=O)O)NC(=O)CN | 0.0046 | 17 | 92843 | 16 | -2.217 | Dipeptides |  | 0.48 |
| gamma-glutamylglutamate | OWQDWQKWSLFFFR-WDSKDSINSA-N | 92865 | C(CC(=O)N[C@@H](CCC(=O)O)C(=O)O)  [C@@H](C(=O)O)N | 0.0024 | 3.4 | 92865 | 11 | -4.176 | Dipeptides |  | 0.29 |
| indolelactate | XGILAAMKEQUXLS-UHFFFAOYSA-N | 92904 | C1=CC=C2C(=C1)C(=CN2)CC(C(=O)O) | 0 | 2 | 92904 | 20 | 0.974 | Amino Acids, Aromatic | I | 0 |
| N2,N2-dimethylguanosine | RSPURTUNRHNVGF-IOSLPCCCSA-N | 92919 | CN(C)C1=NC(=O)C2=C(N1)N(C=N2)[C@H]  3[C@@H]([C@@H]([C@H](O3)CO)O)O | 0 | 0.37 | 92919 | 6 | 0.068 | Purine Nucleosides |  | 0 |
| aspartylphenylalanine | YZQCXOFQZKCETR-UWVGGRQHSA-N | 93078 | C1=CC=C(C=C1)C[C@@H](C(=O)O)NC(=O)  [C@H](CC(=O)O)N | 0.0008 | 5.9 | 93078 | 2 | -2.76 | Dipeptides |  | 0.12 |
| arabitol | HEBKCHPVOIAQTA-QWWZWVQMSA-N | 94154 | C([C@H](C([C@@H](CO)O)O)O)O | 0 | 0.43 | 94154 | 14 | -3.224 | Sugar Alcohols |  | 0 |
| N1-methylguanosine | UTAIYTHAJQNQDW-KQYNXXCUSA-N | 96373 | CN1C(=O)C2=C(N=C1N)N(C=N2)[C@H]  3[C@@H]([C@@H]([C@H](O3)CO)O)O | 0 | 0.21 | 96373 | 6 | -2.13 | Purine Nucleosides |  | 0 |
| glycylmethionine | PFMUCCYYAAFKTH-YFKPBYRVSA-N | 96757 | CSCC[C@@H](C(=O)O)NC(=O)CN | 0.09 | 1 | 96757 | 34 | -2.681 | Peptides | C | 1 |
| phenylalanylglycine | GLUBLISJVJFHQS-UHFFFAOYSA-N | 98207 | C1=CC=C(C=C1)CC(C(=O)NCC(=O)O)N | 0 | 13 | 98207 | 2 | -2.403 | Dipeptides |  | 0 |
| N-acetylasparagine | HXFOXFJUNFFYMO-BYPYZUCNSA-N | 99715 | CC(=O)N[C@@H](CC(=O)N)C(=O)O | 0.045 | 0.82 | 99715 | 85 | -1.993 | Dipeptides |  | 1 |
| alpha-hydroxyisovalerate | NGEWQZIDQIYUNV-UHFFFAOYSA-N | 99823 | CC(C)C(C(=O)O)O | 0.0009 | 1.7 | 99823 | 93 | 0.064 | Valerates |  | 0.13 |
| 1,2-dipalmitoylglycerol | JEJLGIQLPYYGEE-UHFFFAOYSA-N | 99931 | CCCCCCCCCCCCCCCC(=O)OCC(CO)OC  (=O)CCCCCCCCCCCCCCC | 0.0048 | 0.61 | 99931 | 40 | 14.9 | Saturated_Glycerides |  | 0.5 |
| alpha-glutamylalanine | JZDHUJAFXGNDSB-WHFBIAKZSA-N | 100098 | C[C@@H](C(=O)O)NC(=O)[C@H](CCC(=O)O)N | 0.027 | 3 | 100098 | 11 | -3.651 | Dipeptides |  | 1 |
| sorbitol | FBPFZTCFMRRESA-JGWLITMVSA-N | 107428 | C([C@H]([C@H]([C@@H]([C@H](CO)O)O)O)O)O | 0.06 | 8 | 107428 | 14 | -3.896 | Sugar Alcohols |  | 1 |
| Isobar: fructose 1,6-diphosphate, glucose 1,6-diphosphate, myo-inositol 1,4 or 1,3-diphosphate | GZCGUPFRVQAUEE-SLPGGIOYSA-N | 107526 | C([C@H]([C@H]([C@@H]([C@H](C=O)O)O)O)O)O | 0.0002 | 3.2 | 107526 | 9 | -3.325 | Hexoses | h | 0.035 |
| 7-alpha-hydroxycholesterol | OYXZMSRRJOYLLO-RVOWOUOISA-N | 107722 | C[C@H](CCCC(C)C)[C@H]1CC[C@@H]2  [C@@]1(CC[C@H]3[C@H]2[C@@H](C=C4[C@@]  3(CC[C@@H](C4)O)C)O)C | 0.001 | 0.12 | 107722 | 22 | 8.699 | Hydroxycholesterols |  | 0.14 |
| propionylcarnitine | UFAHZIUFPNSHSL-UHFFFAOYSA-N | 107738 | CCC(=O)OC(CC(=O)[O-])C[N+](C)(C)C | 0.025 | 0.78 | 107738 | 23 | -0.748 | Carnitine |  | 1 |
| phytosphingosine | AERBNCYCJBRYDG-KSZLIROESA-N | 122121 | CCCCCCCCCCCCCC[C@H]([C@H]([C@H](CO)N)O)O | 0.0074 | 1.1 | 122121 | 81 | 5.027 | Neutral Glycosphingolipids | r | 0.72 |
| octanoylcarnitine | CXTATJFJDMJMIY-UHFFFAOYSA-N | 123701 | CCCCCCCC(=O)OC(CC(=O)[O-])C[N+](C)(C)C | 0 | 4.8 | 123701 | 29 | 2.097 | Carnitine | J | 0 |
| pregnanediol-3-glucuronide | ZFFFJLDTCLJDHL-JQYCEVDMSA-N | 123796 | C[C@@H]([C@H]1CC[C@@H]2[C@@  ]1(CC[C@H]3[C@H]2CC[C@H]4[  C@@]3(CC[C@H](C4)O[C@H]5[C@@H]  ([C@H]([C@@H]([C@H](O5)C(=O)O)O)O)O)C)C)O | 0.096 | 0.34 | 123796 | 8 | 5.455 | Pregnanes |  | 1 |
| dimethylarginine (SDMA + ADMA) | NWGZOALPWZDXNG-LURJTMIESA-N | 10176589 | CN(C)[C@@H](CCCN=C(N)N)C(=O)O | 0 | 0.37 | 10176589 | 56 | -2.956 | Dipeptides |  | 0 |
| 3-carboxy-4-methyl-5-propyl-2-furanpropanoate (CMPF) | QAJYCQZQLVENRZ-UHFFFAOYSA-N | 123979 | C1=CC(=NC=C1N)Cl | 0.038 | 2 | 123979 | 30 | 0.044 | Propionates | z | 1 |
| 3-methylglutarylcarnitine (C6) | HFCPFJNSBPQJDP-UHFFFAOYSA-N | 128145 | CC(CC(=O)[O-])CC(=O)OC(CC(=O)O)C[N+](C)(C)C | 0 | 0.21 | 128145 | 80 | -0.765 | Glutarates |  | 0 |
| pyroglutamine* | NPWMTBZSRRLQNJ-UHFFFAOYSA-N | 134508 | C1CC(=O)NC(=O)C1N | 0.054 | 1.9 | 134508 | 64 | -1.014 | Dipeptides |  | 1 |
| choline phosphate | YHHSONZFOIEMCP-UHFFFAOYSA-N | 135437 | C[N+](C)(C)CCOP(=O)(O)[O-] | 0.0002 | 1.9 | 135437 | 94 | -2.066 | Choline | K | 0.035 |
| valylglycine | IOUPEELXVYPCPG-LURJTMIESA-N | 136487 | CC(C)[C@@H](C(=O)NCC(=O)O)N | 0.011 | 8.8 | 136487 | 33 | -2.997 | Dipeptides | E | 0.95 |
| valylserine | STTYIMSDIYISRG-UHFFFAOYSA-N | 139506 | CC(C)C(C(=O)NC(CO)C(=O)O)N | 0.013 | 3.5 | 139506 | 77 | -3.603 | Dipeptides |  | 1 |
| proline | ONIBWKKTOPOVIA-BYPYZUCNSA-N | 145742 | C1C[C@H](NC1)C(=O)O | 0 | 0.45 | 145742 | 58 | -0.185 | Amino Acids, Cyclic | t | 0 |
| 4-hydroxyhippurate | ZMHLUFWWWPBTIU-UHFFFAOYSA-N | 151012 | C1=CC(=CC=C1C(=O)NCC(=O)O)O | 0 | 0.05 | 151012 | 35 | -0.56 | Hippurates | L | 0 |
| gamma-glutamylleucine | MYFMARDICOWMQP-YUMQZZPRSA-N | 151023 | CC(C)C[C@@H](C(=O)O)NC(=O)CC[C@@H]  (C(=O)O)N | 0.0038 | 1.5 | 151023 | 11 | -2.216 | Dipeptides |  | 0.41 |
| Isobar: ribulose 5-phosphate, xylulose 5-phosphate | ZAQJHHRNXZUBTE-NQXXGFSBSA-N | 151261 | C([C@H]([C@H](C(=O)CO)O)O)O | 0 | 5.5 | 151261 | 10 | -3.167 | Pentoses | a | 0 |
| sorbitol 6-phosphate | GACTWZZMVMUKNG-SLPGGIOYSA-N | 152306 | C([C@@H]([C@H]([C@@H]([C@@H]  (COP(=O)(O)O)O)O)O)O)O | 0.014 | 2.2 | 152306 | 31 | -5.125 | Hexosephosphates | m | 1 |
| methionine sulfoxide | QEFRNWWLZKMPFJ-UHFFFAOYSA-N | 158980 | CS(=O)CCC(C(=O)O)N | 0.0001 | 0.52 | 158980 | 79 | -3.882 | Amino Acids, Sulfur | M | 0.019 |
| kynurenine | YGPSJZOEDVAXAB-UHFFFAOYSA-N | 161166 | C1=CC=C(C(=C1)C(=O)CC(C(=O)O)N)N | 0 | 5.9 | 161166 | 2 | -2.868 | Amino Acids |  | 0 |
| N6-carbamoylthreonyladenosine | UNUYMBPXEFMLNW-DWVDDHQFSA-N | 161466 | C[C@H]([C@@H](C(=O)O)NC(=O)NC1=NC=  NC2=C1N=CN2[C@H]3[C@@H]  ([C@@H]([C@H](O3)CO)O)O)O | 0 | 0.42 | 161466 | 6 | -3.42 | Purine Nucleosides |  | 0 |
| mannose | WQZGKKKJIJFFOK-QTVWNMPRSA-N | 161658 | C([C@@H]1[C@H]([C@@H]([C@@H]  (C(O1)O)O)O)O)O | 0.011 | 0.68 | 161658 | 21 | -1.697 | Hexoses |  | 0.95 |
| 2-arachidonoylglycerophosphoinositol* | LXUGKKVCSTYZFK-HYNUQJCBSA-N | 167572 | C(C(COP(=O)(O)OC1[C@H]([C@H](C([C@H]  ([C@@H]1O)O)O)O)O)O)O | 0.0006 | 0.81 | 167572 | 31 | -4.407 | Inositol | m | 0.092 |
| threitol | UNXHWFMMPAWVPI-QWWZWVQMSA-N | 169019 | C([C@H]([C@@H](CO)O)O)O | 0 | 0.47 | 169019 | 14 | -2.552 | Sugar Alcohols |  | 0 |
| choline | CRBHXDCYXIISFC-UHFFFAOYSA-N | 170746 | C[N+](C)(C)CC[O-] | 0 | 0.57 | 170746 | 62 | -0.837 | Ethanolamines |  | 0 |
| N-acetylglutamine | KSMRODHGGIIXDV-YFKPBYRVSA-N | 182230 | CC(=O)N[C@@H](CCC(=O)N)C(=O)O | 0 | 3.9 | 182230 | 11 | -1.635 | Dipeptides |  | 0 |
| 2'-deoxyguanosine | YKBGVTZYEHREMT-KVQBGUIXSA-N | 187790 | C1[C@@H]([C@H](O[C@H]1N2C=NC3=  C2NC(=NC3=O)N)CO)O | 0.06 | 2 | 187790 | 6 | -0.357 | Purine Nucleosides |  | 1 |
| histidylleucine | MMFKFJORZBJVNF-UWVGGRQHSA-N | 189008 | CC(C)C[C@@H](C(=O)O)NC(=O)[C@H]  (CC1=CN=CN1)N | 0 | 11 | 189008 | 7 | -2.296 | Dipeptides | j | 0 |
| butyrylcarnitine | QWYFHHGCZUCMBN-UHFFFAOYSA-N | 213144 | CCCC(=O)OC(CC(=O)[O-])C[N+](C)(C)C | 0.0036 | 1.7 | 213144 | 23 | -0.179 | Carnitine |  | 0.39 |
| cortisone | MFYSYFVPBJMHGN-ZPOLXVRWSA-N | 222786 | C[C@]12CCC(=O)C=C1CC[C@@H]3[C@@H]  2C(=O)C[C@]4([C@H]3CC[C@@]4(C(=O)CO)O)C | 0 | 0.42 | 222786 | 8 | 0.14 | Pregnanes |  | 0 |
| cytidine-3'-monophosphate (3'-CMP) | UOOOPKANIPLQPU-UHFFFAOYSA-N | 256622 | C1=CN(C(=O)N=C1N)C2C(C(C(O2)CO)OP(=O)(O)O)O | 0.0002 | 0.62 | 256622 | 24 | -4.28 | Cytosine Nucleotides | w | 0.035 |
| leucylalanine | HSQGMTRYSIHDAC-UHFFFAOYSA-N | 259321 | CC(C)CC(C(=O)NC(C)C(=O)O)N | 0.0004 | 4.8 | 259321 | 73 | -1.691 | Dipeptides | G | 0.064 |
| tyrosylglycine | HPYDSVWYXXKHRD-VIFPVBQESA-N | 259323 | C1=CC(=CC=C1C[C@@H](C(=O)NCC(=O)O)N)O | 0 | 4.1 | 259323 | 4 | -3.539 | Dipeptides |  | 0 |
| leucylphenylalanine | KFKWRHQBZQICHA-STQMWFEESA-N | 259325 | CC(C)C[C@@H](C(=O)N[C@@H]  (CC1=CC=CC=C1)C(=O)O)N | 0.043 | 1.2 | 259325 | 2 | -0.442 | Dipeptides |  | 1 |
| alanylleucine | RDIKFPRVLJLMER-UHFFFAOYSA-N | 259583 | CC(C)CC(C(=O)O)NC(=O)C(C)N | 0.0008 | 21 | 259583 | 16 | -1.691 | Dipeptides |  | 0.12 |
| tryptophylglycine | UYKREHOKELZSPB-JTQLQIEISA-N | 263471 | C1=CC=C2C(=C1)C(=CN2)C[C@@H]  (C(=O)NCC(=O)O)N | 0 | 3.8 | 263471 | 20 | -2.851 | Dipeptides | I | 0 |
| valylmethionine | YSGSDAIMSCVPHG-UHFFFAOYSA-N | 292427 | CC(C)C(C(=O)NC(CCSC)C(=O)O)N | 0.051 | 1.1 | 292427 | 34 | -1.5 | Dipeptides | C | 1 |
| phenylacetylglutamine | JFLIEFSWGNOPJJ-JTQLQIEISA-N | 306137 | C1=CC=C(C=C1)CC(=O)N[C@@H](CCC(=O)N)C(=O)O | 0 | 0.07 | 306137 | 78 | -0.302 | Dipeptides |  | 0 |
| glycyllysine | IKAIKUBBJHFNBZ-LURJTMIESA-N | 306144 | C(CCN)C[C@@H](C(=O)O)NC(=O)CN | 0 | 6.3 | 306144 | 32 | -3.777 | Dipeptides |  | 0 |
| aspartylleucine | ZVDPYSVOZFINEE-UHFFFAOYSA-N | 332962 | CC(C)CC(C(=O)O)NC(=O)C(CC(=O)O)N | 0 | 12 | 332962 | 19 | -2.574 | Oligopeptides |  | 0 |
| arginylleucine | WYBVBIHNJWOLCJ-UHFFFAOYSA-N | 333445 | CC(C)CC(C(=O)O)NC(=O)C(CCCN=C(N)N)N | 0.011 | 3.5 | 333445 | 56 | -2.452 | Dipeptides |  | 0.95 |
| valylalanine | HSRXSKHRSXRCFC-WDSKDSINSA-N | 334517 | C[C@@H](C(=O)[O-])NC(=O)[C@H](C(C)C)[NH3+] | 0.0066 | 15 | 334517 | 43 | -0.252 | Dipeptides | N | 0.66 |
| phenylalanylaspartate | HWMGTNOVUDIKRE-UHFFFAOYSA-N | 335051 | C1=CC=C(C=C1)CC(C(=O)NC(CC(=O)O)C(=O)O)N | 0.0001 | 9.1 | 335051 | 2 | -2.76 | Dipeptides |  | 0.019 |
| leucylhistidine | XWOBNBRUDDUEEY-UHFFFAOYSA-N | 341684 | CC(C)CC(C(=O)NC(CC1=CN=CN1)C(=O)O)N | 0.0081 | 3.8 | 341684 | 7 | -2.296 | Oligopeptides | j | 0.76 |
| isoleucyltyrosine | MUFXDFWAJSPHIQ-UHFFFAOYSA-N | 342468 | CCC(C)C(C(=O)NC(CC1=CC=C(C=C1)O)C(=O)O)N | 0.059 | 1.7 | 342468 | 4 | -1.789 | Dipeptides |  | 1 |
| isoleucylglycine | UCGDDTHMMVWVMV-FSPLSTOPSA-N | 342532 | CC[C@H](C)[C@@H](C(=O)NCC(=O)[O-])[NH3+] | 0.016 | 6.7 | 342532 | 43 | -0.209 | Dipeptides | N | 1 |
| alpha-glutamyltyrosine | YSWHPLCDIMUKFE-UHFFFAOYSA-N | 351830 | C1=CC(=CC=C1CC(C(=O)O)NC(=O)C(CCC(=O)O)N)O | 0.0067 | 4 | 351830 | 4 | -3.538 | Dipeptides |  | 0.66 |
| valylleucine | XCTHZFGSVQBHBW-IUCAKERBSA-N | 352039 | CC(C)C[C@@H](C(=O)[O-])NC(=O)[C@H](C(C)C)[NH3+] | 0.0013 | 4.8 | 352039 | 16 | 1.183 | Dipeptides |  | 0.17 |
| valylvaline | KRNYOVHEKOBTEF-UHFFFAOYSA-N | 409682 | CC(C)C(C(=O)NC(C(C)C)C(=O)O)N | 0.015 | 2.8 | 409682 | 33 | -1.816 | Dipeptides | E | 1 |
| threonylvaline | CKHWEVXPLJBEOZ-VQVTYTSYSA-N | 416721 | C[C@H]([C@@H](C(=O)N[C@@H](C(C)C)C(=O)[O-])[NH3+])O | 0.025 | 3.8 | 416721 | 51 | -0.924 | Dipeptides | x | 1 |
| N-acetylneuraminate | SQVRNKJHWKZAKO-LUWBGTNYSA-N | 439197 | CC(=O)N[C@@H]1[C@H](CC(O[C@H]1[C@@H]  ([C@@H](CO)O)O)(C(=O)O)O)O | 0 | 4.8 | 439197 | 65 | -3.525 | Sialic Acids |  | 0 |
| maltose | GUBGYTABKSRVRQ-QUYVBRFLSA-N | 439341 | C([C@@H]1[C@H]([C@@H]([C@H]([C@H](O1)O  [C@@H]2[C@H](O[C@H]([C@@H]([C@H]2O)O)O)CO)O)O)O)O | 0 | 85 | 439341 | 18 | -3.663 | Oligosaccharides |  | 0 |
| N-methylglutamate | XLBVNMSMFQMKEY-BYPYZUCNSA-N | 439377 | CN[C@@H](CCC(=O)O)C(=O)O | 0.0097 | 0.94 | 439377 | 13 | -0.793 | Glutamates |  | 0.85 |
| desmosterol | AVSXSVCZWQODGV-DPAQBDIFSA-N | 439577 | C[C@H](CCC=C(C)C)[C@H]1CC[C@@H]2[C@@]  1(CC[C@H]3[C@H]2CC=C4[C@@]3(CC[C@@H](C4)O)C)C | 0.0001 | 0.42 | 439577 | 22 | 9.661 | Hydroxycholesterols |  | 0.019 |
| maltotriose | FYGDTMLNYKFZSV-DZOUCCHMSA-N | 439586 | C([C@@H]1[C@H]([C@@H]([C@H]([C@H](O1)O[C@@H]  2[C@H](O[C@@H]([C@@H]([C@H]2O)O)O[C@@H]  3[C@H](OC([C@@H]([C@H]3O)O)O)CO)CO)O)O)O)O | 0 | 43 | 439586 | 18 | -5.629 | Oligosaccharides |  | 0 |
| 4-hydroxyglutamate | HBDWQSHEVMSFGY-STHAYSLISA-N | 439902 | C([C@@H](C(=O)O)N)[C@H](C(=O)O)O | 0.012 | 2.5 | 439902 | 13 | -4.113 | Glutamates |  | 0.97 |
| inositol 1-phosphate (I1P) | INAPMGSXUVUWAF-LXOASSSBSA-N | 440194 | [C@H]1([C@@H](C([C@H]([C@@H](C1O)O)O)OP(=O)(O)O)O)O | 0 | 0.74 | 440194 | 31 | -3.545 | Inositol | m | 0 |
| glucosamine | MSWZFWKMSRAUBD-IVMDWMLBSA-N | 441477 | C([C@@H]1[C@H]([C@@H]([C@H](C(O1)O)N)O)O)O | 0 | 0.21 | 441477 | 65 | -1.764 | Hexosamines |  | 0 |
| tryptophan betaine | AOHCBEAZXHZMOR-ZDUSSCGKSA-N | 442106 | C[N+](C)(C)[C@@H](CC1=CNC2=CC=CC=C21)C(=O)[O-] | 0.063 | 2 | 442106 | 20 | 0.1 | Amino Acids, Aromatic | I | 1 |
| prolylleucine | ZKQOUHVVXABNDG-IUCAKERBSA-N | 444109 | CC(C)C[C@@H](C(=O)O)NC(=O)[C@@H]1CCCN1 | 0.052 | 1.1 | 444109 | 16 | 0.948 | Dipeptides |  | 1 |
| 3'-dephosphocoenzyme A | KDTSHFARGAKYJN-IBOSZNHHSA-N | 444485 | CC(C)(COP(=O)(O)OP(=O)(O)OC[C@@H]1[C@H]([C@H]([C@@H]  (O1)N2C=NC3=C2N=CN=C3N)O)O)[C@H](C(=O)NCCC(=O)NCCS)O | 0.018 | 0.88 | 444485 | 74 | -5.117 | Adenine Nucleotides |  | 1 |
| arachidonate (20:4n6) | NNDIXBJHNLFJJP-DTLRTWKJSA-N | 444899 | C(CC/C=C\C/C=C\C/C=C\C/C=C\CCCC(=O)O)CCO | 0 | 0.5 | 444899 | 12 | 6.584 | UnSaturated FA |  | 0 |
| docosahexaenoate (DHA; 22:6n3) | MBMBGCFOFBJSGT-KUBAVDMBSA-N | 445580 | CC/C=C\C/C=C\C/C=C\C/C=C\C/C=C\C/C=C\CCC(=O)O | 0.037 | 0.95 | 445580 | 12 | 8.833 | UnSaturated FA |  | 1 |
| palmitoleate (16:1n7) | SECPZKHBENQXJG-FPLPWBNLSA-N | 445638 | CCCCCC/C=C\CCCCCCCC(=O)O | 0.0006 | 2 | 445638 | 1 | 7.054 | UnSaturated FA |  | 0.092 |
| oleate (18:1n9) | ZQPPMHVWECSIRJ-KTKRTIGZSA-M | 445639 | CCCCCCCC/C=C\CCCCCCCC(=O)[O-] | 0.0015 | 2 | 445639 | 1 | 7.711 | UnSaturated FA |  | 0.19 |
| eicosapentaenoate (EPA; 20:5n3) | JAZBEHYOTPTENJ-JLNKQSITSA-N | 446284 | CC/C=C\C/C=C\C/C=C\C/C=C\C/C=C\CCCC(=O)O | 0.068 | 0.79 | 446284 | 12 | 8.022 | UnSaturated FA |  | 1 |
| maltotetraose | LUEWUZLMQUOBSB-ZLBHSGTGSA-N | 446495 | C([C@@H]1[C@H]([C@@H]([C@H]([C@H](O1)O[C@@H]  2[C@H](O[C@@H]([C@@H]([C@H]2O)O)O[C@@H]3  [C@H](O[C@@H]([C@@H]([C@H]3O)O)O[C@@H]4  [C@H](O[C@@H]  ([C@@H]([C@H]4O)O)O)CO)CO)CO)O)O)O)O | 0 | 42 | 446495 | 18 | -7.595 | Oligosaccharides |  | 0 |
| ribose 5-phosphate | PPQRONHOSHZGFQ-LMVFSUKVSA-N | 447634 | C([C@H]([C@H]([C@H](C=O)O)O)O)OP(=O)(O)O | 0 | 3 | 447634 | 9 | -3.882 | Hexosephosphates | h | 0 |
| N-acetylmethionine | XUYPXLNMDZIRQH-LURJTMIESA-N | 448580 | CC(=O)N[C@@H](CCSC)C(=O)O | 0 | 5.8 | 448580 | 34 | 0.589 | Peptides | C | 0 |
| 3-hydroxyhippurate | XDOFWFNMYJRHEW-UHFFFAOYSA-N | 450268 | C1=CC(=CC(=C1)O)C(=O)NCC(=O)O | 0.0008 | 0.09 | 450268 | 35 | -0.56 | Hippurates | L | 0.12 |
| 2-myristoylglycerophosphocholine* | VXUOFDJKYGDUJI-OAQYLSRUSA-N | 460604 | CCCCCCCCCCCCCC(=O)OC[C@H](COP(=O)([O-])OCC[N+](C)(C)C)O | 0.023 | 1.7 | 460604 | 15 | 4.324 | Saturated_  Lysophosphatidyl-  cholines | H | 1 |
| 7-beta-hydroxycholesterol | OYXZMSRRJOYLLO-KGZHIOMZSA-N | 473141 | C[C@H](CCCC(C)C)[C@H]1CC[C@@H]2[C@@]1(CC[C@H]3  [C@H]2[C@H](C=C4[C@@]3(CC[C@@H](C4)O)C)O)C | 0 | 0.25 | 473141 | 22 | 8.699 | Hydroxycholesterols |  | 0 |
| riboflavin (Vitamin B2) | AUNGANRZJHBGPY-SCRDCRAPSA-N | 493570 | CC1=CC2=C(C=C1C)N(C3=NC(=O)NC(=O)C3=N2)C[C@@H]([C@@H]  ([C@@H](CO)O)O)O | 0 | 0.23 | 493570 | 37 | -2.049 | Flavins |  | 0 |
| 1-stearoylglycerophosphocholine | IHNKQIMGVNPMTC-RUZDIDTESA-N | 497299 | CCCCCCCCCCCCCCCCCC(=O)OC[C@H](COP(=O)([O-])OCC[N+](C)(C)C)O | 0 | 0.36 | 497299 | 15 | 6.6 | Saturated_  Lysophosphatidyl-cholines | H | 0 |
| squalene | YYGNTYWPHWGJRM-AAJYLUCBSA-N | 638072 | CC(=CCC/C(=C/CC/C(=C/CC/C=C(/CC/C=C(/CCC=C(C)C)\C)\C)/C)/C)C | 0 | 0.25 | 638072 | 12 | 11.482 | Polyenes |  | 0 |
| flavin adenine dinucleotide (FAD) | VWWQXMAJTJZDQX-UYBVJOGSSA-N | 643975 | CC1=CC2=C(C=C1C)N(C3=NC(=O)NC(=O)C3=N2)C[C@@H]([C@@H]  ([C@@H](COP(=O)(O)OP(=O)(O)OC[C@@H]4[C@H]([C@H]([C@@H]  (O4)N5C=NC6=C5N=CN=C6N)O)O)O)O)O | 0 | 0.18 | 643975 | 37 | -6.285 | Riboflavin |  | 0 |
| glycerophosphorylcholine (GPC) | SUHOQUVVVLNYQR-MRVPVSSYSA-N | 657272 | C[N+](C)(C)CCOP(=O)([O-])OC[C@@H](CO)O | 0 | 3.7 | 657272 | 42 | -2.928 | Saturated_Lecithins |  | 0 |
| trans-urocanate | LOIYMIARKYCTBW-OWOJBTEDSA-M | 736715 | C1=C(NC=N1)/C=C/C(=O)[O-] | 0.056 | 0.85 | 736715 | 7 | -0.619 | Acrylates | j | 1 |
| glycylphenylalanine | JBCLFWXMTIKCCB-UHFFFAOYSA-N | 1549344 | C1=CC=C(C=C1)CC(C(=O)O)NC(=O)CN | 0.0077 | 3.8 | 1549344 | 2 | -2.403 | Dipeptides |  | 0.73 |
| glycylvaline | STKYPAFSDFAEPH-UHFFFAOYSA-N | 2724807 | CC(C)C(C(=O)O)NC(=O)CN | 0.04 | 3.3 | 2724807 | 33 | -2.997 | Dipeptides | E | 1 |
| ranitidine | VMXUWOKSQNHOCA-UKTHLTGXSA-N | 3001055 | CN/C(=C\[N+](=O)[O-])/NCCSCC1=CC=C(O1)CN(C)C | 0.0023 | 0.19 | 3001055 | 35 | 1.282 | Furans | L | 0.28 |
| glycylproline | KZNQNBZMBZJQJO-YFKPBYRVSA-N | 3013625 | C1C[C@H](N(C1)C(=O)CN)C(=O)O | 0 | 6.1 | 3013625 | 52 | -3.427 | Dipeptides |  | 0 |
| ergothioneine | SSISHJJTAXXQAX-UHFFFAOYSA-N | 3032311 | C[N+](C)(C)C(CC1=CNC(=S)N1)C(=O)[O-] | 0 | 5.7 | 3032311 | 7 | -0.319 | Amino Acids, Cyclic | j | 0 |
| glucose-6-phosphate (G6P) | VFRROHXSMXFLSN-KCDKBNATSA-N | 3034794 | C([C@H]([C@@H]([C@@H]([C@H](C=O)O)O)O)O)OP(=O)(O)O | 0 | 5.8 | 3034794 | 9 | -4.554 | Hexosephosphates | h | 0 |
| N2-methylguanosine | SLEHROROQDYRAW-KQYNXXCUSA-N | 3035422 | CNC1=NC(=O)C2=C(N1)N(C=N2)[C@H]3[C@@H]([C@@H]([C@H](O3)CO)O)O | 0 | 0.39 | 3035422 | 6 | -0.495 | Purine Nucleosides |  | 0 |
| 7-alpha-hydroxy-3-oxo-4-cholestenoate (7-Hoca) | SATGKQGFUDXGAX-MYWFJNCASA-N | 3081085 | C[C@H](CCCC(C)C(=O)O)[C@H]1CC[C@@H]2[C@@]1(CC[C@H]  3[C@H]2[C@@H](CC4=CC(=O)CC[C@]34C)O)C | 0.0003 | 3.1 | 3081085 | 8 | 6.337 | Cholestenones |  | 0.05 |
| 17-methylstearate | YETXGSGCWODRAA-UHFFFAOYSA-M | 3083779 | CC(C)CCCCCCCCCCCCCCCC(=O)[O-] | 0 | 3.2 | 3083779 | 3 | 8.735 | Saturated FA | o | 0 |
| catechol sulfate | MZPWKJZDOCIALD-UHFFFAOYSA-N | 3083879 | C1=CC=C(C(=C1)O)OS(=O)(=O)O | 0.095 | 0.72 | 3083879 | 87 | -0.763 | Phenols | F | 1 |
| maltopentaose | FTNIPWXXIGNQQF-DWTFCAFKSA-N | 3710145 | C([C@@H]1[C@H]([C@@H]([C@H]([C@H](O1)O[C@@H]2[C@H]  (O[C@@H]([C@@H]([C@H]2O)O)O[C@@H]3[C@H](O[C@@H]  ([C@@H]([C@H]3O)O)O[C@@H]4[C@H](O[C@@H]([C@@H]  ([C@H]4O)O)O[C@@H]5[C@H](O[C@@H]([C@@H]  ([C@H]5O)O)O)CO)CO)CO)CO)O)O)O)O | 0 | 15 | 3710145 | 18 | -9.561 | Oligosaccharides |  | 0 |
| p-acetamidophenylglucuronide | IPROLSVTVHAQLE-UHFFFAOYSA-N | 4022661 | CC(=O)NC1=CC=C(C=C1)OC2C(C(C(C(O2)C(=O)O)O)O)O | 0.08 | 0.3 | 4022661 | 35 | -0.835 |  | L | 1 |
| valyltyrosine | VEYJKJORLPYVLO-UHFFFAOYSA-N | 4065033 | CC(C)C(C(=O)NC(CC1=CC=C(C=C1)O)C(=O)O)N | 0 | 4 | 4065033 | 4 | -2.358 | Dipeptides |  | 0 |
| phenylalanylproline | WEQJQNWXCSUVMA-UHFFFAOYSA-N | 4069131 | C1CC(N(C1)C(=O)C(CC2=CC=CC=C2)N)C(=O)O | 0 | 8 | 4069131 | 2 | -1.652 | Dipeptides |  | 0 |
| phenylalanylleucine | RFCVXVPWSPOMFJ-STQMWFEESA-N | 4078229 | CC(C)C[C@@H](C(=O)O)NC(=O)[C@H](CC1=CC=CC=C1)N | 0.0001 | 5.5 | 4078229 | 2 | -0.442 | Dipeptides |  | 0.019 |
| phenylalanylvaline | IEHDJWSAXBGJIP-UHFFFAOYSA-N | 4096934 | CC(C)C(C(=O)O)NC(=O)C(CC1=CC=CC=C1)N | 0.003 | 4.5 | 4096934 | 2 | -1.222 | Dipeptides |  | 0.34 |
| threonylphenylalanine | IQHUITKNHOKGFC-MIMYLULJSA-N | 4099799 | C[C@H]([C@@H](C(=O)N[C@@H](CC1=CC=CC=C1)C(=O)[O-])[NH3+])O | 0.003 | 4.2 | 4099799 | 46 | -0.33 | Dipeptides |  | 0.34 |
| leucylasparagine | MLTRLIITQPXHBJ-UHFFFAOYSA-N | 4128305 | CC(C)CC(C(=O)NC(CC(=O)N)C(=O)O)N | 0.053 | 2.1 | 4128305 | 19 | -3.302 | Oligopeptides |  | 1 |
| cysteine-glutathione disulfide | GNTARDAHCXNJEX-ATVXKPNKSA-N | 4247235 | C(CC(=O)N[C@H](C(=O)NCC(=O)O)C(=S)SC[C@@H](C(O)O)N)[C@@H](C(=O)O)N | 0.044 | 6.9 | 4247235 | 88 | -6.774 | Oligopeptides | D | 1 |
| isoleucylasparagine | HZYHBDVRCBDJJV-UHFFFAOYSA-N | 4414300 | CCC(C)C(C(=O)NC(CC(=O)N)C(=O)O)N | 0.026 | 7.3 | 4414300 | 19 | -3.513 | Oligopeptides |  | 1 |
| glutarate (pentanedioate) | JFCQEDHGNNZCLN-UHFFFAOYSA-L | 4418048 | C(CC(=O)[O-])CC(=O)[O-] | 0 | 0.39 | 4418048 | 97 | -1.314 |  |  | 0 |
| threonylleucine | BQBCIBCLXBKYHW-CSMHCCOUSA-N | 4420322 | C[C@H]([C@@H](C(=O)N[C@@H](CC(C)C)C(=O)O)N)O | 0.01 | 7.7 | 4420322 | 47 | -2.363 | Dipeptides |  | 0.87 |
| phenylalanylglutamate | JXWLMUIXUXLIJR-QWRGUYRKSA-N | 4422358 | C1=CC=C(C=C1)C[C@@H](C(=O)N[C@@H](CCC(=O)O)C(=O)O)N | 0.011 | 5.5 | 4422358 | 78 | -2.402 | Dipeptides |  | 0.95 |
| arginylphenylalanine | PQBHGSGQZSOLIR-RYUDHWBXSA-N | 4441256 | C1=CC=C(C=C1)C[C@@H](C(=O)O)NC(=O)[C@H](CCCN=C(N)N)N | 0.001 | 2.6 | 4441256 | 2 | -2.638 | Dipeptides |  | 0.14 |
| histidylphenylalanine | XMAUFHMAAVTODF-STQMWFEESA-N | 4466133 | C1=CC=C(C=C1)C[C@@H](C(=O)O)NC(=O)[C@H](CC2=CN=CN2)N | 0.0001 | 3 | 4466133 | 57 | -2.482 | Dipeptides |  | 0.019 |
| pro-pro-pro | SBVPYBFMIGDIDX-SRVKXCTJSA-N | 4475718 | C1C[C@H](NC1)C(=O)N2CCC[C@H]2C(=O)N3CCC[C@H]3C(=O)O | 0.0093 | 1.7 | 4475718 | 52 | -0.339 | Peptides |  | 0.84 |
| N-acetylthreonine | PEDXUVCGOLSNLQ-WUJLRWPWSA-N | 4651717 | C[C@H]([C@@H](C(=O)O)NC(=O)C)O | 0 | 0.52 | 4651717 | 82 | -1.054 | Dipeptides |  | 0 |
| 4-hydroxyphenylacetate | XQXPVVBIMDBYFF-UHFFFAOYSA-N | 4693933 | C1=CC(=CC=C1CC(=O)O)O | 0 | 0.26 | 4693933 | 36 | 0.117 | Phenols |  | 0 |
| phosphoethanolamine | SUHOOTKUPISOBE-UHFFFAOYSA-N | 5232324 | C(COP(=O)(O)O)N | 0.0025 | 0.7 | 5232324 | 94 | -2.504 | Ethanolamines | K | 0.3 |
| isoleucylalanine | RCFDOSNHHZGBOY-UHFFFAOYSA-N | 5246009 | CCC(C)C(C(=O)NC(C)C(=O)[O-])[NH3+] | 0.0002 | 7.5 | 5246009 | 43 | 0.317 | Dipeptides | N | 0.035 |
| valyllysine | JKHXYJKMNSSFFL-IUCAKERBSA-N | 5253210 | CC(C)[C@@H](C(=O)N[C@@H](CCCCN)C(=O)O)N | 0.024 | 2 | 5253210 | 32 | -2.596 | Dipeptides |  | 1 |
| glycine | DHMQDGOQFOQNFH-UHFFFAOYSA-N | 5257127 | C(C(=O)[O-])[NH3+] | 0.0038 | 0.78 | 5257127 | 74 | -1.131 | Amino Acids |  | 0.41 |
| bilirubin (Z,Z) | BPYKTIZUTYGOLE-IFADSCNNSA-N | 5280352 | CC1=C(NC(=C1CCC(=O)O)CC2=C(C(=C(N2)/C=C\3/C  (=C(C(=O)N3)C)C=C)C)CCC(=O)O)/C=C\4/C(=C(C(=O)N4)C=C)C | 0 | 4.2 | 5280352 | 25 | 2.167 | Bile Pigments | s | 0 |
| prostaglandin E2 | XEYBRNLFEZDVAW-ARSRFYASSA-N | 5280360 | CCCCC[C@@H](/C=C/[C@H]1[C@@H](CC(=O)[C@@H]1C/C=C\CCCC(=O)O)O)O | 0.0027 | 0.27 | 5280360 | 84 | 2.79 | Prostaglandins E |  | 0.31 |
| linoleate (18:2n6) | OYHQOLUKZRVURQ-AVQMFFATSA-N | 5280450 | CCCCC/C=C/C/C=C/CCCCCCCC(=O)O | 0.0009 | 1.7 | 5280450 | 1 | 7.865 | UnSaturated FA |  | 0.13 |
| 6-keto prostaglandin F1alpha | KFGOFTHODYBSGM-ZUNNJUQCSA-N | 5280888 | CCCCC[C@@H](/C=C/[C@H]1[C@@H](C[C@@H]([C@@H]1CC(=O)CCCCC(=O)O)O)O)O | 0.034 | 0.35 | 5280888 | 84 | 1.647 | Prostaglandins F |  | 1 |
| linolenate [alpha or gamma; (18:3n3 or 6)] | DTOSIQBPPRVQHS-PDBXOOCHSA-N | 5280934 | CC/C=C\C/C=C\C/C=C\CCCCCCCC(=O)O | 0.0001 | 2.2 | 5280934 | 1 | 7.538 | UnSaturated FA |  | 0.019 |
| myristoleate (14:1n5) | YWWVWXASSLXJHU-WAYWQWQTSA-N | 5281119 | CCCC/C=C\CCCCCCCC(=O)O | 0.094 | 0.88 | 5281119 | 1 | 5.916 | UnSaturated FA |  | 1 |
| nervonate (24:1n9) | GWHCXVQVJPWHRF-KTKRTIGZSA-M | 5281120 | CCCCCCCC/C=C\CCCCCCCCCCCCCC(=O)[O-] | 0 | 3.3 | 5281120 | 1 | 11.125 | UnSaturated FA |  | 0 |
| cis-vaccenate (18:1n7) | UWHZIFQPPBDJPM-FPLPWBNLSA-N | 5282761 | CCCCCC/C=C\CCCCCCCCCC(=O)O | 0.013 | 1.6 | 5282761 | 1 | 8.192 | UnSaturated FA |  | 1 |
| eicosenoate (20:1n9 or 11) | BITHHVVYSMSWAG-MDZDMXLPSA-N | 5282769 | CCCCCCCC/C=C/CCCCCCCCCC(=O)O | 0 | 6.5 | 5282769 | 1 | 9.33 | UnSaturated FA |  | 0 |
| docosadienoate (22:2n6) | HVGRZDASOHMCSK-AVQMFFATSA-N | 5282807 | CCCCC/C=C/C/C=C/CCCCCCCCCCCC(=O)O | 0 | 5.3 | 5282807 | 1 | 10.141 | UnSaturated FA |  | 0 |
| adrenate (22:4n6) | TWSWSIQAPQLDBP-DOFZRALJSA-N | 5282844 | CCCCC/C=C\C/C=C\C/C=C\C/C=C\CCCCCC(=O)O | 0 | 2.3 | 5282844 | 1 | 9.487 | UnSaturated FA |  | 0 |
| 1-linoleoylglycerol (1-monolinolein) | WECGLUPZRHILCT-HZJYTTRNSA-N | 5283469 | CCCCC/C=C\C/C=C\CCCCCCCC(=O)OCC(CO)O | 0.0001 | 0.55 | 5283469 | 44 | 6.805 | Unsaturated_  Glycerides |  | 0.019 |
| maltohexaose | OCIBBXPLUVYKCH-VCIHZLQYSA-N | 5288409 | C(C1[C@H]([C@@H](C([C@H](O1)O[C@H]2[C@@H](C([C@H]  (OC2CO)O[C@H]3[C@@H](C([C@H](OC3CO)O[C@H]4[C@@H]  (C([C@H](OC4CO)O[C@H]5[C@@H](C([C@H](OC5CO)O[C@H]  6[C@@H](C(C(OC6CO)O)O)O)O)O)O)O)O)O)O)O)O)O)O)O | 0 | 11 | 5288409 | 18 | -11.527 | Oligosaccharides |  | 0 |
| 11-dehydrocorticosterone | FUFLCEKSBBHCMO-KJQYFISQSA-N | 5311364 | C[C@]12CCC(=O)C=C1CC[C@@H]3[C@@H]2C(=O)C[C@]4  ([C@H]3CC[C@@H]4C(=O)CO)C | 0.0003 | 0.44 | 5311364 | 8 | 1.291 | Pregnanes |  | 0.05 |
| 5-dodecenoate (12:1n7) | IJBFSOLHRKELLR-FPLPWBNLSA-N | 5312378 | CCCCCC/C=C\CCCC(=O)O | 0.0003 | 0.7 | 5312378 | 12 | 4.778 | UnSaturated FA |  | 0.05 |
| 10-heptadecenoate (17:1n7) | BXYRAPNURYRQSP-UHFFFAOYSA-N | 5312435 | C1=CC=NC(=C1)C2=CC=C(C=C2)N | 0 | 2.2 | 5312435 | 71 | 0.443 | Pyridines | O | 0 |
| stearidonate (18:4n3) | JIWBIWFOSCKQMA-LTKCOYKYSA-M | 5312508 | CC/C=C\C/C=C\C/C=C\C/C=C\CCCCC(=O)[O-] | 0.0001 | 2.5 | 5312508 | 12 | 6.73 | UnSaturated FA |  | 0.019 |
| 10-nonadecenoate (19:1n9) | BXYRAPNURYRQSP-UHFFFAOYSA-N | 5312513 | C1=CC=NC(=C1)C2=CC=C(C=C2)N | 0 | 3.6 | 5312513 | 71 | 0.443 | Pyridines | O | 0 |
| dihomo-linolenate (20:3n3 or n6) | AHANXAKGNAKFSK-PDBXOOCHSA-N | 5312529 | CC/C=C\C/C=C\C/C=C\CCCCCCCCCC(=O)O | 0.0088 | 1.7 | 5312529 | 1 | 8.676 | UnSaturated FA |  | 0.81 |
| docosatrienoate (22:3n3) | WBBQTNCISCKUMU-PDBXOOCHSA-N | 5312556 | CC/C=C\C/C=C\C/C=C\CCCCCCCCCCCC(=O)O | 0 | 4 | 5312556 | 1 | 9.814 | UnSaturated FA |  | 0 |
| 1-linoleoylglycerophosphocholine | SPJFYYJXNPEZDW-FTJOPAKQSA-O | 5313990 | CCCCC/C=C\C/C=C\CCCCCCCC(=O)OC[C@H](COP(=O)  (O)OCC[N+](C)(C)C)O | 0 | 0.47 | 5313990 | 5 | 5.757 | Unsaturated_  Lysophosphatidyl-cholines | P | 0 |
| bilirubin (E,E)* | BPYKTIZUTYGOLE-IFADSCNNSA-N | 5315454 | CC1=C(NC(=C1CCC(=O)O)CC2=C(C(=C(N2)/C=C\3/C(=C  (C(=O)N3)C)C=C)C)CCC(=O)O)/C=C\4/C(=C(C(=O)N4)C=C)C | 0.0019 | 2.6 | 5315454 | 25 | 2.167 | Bile Pigments | s | 0.24 |
| 2-linoleoylglycerol (2-monolinolein) | IEPGNWMPIFDNSD-HZJYTTRNSA-N | 5365676 | CCCCC/C=C\C/C=C\CCCCCCCC(=O)OC(CO)CO | 0.0002 | 5.1 | 5365676 | 44 | 6.805 | Unsaturated_Glycerides |  | 0.035 |
| 3-hydroxypropanoate | ALRHLSYJTWAHJZ-UHFFFAOYSA-M | 5459847 | C(CO)C(=O)[O-] | 0.0014 | 0.72 | 5459847 | 38 | -1.44 | Glyceric Acids | g | 0.18 |
| 1-arachidonoylglycerophosphoethanolamine* | JZNWSCPGTDBMEW-UHFFFAOYSA-N | 5459861 | C(COP(=O)(O)OC[C@H](CO)O)N | 0.0007 | 0.65 | 5459861 | 26 | -3.366 | Saturated_  Phosphatidyl-ethanolamines |  | 0.1 |
| N1-methyladenosine | QQBGTSSELNKRID-IOSLPCCCSA-N | 5460178 | CN1CN=C2C(=C1N)N=CN2[C@H]3[C@@H]([C@@H]  ([C@H](O3)CO)O)O | 0 | 0.36 | 5460178 | 17 | -0.634 | Purine Nucleosides |  | 0 |
| fumarate | VZCYOOQTPOCHFL-OWOJBTEDSA-L | 5460307 | C(=C/C(=O)[O-])\C(=O)[O-] | 0 | 0.3 | 5460307 | 67 | -1.378 | Dicarboxylic Acids | p | 0 |
| alanylalanine | DEFJQIDDEAULHB-UHFFFAOYSA-N | 5460362 | CC(C(=O)NC(C)C(=O)O)N | 0.073 | 2.3 | 5460362 | 54 | -3.126 | Dipeptides | y | 1 |
| docosapentaenoate (n3 DPA; 22:5n3) | YUFFSWGQGVEMMI-JLNKQSITSA-N | 5497182 | CC/C=C\C/C=C\C/C=C\C/C=C\C/C=C\CCCCCC(=O)O | 0 | 2.6 | 5497182 | 1 | 9.16 | UnSaturated FA |  | 0 |
| isovalerylcarnitine | IGQBPDJNUXPEMT-UHFFFAOYSA-N | 6426851 | CC(C)CC(=O)OC(CC(=O)[O-])C[N+](C)(C)C | 0 | 0.45 | 6426851 | 27 | 0.329 | Carnitine |  | 0 |
| hexanoylcarnitine | VVPRQWTYSNDTEA-UHFFFAOYSA-N | 6426853 | CCCCCC(=O)OC(CC(=O)[O-])C[N+](C)(C)C | 0.0003 | 2.5 | 6426853 | 76 | 0.959 | Carnitine |  | 0.05 |
| 2-methylbutyrylcarnitine (C5) | IHCPDBBYTYJYIL-UHFFFAOYSA-N | 6426901 | CCC(C)C(=O)OC(CC(=O)[O-])C[N+](C)(C)C | 0 | 0.36 | 6426901 | 27 | 0.114 | Carnitine |  | 0 |
| valerylcarnitine | VSNFQQXVMPSASB-UHFFFAOYSA-N | 6426903 | CCCCC(=O)OC(CC(=O)[O-])C[N+](C)(C)C | 0.031 | 0.68 | 6426903 | 76 | 0.39 | Carnitine |  | 1 |
| dihomo-linoleate (20:2n6) | LNAVIIOBBICBIS-NBRVCOCJSA-N | 6439848 | CCCCCCCCCCCCCCC/C=C/C=C/C(=O)O | 0 | 4.4 | 6439848 | 1 | 9.291 | UnSaturated FA |  | 0 |
| stearoyl sphingomyelin | LKQLRGMMMAHREN-YJFXYUILSA-N | 6453725 | CCCCCCCCCCCCCCCCCC(=O)N[C@@H](COP(=O)([O-])OCC[N+](C)(C)C)[C@@H](/C=C/CCCCCCCCCCCCC)O | 0 | 0.38 | 6453725 | 72 | 14.034 | Phosphatidylcholines | Q | 0 |
| xylonate | QXKAIJAYHKCRRA-FLRLBIABSA-N | 6602431 | C([C@H]([C@@H]([C@H](C(=O)O)O)O)O)O | 0 | 0.46 | 6602431 | 10 | -3.067 | Glutarates | a | 0 |
| beta-tocopherol | WGVKWNUPNGFDFJ-DQCZWYHMSA-N | 6857447 | CC1=CC(=C(C2=C1O[C@](CC2)(C)CCC[C@H]  (C)CCC[C@H](C)CCCC(C)C)C)O | 0 | 10 | 6857447 | 30 | 10.514 | Tocopherols | z | 0 |
| tryptophan | QIVBCDIJIAJPQS-VIFPVBQESA-N | 6923516 | C1=CC=C2C(=C1)C(=CN2)C[C@@H](C(=O)O)N | 0 | 0.57 | 6923516 | 20 | -2.023 | Amino Acids, Aromatic | I | 0 |
| phenylalanine | COLNVLDHVKWLRT-QMMMGPOBSA-N | 6925665 | C1=CC=C(C=C1)C[C@@H](C(=O)O)N | 0.0006 | 0.74 | 6925665 | 2 | -1.575 | Amino Acids, Aromatic |  | 0.092 |
| valine | KZSNJWFQEVHDMF-BYPYZUCNSA-N | 6971018 | CC(C)[C@@H](C(=O)O)N | 0 | 0.6 | 6971018 | 50 | -2.169 | Amino Acids,  Branched-Chain | l | 0 |
| threonine | AYFVYJQAPQTCCC-GBXIJSLDSA-N | 6971019 | C[C@H]([C@@H](C(=O)O)N)O | 0 | 0.58 | 6971019 | 60 | -3.496 | Amino Acids | u | 0 |
| N6-acetyllysine | DTERQYGMUDWYAZ-ZETCQYMHSA-N | 6991978 | CC(=O)NCCCC[C@@H](C(=O)O)N | 0 | 0.32 | 6991978 | 32 | -2.673 | Dipeptides |  | 0 |
| methionine | FFEARJCKVFRZRR-UHFFFAOYSA-N | 6992087 | CSCCC(C(=O)O)N | 0 | 0.59 | 6992087 | 79 | -1.853 | Amino Acids, Sulfur | M | 0 |
| gamma-aminobutyrate (GABA) | BTCSSZJGUNDROE-UHFFFAOYSA-N | 6992099 | C(CC(=O)O)CN | 0 | 0.11 | 6992099 | 63 | -0.668 | Aminobutyrates | e | 0 |
| anserine | MYYIAHXIVFADCU-QMMMGPOBSA-N | 6992114 | CN1C=NC=C1C[C@@H](C(=O)O)NC(=O)CCN | 0.085 | 0.47 | 6992114 | 45 | -3.795 | Dipeptides |  | 1 |
| glycylisoleucine | KGVHCTWYMPWEGN-FSPLSTOPSA-N | 6992386 | CC[C@H](C)[C@@H](C(=O)O)NC(=O)CN | 0.044 | 5 | 6992386 | 16 | -2.428 | Dipeptides |  | 1 |
| N2-acetyllysine | VEYYWZRYIYDQJM-ZETCQYMHSA-N | 6992697 | CC(=O)N[C@@H](CCCCN)C(=O)O | 0.072 | 1.4 | 6992697 | 32 | -2.673 | Dipeptides |  | 1 |
| valylphenylalanine | GJNDXQBALKCYSZ-UHFFFAOYSA-N | 6993119 | CC(C)C(C(=O)NC(CC1=CC=CC=C1)C(=O)O)N | 0.028 | 3 | 6993119 | 2 | -1.222 | Dipeptides |  | 1 |
| phenylalanylalanine | MIDZLCFIAINOQN-WPRPVWTQSA-N | 6993123 | C[C@@H](C(=O)O)NC(=O)[C@H](CC1=CC=CC=C1)N | 0.0003 | 3.6 | 6993123 | 2 | -1.877 | Dipeptides |  | 0.05 |
| serylleucine | NFDYGNFETJVMSE-BQBZGAKWSA-N | 7015694 | CC(C)C[C@@H](C(=O)O)NC(=O)[C@H](CO)N | 0.018 | 9.4 | 7015694 | 47 | -2.823 | Dipeptides |  | 1 |
| serylvaline | ILVGMCVCQBJPSH-WDSKDSINSA-N | 7020159 | CC(C)[C@@H](C(=O)O)NC(=O)[C@H](CO)N | 0.014 | 2.3 | 7020159 | 51 | -3.603 | Oligopeptides | x | 1 |
| acetylcarnitine | RDHQFKQIGNGIED-MRVPVSSYSA-N | 7045767 | CC(=O)O[C@H](CC(=O)[O-])C[N+](C)(C)C | 0 | 3.8 | 7045767 | 23 | -1.001 | Carnitine |  | 0 |
| leucine | ROHFNLRQFUQHCH-YFKPBYRVSA-N | 7045798 | CC(C)C[C@@H](C(=O)O)N | 0.0001 | 0.69 | 7045798 | 50 | -1.389 | Amino Acids,  Branched-Chain | l | 0.019 |
| cytidine 5'-monophosphate (5'-CMP) | IERHLVCPSMICTF-XVFCMESISA-L | 7058165 | C1=CN(C(=O)N=C1N)[C@H]2[C@@H]([C@@H]  ([C@H](O2)COP(=O)([O-])[O-])O)O | 0 | 2.3 | 7058165 | 24 | -3.851 | Cytosine Nucleotides | w | 0 |
| pregnen-diol disulfate* | AVQNPDXTQNUWMO-UHFFFAOYSA-N | 7172135 | CC1=C(N=C(S1)NN)C2=CC(=C(C=C2)O)O | 0.087 | 0.68 | 7172135 | 57 | 0.442 |  |  | 1 |
| valylhistidine | BNQVUHQWZGTIBX-IUCAKERBSA-N | 7408624 | CC(C)[C@@H](C(=O)N[C@@H](CC1=CN=CN1)C(=O)O)N | 0.02 | 3.6 | 7408624 | 7 | -3.076 | Dipeptides | j | 1 |
| 2-oleoylglycerophosphocholine* | SULIDBRAXVDKBU-SEYXRHQNSA-O | 9546675 | CCCCCCCC/C=C\CCCCCCCC(=O)OC(CO)COP(=O)(O)OCC[N+](C)(C)C | 0 | 0.54 | 9546675 | 5 | 6.084 | Unsaturated_  Lysophosphatidyl-cholines | P | 0 |
| 2-palmitoylglycerophosphoethanolamine* | NRMPAIZEDWJYJB-UHFFFAOYSA-N | 9547044 | CCCCCCCCCCCCCCCCOCC(COP(=O)(O)OCCN)OC(=O)  CCCCCCCCCCCCCCC | 0 | 0.42 | 9547044 | 28 | 13.721 | Saturated_  Phosphatidyl-ethanolamines |  | 0 |
| 1-stearoylglycerophosphoethanolamine | BBYWOYAFBUOUFP-JOCHJYFZSA-N | 9547068 | CCCCCCCCCCCCCCCCCC(=O)OC[C@H](COP(=O)(O)OCCN)O | 0 | 0.11 | 9547068 | 28 | 6.162 | Saturated_  Lysophospholipids |  | 0 |
| 1-palmitoylglycerophosphoethanolamine | YVYMBNSKXOXSKW-HXUWFJFHSA-N | 9547069 | CCCCCCCCCCCCCCCC(=O)OC[C@H](COP(=O)(O)OCCN)O | 0 | 0.04 | 9547069 | 28 | 5.024 | Saturated_  Lysophospholipids |  | 0 |
| 1-oleoylglycerophosphoethanolamine | PYVRVRFVLRNJLY-MZMPXXGTSA-N | 9547071 | CCCCCCCC/C=C\CCCCCCCC(=O)OC[C@H](COP(=O)(O)OCCN)O | 0 | 0.07 | 9547071 | 5 | 5.646 | Unsaturated_  Lysophosphatidyl-cholines | P | 0 |
| isoleucyltryptophan | BVRPESWOSNFUCJ-UHFFFAOYSA-N | 9797038 | CCC(C)C(C(=O)NC(CC1=CNC2=CC=CC=C21)C(=O)O)N | 0.027 | 1.6 | 9797038 | 20 | -1.101 | Dipeptides | I | 1 |
| phenylalanylserine | ROHDXJUFQVRDAV-UWVGGRQHSA-N | 9859812 | C1=CC=C(C=C1)C[C@@H](C(=O)N[C@@H](CO)C(=O)O)N | 0.0001 | 4.3 | 9859812 | 46 | -3.009 | Dipeptides |  | 0.019 |
| 5-HETE | KGIJOOYOSFUGPC-JGKLHWIESA-N | 9862886 | CCCCC/C=C\C/C=C\C/C=C\C=C\[C@H](CCCC(=O)O)O | 0 | 0.08 | 9862886 | 44 | 6.624 | HETE |  | 0 |
| isoleucylarginine | HYXQKVOADYPQEA-UHFFFAOYSA-N | 9900561 | CCC(C)C(C(=O)NC(CCCN=C(N)N)C(=O)O)N | 0.035 | 5 | 9900561 | 38 | -2.663 | Acetates | g | 1 |
| N-acetylcarnosine | BKAYIFDRRZZKNF-VIFPVBQESA-N | 9903482 | CC(=O)NCCC(=O)N[C@@H](CC1=CN=CN1)C(=O)O | 0 | 0.09 | 9903482 | 7 | -1.657 | Dipeptides | j | 0 |
| epiandrosterone sulfate | ZMITXKRGXGRMKS-LUJOEAJASA-N | 9929317 | C[C@]12CC[C@@H](C[C@@H]1CC[C@@H]3[C@@H]  2CC[C@]4([C@H]3CCC4=O)C)OS(=O)(=O)O | 0.0012 | 0.22 | 9929317 | 8 | 3.003 |  |  | 0.16 |
| palmitoyl sphingomyelin | RWKUXQNLWDTSLO-GWQJGLRPSA-N | 9939941 | CCCCCCCCCCCCCCCC(=O)N[C@@H](COP(=O)([O-])OCC[N+](C)(C)C)[C@@H](/C=C/CCCCCCCCCCCCC)O | 0.0001 | 0.6 | 9939941 | 72 | 12.896 | Neutral Glycosphingolipids | Q | 0.019 |
| N-acetylglucosamine 6-phosphate | BRGMHAYQAZFZDJ-PVFLNQBWSA-N | 10040501 | CC(=O)N[C@@H]1[C@H]([C@@H]([C@H](O[C@@H]1O)  COP(=O)(O)O)O)O | 0.0003 | 2.1 | 10040501 | 65 | -3.146 | Hexosephosphates |  | 0.05 |
| isobutyrylcarnitine | LRCNOZRCYBNMEP-UHFFFAOYSA-N | 10177002 | CC(C)C(=O)OC(CC(=O)[O-])C[N+](C)(C)C | 0.085 | 0.7 | 10177002 | 27 | -0.455 | Carnitine |  | 1 |
| 2-stearoylglycerophosphocholine* | IQGPMZRCLCCXAG-UHFFFAOYSA-N | 10208382 | CCCCCCCCCCCCCCCCCC(=O)OC(CO)COP(=O)([O-])OCC[N+](C)(C)C | 0.0007 | 0.89 | 10208382 | 15 | 6.6 | Saturated_  Lysophosphatidyl-cholines | H | 0.1 |
| decanoylcarnitine | LZOSYCMHQXPBFU-UHFFFAOYSA-N | 10245190 | CCCCCCCCCC(=O)OC(CC(=O)[O-])C[N+](C)(C)C | 0 | 3.1 | 10245190 | 29 | 3.235 | Carnitine | J | 0 |
| N2,N5-diacetylornithine | XUYANFPPYJSBPU-UHFFFAOYSA-N | 10398396 | CC(=O)NCCCC(C(=O)O)NC(=O)C | 0 | 0.36 | 10398396 | 11 | -0.589 | Dipeptides |  | 0 |
| laurylcarnitine | FUJLYHJROOYKRA-UHFFFAOYSA-N | 10427569 | CCCCCCCCCCCC(=O)OC(CC(=O)[O-])C[N+](C)(C)C | 0 | 2.9 | 10427569 | 29 | 4.373 | Carnitine | J | 0 |
| nicotinamide adenine dinucleotide (NAD+) | BAWFJGJZGIEFAR-NNYOXOHSSA-N | 10897651 | C1=CC(=C[N+](=C1)[C@H]2[C@@H]([C@@H]([C@H](O2)  COP(=O)(O)OP(=O)([O])OC[C@@H]3[C@H]([C@H]([C@@H]  (O3)N4C=NC5=C4N=CN=C5N)O)O)O)O)C(=O)N | 0 | 0.72 | 10897651 | 37 | -6.261 | Adenine Nucleotides |  | 0 |
| prolylproline | RWCOTTLHDJWHRS-UHFFFAOYSA-N | 11902928 | C1CC(NC1)C(=O)N2CCCC2C(=O)O | 0.017 | 1.6 | 11902928 | 52 | -0.262 | Dipeptides |  | 1 |
| 15-methylpalmitate (isobar with 2-methylpalmitate) | YTDZFWNQRRMELI-UHFFFAOYSA-N | 11958915 | C1=CC=C(C=C1)C2=NN=C(C=C2)N | 0 | 2.4 | 11958915 | 96 | 1.757 | Pyridazines | A | 0 |
| 1-palmitoylplasmenylethanolamine* | NIOYUNMRJMEDGI-UHFFFAOYSA-N | 12854160 | CCCCCCCCCCCCCCC[C]=O | 0 | 0.19 | 12854160 | 90 | 7.984 | Ketones | n | 0 |
| isoleucylserine | TWVKGYNQQAUNRN-UHFFFAOYSA-N | 14426033 | CCC(C)C(C(=O)NC(CO)C(=O)O)N | 0.042 | 6.3 | 14426033 | 77 | -3.034 | Dipeptides |  | 1 |
| tyrosylhistidine | ZQOOYCZQENFIMC-UHFFFAOYSA-N | 14717809 | C1=CC(=CC=C1CC(C(=O)NC(CC2=CN=CN2)C(=O)O)N)O | 0 | 6.1 | 14717809 | 57 | -3.618 | Oligopeptides |  | 0 |
| adenosine 5'-monophosphate (AMP) | UDMBCSSLTHHNCD-KQYNXXCUSA-L | 15938965 | C1=NC2=C(C(=N1)N)N=CN2[C@H]3[C@@H]([C@@H]  ([C@H](O3)COP(=O)([O-])[O-])O)O | 0.0026 | 1.1 | 15938965 | 17 | -4.025 | Poly A |  | 0.31 |
| adenosine 3'-monophosphate (3'-AMP) | LNQVTSROQXJCDD-KQYNXXCUSA-L | 15938966 | C1=NC2=C(C(=N1)N)N=CN2[C@H]3[C@@H]([C@@H]  ([C@H](O3)CO)OP(=O)([O-])[O-])O | 0 | 0.33 | 15938966 | 17 | -4.454 | Poly A |  | 0 |
| 2-arachidonoylglycerophosphocholine* | IGJKYDBBINVMLH-SNPVRQPZSA-O | 16035470 | CCCCC/C=C\C/C=C\C/C=C\C/C=C\CCCC(=O)OC(CO)COP  (=O)(O)OCC[N+](C)(C)C | 0.0006 | 0.76 | 16035470 | 5 | 6.241 | Unsaturated_  Lysophosphatidyl-cholines | P | 0.092 |
| 1-oleoylglycerophosphocholine | YAMUFBLWGFFICM-PTGWMXDISA-N | 16081932 | CCCCCCCC/C=C\CCCCCCCC(=O)OC[C@H](COP(=O)  ([O-])OCC[N+](C)(C)C)O | 0 | 0.31 | 16081932 | 5 | 6.084 | Unsaturated_  Lysophosphatidyl  cholines | P | 0 |
| asparagylisoleucine | MQLZLIYPFDIDMZ-UHFFFAOYSA-N | 17805101 | CCC(C)C(C(=O)O)NC(=O)C(CC(=O)N)N | 0.013 | 4.2 | 17805101 | 19 | -3.513 | Oligopeptides |  | 1 |
| asparagylleucine | HXWUJJADFMXNKA-UHFFFAOYSA-N | 18218182 | CC(C)CC(C(=O)O)NC(=O)C(CC(=O)N)N | 0.0002 | 8.9 | 18218182 | 19 | -3.302 | Oligopeptides |  | 0.035 |
| gamma-glutamylisoleucine* | XITLYYAIPBBHPX-UHFFFAOYSA-N | 18218207 | CCC(C)C(C(=O)O)NC(=O)C(CCC(=O)N)N | 0 | 0.44 | 18218207 | 48 | -3.155 | Dipeptides |  | 0 |
| glutamine-leucine | ARPVSMCNIDAQBO-UHFFFAOYSA-N | 18218208 | CC(C)CC(C(=O)O)NC(=O)C(CCC(=O)N)N | 0.0034 | 6.5 | 18218208 | 48 | -2.944 | Dipeptides |  | 0.38 |
| threonylisoleucine | LUMXICQAOKVQOB-UHFFFAOYSA-N | 18218246 | CCC(C)C(C(=O)O)NC(=O)C(C(C)O)N | 0.0076 | 2.2 | 18218246 | 47 | -2.574 | Dipeptides |  | 0.73 |
| tyrosylglutamine | UBAQSAUDKMIEQZ-UHFFFAOYSA-N | 19421321 | C1=CC(=CC=C1CC(C(=O)NC(CCC(=O)N)C(=O)O)N)O | 0.037 | 3.1 | 19421321 | 4 | -4.266 | Peptides |  | 1 |
| serylphenyalanine | PPQRSMGDOHLTBE-UHFFFAOYSA-N | 21285384 | C1=CC=C(C=C1)CC(C(=O)O)NC(=O)C(CO)N | 0.0004 | 5.9 | 21285384 | 46 | -3.009 | Oligopeptides |  | 0.064 |
| 2-arachidonoylglycerophosphoethanolamine* | JZNWSCPGTDBMEW-UHFFFAOYSA-N | 22833510 | C(CO)N(CC(CO)O)P(=O)(O)O | 0 | 0.64 | 22833510 | 42 | -3.775 | Saturated_Lecithins |  | 0 |
| tiglyl carnitine | WURBQCVBQNMUQT-RMKNXTFCSA-N | 22833596 | C/C=C(\C)/C(=O)OC(CC(=O)[O-])C[N+](C)(C)C | 0.03 | 0.77 | 22833596 | 23 | -0.004 | Carnitine |  | 1 |
| 2-palmitoleoylglycerophosphocholine* | LFUDDCMNKWEORN-ZXEGGCGDSA-N | 24779461 | CCCCCC/C=C\CCCCCCCC(=O)OC[C@H](COP(=O)  ([O-])OCC[N+](C)(C)C)O | 0 | 2.4 | 24779461 | 5 | 4.946 | Unsaturated_  Lysophosphatidyl-cholines | P | 0 |
| 1-heptadecanoylglycerophosphocholine | SRRQPVVYXBTRQK-XMMPIXPASA-N | 24779463 | CCCCCCCCCCCCCCCCC(=O)OC[C@H](COP(=O)([O-])  OCC[N+](C)(C)C)O | 0 | 0.3 | 24779463 | 15 | 6.031 | Saturated_  Lysophosphatidyl-cholines | H | 0 |
| 2-oleoylglycerophosphoethanolamine* | JZNWSCPGTDBMEW-UHFFFAOYSA-N | 25200464 | C(COP(=O)([O-])OCC(CO)O)[NH3+] | 0 | 0.26 | 25200464 | 26 | -2.832 | Saturated_  Phosphatidyl-ethanolamines |  | 0 |
| 2-eicosapentaenoylglycerophosphoethanolamine* | JZNWSCPGTDBMEW-YFKPBYRVSA-N | 49791955 | C(COP(=O)([O-])OC[C@H](CO)O)[NH3+] | 0 | 0.38 | 49791955 | 26 | -2.832 | Saturated_  Phosphatidyl-ethanolamines |  | 0 |
| stearoylcarnitine | FNPHNLNTJNMAEE-HSZRJFAPSA-N | 52922056 | CCCCCCCCCCCCCCCCCC(=O)O[C@H](CC(=O)[O-])C[N+](C)(C)C | 0 | 0.44 | 52922056 | 29 | 7.787 | Carnitine | J | 0 |
| 1-eicosadienoylglycerophosphocholine* | YYQVCMMXPIJVHY-ZOIJLGJPSA-N | 52924053 | CCCCC/C=C\C/C=C\CCCCCCCCCC(=O)OC[C@H](COP(=O)([O-])OCC[N+](C)(C)C)O | 0.0053 | 0.8 | 52924053 | 5 | 6.895 | Unsaturated_  Lysophosphatidyl  cholines | P | 0.54 |
| 1-linoleoylglycerophosphoethanolamine* | DBHKHNGBVGWQJE-USWSLJGRSA-N | 52925130 | CCCCC/C=C\C/C=C\CCCCCCCC(=O)OC[C@H](COP(=O)(O)OCCN)O | 0 | 0.19 | 52925130 | 36 | 5.319 | Phenols |  | 0 |
| 1-heptadecanoylglycerophosphoethanolamine* | RVNBVQKDPQVSOY-OAQYLSRUSA-N | 52925149 | CCCCCCCCCCCCCCCCC(=O)OC[C@H](COP(=O)(O)OCCN)O | 0 | 0.1 | 52925149 | 28 | 5.593 | Saturated_  Lysophospholipids |  | 0 |
| N-acetyl-1-methylhistidine* | GVRCKHXHWSYDEF-QMMMGPOBSA-N | 53859791 | CC(=O)N[C@@H](CC1=CN(C=N1)C)C(=O)O | 0 | 0.2 | 53859791 | 45 | -0.369 | Dipeptides |  | 0 |
| ascorbate (Vitamin C) | CIWBSHSKHKDKBQ-JLAZNSOCSA-N | 54670067 | C([C@@H]([C@@H]1C(=C(C(=O)O1)O)O)O)O | 0.0081 | 3.1 | 54670067 | 67 | -0.178 | Ascorbic Acid | p | 0.76 |
| hydroxyisovaleroyl carnitine | DHRNMNHPSWBJEN-UHFFFAOYSA-N | 57357187 | CC(C)CC(=O)OC(CC(=O)[O-])(C[N+](C)(C)C)O | 0 | 0.37 | 57357187 | 27 | -0.249 | Carnitine |  | 0 |
| biliverdin | RCNSAJSGRJSBKK-LJMVIHTPSA-N | 66588766 | CC1=C(/C(=C/C2=C(C(=C(N2)/C=C\3/C(=C(C(=O)N3)C)  C=C)C)CCC(=O)O)/NC1=CC4=NC(=O)C(=C4C)C=C)CCC(=O)O | 0 | 4 | 66588766 | 25 | 2.708 | Bile Pigments | s | 0 |
| glutarylcarnitine (C5) | NXJAXUYOQLTISD-UHFFFAOYSA-N | 71464488 | C[N+](C)(C)CC(CC(=O)[O-])OC(=O)CCCC(=O)O | 0 | 0.32 | 71464488 | 80 | -1.273 | Glutarates |  | 0 |
| 1-stearoylglycerophosphoinositol | MXAFDFDAIFZFET-QIEOIKHQSA-N | 71581135 | CCCCCCCCCCCCCCCCCC(=O)OC[C@H](COP(=O)(O)OC1[C@@H]  ([C@H](C([C@H]([C@H]1O)O)O)O)O)O | 0 | 0.28 | 71581135 | 53 | 5.121 | Phosphatidylinositols |  | 0 |
| 1-oleoylglycerophosphoinositol* | UGDOFRYHDCDVHD-GFMLYYSJSA-N | 86289645 | CCCCCCCC/C=C\CCCCCCCC(=O)OC[C@H](COP(=O)(O)OC1  [C@@H]([C@H](C([C@H]([C@H]1O)O)O)O)O)O | 0 | 0.32 | 86289645 | 53 | 4.605 | Phosphatidylinositols |  | 0 |
| 1-arachidonoylglycerophosphoinositol* | LXUGKKVCSTYZFK-VUSDMFPISA-N | 86290034 | CCCCC/C=C\C/C=C\C/C=C\C/C=C\CCCC(=O)OC[C@H]  (COP(=O)(O)OC1[C@@H]([C@H](C([C@H]([C@H]1O)O)O)O)O)O | 0.0035 | 0.78 | 86290034 | 53 | 4.762 | Phosphatidylinositols |  | 0.38 |
| isoleucylglutamate | KTGFOCFYOZQVRJ-UHFFFAOYSA-M | 131750771 | CCC(C)C(C(=O)NC(CCC(=O)O)C(=O)[O-])N | 0.025 | 3.4 | 131750771 | 48 | -2.908 | Dipeptides |  | 1 |
| tyrosylglutamate | PDSLRCZINIDLMU-UHFFFAOYSA-M | 131750789 | C1=CC(=CC=C1CC(C(=O)NC(CCC(=O)O)C(=O)[O-])N)O | 0.0051 | 3.4 | 131750789 | 4 | -4.019 | Peptides |  | 0.53 |
| 2-linoleoylglycerophosphocholine* | XFUBHUALKATHPX-XVTLYKPTSA-O | 131770412 | CCCCCC/C=C\C/C=C\CCCCCCCC(=O)OC(CO)COP(=O)  (O)OCC[N+](C)(C)C | 0.0004 | 0.69 | 131770412 | 5 | 6.326 | Unsaturated_  Lysophosphatidyl-cholines | P | 0.064 |

**Supplementary Table 2: ChemRich results**
